# Randomized phase 3 trial of Ropeginterferon alfa-2b versus surveillance after tyrosine kinase inhibitor discontinuation in chronic myeloid leukemia (ENDURE/CML-IX)

**DOI:** 10.1038/s41375-025-02859-1

**Published:** 2026-01-12

**Authors:** Andreas Burchert, Franck E. Nicolini, Philipp le Coutre, Susanne Saussele, Andreas Hochhaus, Evin Tuere, Stephan K. Metzelder, Kim Pauck, Holger Garn, Hartmann Raifer, Magdalena Huber, Norbert Gattermann, Martina Crysandt, Philippe Schafhausen, Matthias Bormann, Markus P. Radsak, Agnès Guerci-Bresler, Thomas Illmer, Maria E. Goebeler, Peter Herhaus, Lino L. Teichmann, Georg-Nikolaus Franke, Fabian Lang, Stefan W. Krause, Robert Möhle, Martine Klausmann, Frank Stegelmann, Christoph Lutz, Gabriel Etienne, Andrea Stoltefuß, Joachim R. Göthert, Thomas Ernst, Maisun Abu-Samra, Heinz-Gert Höffkes, Andreas Neubauer, Ying Wang, Paul Weiland, Clara Otto, Lea Kiessler, Theresa Weber, Lea Kroning, Andrea Nist, Thorsten Stiewe, Rüdiger Hehlmann, Behnaz Aminossadati, Michael Wittenberg, Kerstin Winterstein, Thomas Oellerich, Marcus Lechner, Markus Pfirrmann, Carmen Schade-Brittinger, Paul Klemm, Christian Michel

**Affiliations:** 1 Department of Hematology and Oncology, University Hospital Giessen and Marburg, Marburg University, Marburg, Germany; 2 Centre Hospitalier Lyon Sud, Lyon, Cedex 03 France; 3 Department of Hematology, Oncology and Tumor Immunology, Charité Campus Mitte, Charité-Universitätsmedizin Berlin, corporate member of Freie Universität Berlin and Humboldt-Universität zu Berlin, Berlin, Germany; 4 Clinic for Hematology and Oncology, University Heidelberg, Mannheim, Germany; 5 Hematology/Oncology, Jena University Hospital, Jena, Germany; 6 Translational Inflammation Research Division & Core Facility for Single Cell Multiomics, University Marburg, Marburg, Germany; 7 Institute of Systems Immunology, University Marburg, Marburg, Germany; 8 Department of Hematology, Oncology and Clinical Immunology, Heinrich Heine University, Düsseldorf, Germany; 9 Department of Hematology, Oncology, Hemostaseology, and Stem Cell Transplantation, Faculty of Medicine, University Hospital RWTH Aachen, Aachen, Germany; 10 Department of Internal medicine II, University Medical Centre of Hamburg-Eppendorf, Hamburg, Germany; 11 Klinikum Bremen Mitte, Bremen, Germany; 12 Department of Internal Medicine III, University Medical Center, Johannes Gutenberg-University of Mainz, Mainz, Germany; 13 Service d’Hématologie, Centre Hospitalier Universitaire Brabois, Vandoeuvre- lès-, Nancy, France; 14 Group Practice for Hematology and Oncology, Dresden, Germany; 15 CCC Trial Office, Würzburg, Germany; 16 Internal Medicine III, School of Medicine, Klinikum rechts der Isar, Technische Universität München, Munich, Germany; 17 Center for Integrated Oncology Aachen-Bonn-Cologne-Düsseldorf (CIO ABCD), Bonn, Germany; 18 Department for Hematology, Cell Therapy and Hemostaseology, University of Leipzig Medical Center, Leipzig, Germany; 19 Dept. of Medicine, Hematology and Oncology, Goethe University Frankfurt, Frankfurt, Germany; 20 Uniklinikum Erlangen, Medicine 5, Erlangen, Germany; 21 Department of Hematology and Oncology, University of Tübingen, Tübingen, Germany; 22 Practice for Hematology, Oncology and Gastroeneterology, Aschaffenburg, Germany; 23 Department of Internal Medicine III, University Hospital of Ulm, Ulm, Germany; 24 Praxis for Hematology and Oncology, Koblenz, Germany; 25 Groupe Fi-LMC, Institut Bergonié, Bordeaux, France; 26 Evangelisches Krankenhaus Hamm, Hamm, Germany; 27 Department of Hematology and Stem Cell Transplantation, West German Cancer center, University Hospital Essen, University of Duisburg-Essen, Essen, Germany; 28 Department of Hematology and Oncology, University Hospital Giessen and Marburg, Giessen, Germany; 29 Tumorklinik (Medizinische Onkologie, Palliativmedizin, Hämatologie und Hämostaseologie), Klinikum Fulda, Fulda, Germany; 30 Genomics Core Facility, Institute of Molecular Oncology, Member of the German Center for Lung Research (DZL), Philipps University Marburg, Marburg, Germany; Institute of Lung Health (ILH), Justus Liebig University, Giessen, 35392 Germany; 31 Medizinische Fakultät Mannheim, Universität Heidelberg, Mannheim, Germany, and ELN-Foundation, Weinheim, Germany; 32 Marburg University, Faculty of Medicine, Coordinating Center for Clinical Trials, Marburg, Germany; 33 Center for Synthetic Microbiology, SYNMIKRO, University Marburg, Marburg, Germany; 34 Institut für Medizinische Informationsverarbeitung, Biometrie und Epidemiologie (IBE), Medizinische Fakultät, Ludwig-Maximilians-Universität, Munich, Germany

**Keywords:** Randomized controlled trials, Chronic myeloid leukaemia

## Abstract

Treatment-free remission (TFR) after discontinuation of ABL tyrosine kinase inhibitors (TKIs) is an important therapeutic goal in chronic myeloid leukemia (CML). Interferon-α (IFN) has been suggested to promote durable TFR. The phase 3 ENDURE trial (NCT03117816; EUDRA-CT 2016-001030-94) prospectively tested this hypothesis in patients with stable deep molecular remission after TKI therapy. A total of 203 patients were randomised 1:1 to receive ropeginterferon alfa-2b (ropeg-IFN; 100 µg subcutaneously every two weeks for 15 months, n = 95) or observation alone (n = 108) after TKI discontinuation. The primary endpoint was molecular relapse-free survival (MRFS), defined as time to loss of major molecular response (MMR) or death. At a median follow-up of 36 months, 25-month MRFS was 56% (95% confidence interval (CI), 45–66) with ropeg-IFN and 59% (95% CI, 49–68) with observation (hazard ratio (HR), 1.02; 95% CI, 0.68–1.55; P = 0.91). Among 83 patients with molecular data after TKI restart, 79 (95%) regained at least MMR, 78 within 12 months (median 3 months, interquartile range: 2-4 months). Ropeg-IFN was well tolerated (median administered dose of 92 µg, range 3–104), and no new safety signals were observed. Ropeg-IFN maintenance did not improve the probability of sustained TFR after TKI discontinuation.

## Introduction

Tyrosine kinase inhibitor (TKI) therapy with imatinib has normalized survival expectations for patients with chronic-phase chronic myeloid leukemia (CML) [1, 2]. However, TKIs frequently fail to eradicate CML stem cells, necessitating lifelong treatment for the majority of patients [3, 4]. This includes the need for switching of TKI in case of toxicity or the development of TKI resistance [5].

TKI-induced remissions in CML are due to the inhibition of the causative oncogenic kinase, BCR::ABL1. In contrast, the anti-leukemic effects of interferon-alpha (IFN) in CML [6] are pleiotropic. IFN activates the JAK-STAT-signaling pathway in immune cells [7–9] and regulates elicitation of anti-leukemic immune responses [10–13]. Combination strategies involving TKI and IFN have therefore been proposed to harness the complementary anti-leukemic effects of both agents. These approaches have been shown to accelerate the achievement of deep molecular remission [14–17], which is a key prerequisite for TFR eligibility [5, 18]. Moreover, we have previously postulated that higher rates of treatment-free remission (TFR) might be achievable through maintenance with pegylated IFN after TKI discontinuation [12, 19, 20]. However, this hypothesis had not been tested or confirmed in a controlled clinical trial prior to ENDURE.

While TKI discontinuation in eligible patients has consistently resulted in long-term TFR rates of around 50% [21–30], the underlying biological mechanisms of TFR remain largely unclear. They are multifactorial [31], but are presumed to involve immunological control of residual CML [32–36]. As TKI treatment has been linked to a normalization of the immune effector cell composition [37], IFN—known for its pleiotropic immune-stimulatory properties [7]—was hypothesized to more effectively engage the immune system after prior TKI exposure, potentially enhancing the likelihood of sustained TFR. This premise was tested in the randomized, phase 3 ENDURE trial (NCT03117816). In this interventional TFR study, patients with CML in deep molecular remission suitable to discontinue TKI therapy were randomly assigned to receive pegylated interferon alfa-2b (ropeg-IFN) for 15 months or undergo surveillance [38, 39].

## Subjects and methods

### Patients

For this open-label, randomized trial, we enrolled adults with CML in France (3 centers) and Germany (24 centers). Eligible patients had *BCR::ABL1*-positive chronic phase CML and were receiving treatment with any TKI. Patients were required to have a minimum TKI treatment duration at the time of randomization of three years, and a minimum duration of deep molecular remission (DMR) of one year. DMR was defined as detectable *BCR::ABL1* ( ≤ 0.01% on the International Scale [40]) or undetectable *BCR::ABL1* in samples with 10 000 or more *ABL1* transcripts or 24 000 or more *GUS* transcripts. Patients were required to have three PCR results confirming DMR within 12 months prior to study entry, with no results falling below MR4 during that period. An exposure to IFN prior to study entry was not allowed. Patients with a first or second discontinuation attempt could be included. A prior history of TKI resistance was also not an exclusion criterion (see complete inclusion/exclusion criteria available online in the Data Supplement).

### Study design and treatment

ENDURE is an international phase 3 trial (registered with ClinicalTrials.gov ID: NCT03117816 and EUDRA-CT: 2016-001030-94). The study was approved by the institutional ethics committee of the Philipps University Marburg and at each participating center. Ropeg-IFN was provided by AOP Health (Vienna, Austria). All patients gave written informed consent at the time of enrollment. In the consent form, patients could opt for additional participation in translational biomarker studies, which required additional blood sampling at randomization and regular intervals thereafter. The full protocol of this trial is available online (Data Supplement). All investigators had access to all data and have confirmed its accuracy as well as complete adherence to the study protocol.

Eligible patients were randomized in a 1:1 ratio to receive either ropeg-IFN or no further treatment after TKI discontinuation. Randomization was stratified according to the trial site and prior failure of a discontinuation attempt (yes/no). Patients in the experimental arm received 50 μg ropeg-IFN subcutaneously (s.c.) every 2 weeks for the first month and 100 μg ropeg-IFN s.c. every 2 weeks thereafter up to month 15. In case of a loss of MMR, TKI treatment was resumed without delay.

### Study endpoints and assessments

The primary efficacy endpoint was molecular relapse-free survival (MRFS) and was analysed as a time-to-event variable. MRFS was defined as the time from randomization to molecular relapse, which is defined as a loss of major molecular remission (MMR), which is any increase of the *BCR::ABL1* transcript level to >0.1% according to the international scale (IS) or to death from any cause. Accelerated disease and blast crisis implied a prior loss of MMR and were counted as events. A restart of TKI without a prior loss of MMR was censored at the time of restart. Survivors without an event or restart prior to loss of MMR were censored on the last date they were known to be alive. Secondary endpoints can be found in the protocol and included overall survival, safety and tolerability of ropeg-IFN maintenance, quality of life after TKI stop and assessment of immunological biomarkers associated with TFR (Data Supplement). Safety was assessed in 202 of the 203 patients (safety population), excluding one randomized ropeg-IFN patient, who did not receive at least one ropeg-IFN dose.

Data entry lock for the primary analysis was June 2022.

### Sample size calculation

Assuming an exponential distribution, hazard rates for the primary endpoint MRFS were 0.0529 for the control arm and 0.0297 for the experimental arm. With an accrual time of 25 months after randomization, a minimum follow-up time of 7 months, a drop-out rate of 5%, and 1:1 randomization, a sample size of 210 patients would provide 80% power to detect a significant difference between the hazard rates at a two-sided significance level of 0.05.

### Statistical analysis

MRFS probabilities over time were described using Kaplan-Meier estimates. The MRFS probabilities between the two treatment arms were compared with the log-rank test. As randomization was stratified by prior failure of a discontinuation attempt, the influence of stratum and a potential interaction between treatment arm and stratum were examined using a stratified log-rank test and multiple Cox regression modelling. MRFS was also analysed at the fixed times 6, 12, and 24 months after TKI discontinuation, corresponding to 7, 13, and 25 months after randomization, due to a one-month overlap of treatment with ropeg-IFN and TKI prior to TKI discontinuation.

Overall survival (OS) was defined as the time from randomization to death from any cause or censoring at the last time the patient was known to be alive. OS was estimated using Kaplan-Meier analysis.

Except for the analysis of the primary endpoint and the potential testing of the hierarchically ordered secondary endpoints MRFS at 7, 13, and 25 months after randomization, all statistical analyses were exploratory. Due to reduced sample sizes over time, in case of the three ordered secondary endpoints, one-sided tests were performed, with the null hypotheses that the results in the experimental arm would be worse. Unless specified otherwise, *P* values were not adjusted for multiple testing. For all tests, the significance level was set at 0.05. Point estimations are given together with their 95%-CI.

The software for analysis was SAS version 9.4 (SAS Institute, Cary, NC) and R version 4.5.1 (R Foundation for Statistical Computing, Vienna, Austria).

## Results

### Patients

**Fig. 1:**
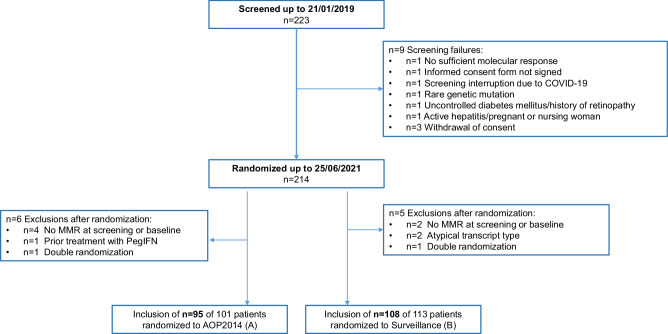
CONSORT flow diagram of patient disposition in the ENDURE trial. Of 223 patients screened, 9 (4.0%) did not meet the eligibility criteria. A total of 214 patients (96%) were randomized to either ropeginterferon alfa-2b (AOP2014; *n* = 101) or surveillance (*n* = 113). After randomization, 11 patients (4.9%) were excluded from the analysis. The final analysis population comprised 95 patients in the ropeg-IFN arm and 108 patients in the surveillance arm. COVID coronavirus disease, MMR major molecular remission, PegIFN pegylated interferon.

Between May 2017 and June 2021, 223 patients entered screening. Overall, 203 patients (68 females, 135 males) with a median age of 55 years (range 20-88) were randomly assigned to ropeg-IFN maintenance after TKI stop (*n* = 95) versus surveillance only after TKI stop (*n* = 108) (Fig. 1). For 77 patients in the ropeg-IFN arm (81%) and 86 patients in the surveillance arm (80%), it was the first TKI discontinuation attempt (Table 1). The median TKI treatment duration prior to TKI stop was 7.8 years (range, 2.5 –19,7) and the proportion of ELTS-high risk patients [41] was comparable in both arms and 15% for the entire cohort (Table 1). Median duration of latest stable MR4 or better was 3 years (inter quartile range (IQR): 2–5 years). At the time of data cut off in June 2022, the median observation time for all patients was 36 months (IQR: 25–48).

**Table 1:** Baseline characteristics and pretreatment of CML patients.

WHO grade 3 and 4 adverse events, *n* (%)	Ropeg-IFN (*n* = 94^a^)		Surveillance (*n* = 108)	
	Grade 3	Grade 4	Grade 3	Grade 4
Neutropenia	3 (3.2)	-	1 (0.9)	-
Pain (bone, joint and skeletal, head, general)	5 (5.3)	-	6 (5.6)	-
Myalgia	-	-	2 (1.9)	-
Hypertriglyceridemia	2 (2.1)	1 (1.1)	1 (0.0)	
Liver enzyme elevation	1 (1.1)	1 (1.1)	-	-
Gastrointestinal toxicity (nausea, diarrhea, dry mouth)	2 (2.1)	-	1 (0.9)	
Skin (lichen, erysipelas)	1 (1.1)	-	1 (0.9)	-
Neurological (hearing loss, PNP, insomnia, hypoasthesia)	4 (4.3)	-	1 (0.9)	
Edema	-	-	2 (1.9)	-
Fatigue, discomfort	-	-	2 (1.9)	-
Arterial disorder and hypertension	2 (2.1)	-	7 (6.5)	-
Cardiopulmonary (pleural effusion)	2 (2.1)	-	-	-
Other (operations, bone fracture, COVID-19, hyperthyrodism, hyponatremia, adenoma, sinusitis)	6 (6.4)	-	3 (2.8)	-
Total 30 (31.9) 2 (2.1) 26 (24.1)				

All data are presented as no. (%) unless otherwise indicated.

*CML* chronic myeloid leukemia, *ELTS* EUTOS long-term survival score, *EUTOS* European Treatment and Outcome Study, *ropeg-IFN* ropeginterferon alfa-2b, *TKI* tyrosine kinase inhibitor.

^a^Safety population (patients, who received at least one time study medication).

### Efficacy

**Fig. 2:**
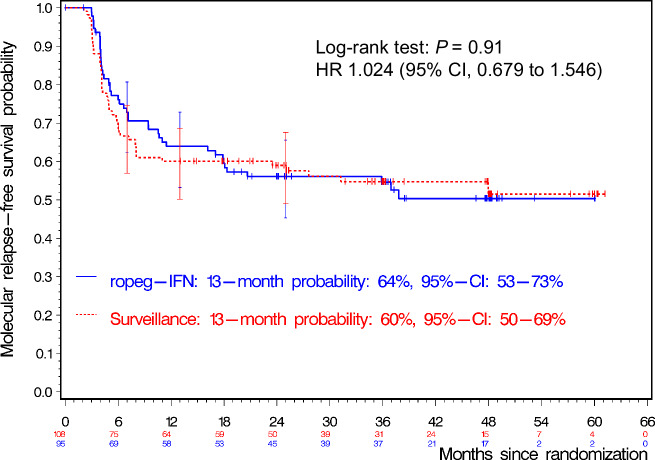
Kaplan-Meier analysis of molecular relapse–free survival (MRFS) in the ENDURE trial. MRFS probabilities are shown for patients with CML who discontinued TKI therapy after one month and were randomized to receive either ropeginterferon alfa-2b (ropeg-IFN) maintenance for 15 months or surveillance. At months 7, 13, and 25 after randomization (corresponding to 6, 12, and 24 months after TKI discontinuation), MRFS probabilities were 73%, 64%, and 56% in the ropeg-IFN arm and 67%, 60%, and 59% in the surveillance arm, respectively. Numbers below the graph indicate patients at risk at each time point. CI confidence interval, HR hazard ratio.

The Kaplan-Meier probabilities MRFS by 6, 12, and 24 months after TKI discontinuation were 73% (95%-CI, 62–81%), 64% (53–73%) and 56% (45–66%) for the ropeg-IFN versus 67% (57–75%), 60% (50–69%) and 59% (49–68%) for no treatment (Fig. 2). The hazard ratio (HR) of molecular relapse for the no treatment cohort versus the ropeg-IFN cohort was 1.024 (95% CI, 0.679–1.546; log-rank *P* = 0.91). The result of the log-rank test stratified for a prior TKI stopping attempt was *P* = 0.96.

The probability of MRFS at 6, 12, and 24 months after TKI discontinuation (secondary endpoints) were 70% (95%-CI, 60–79%), 64% (95%-CI, 53–73%) and 49% (95%-CI, 38–60%) in 91, 91, and 78 patients of the ropeg-IFN group versus 65% (95-CI, 56–74%; *p* = 0.23, one-sided), 59% (95-CI, 50–68%, *p* = 0.27) and 50% (95-CI, 40–60%; *p* = 0.93) in 107, 106 and 92 patients of the surveillance group. A post hoc subgroup analysis of patients with a first discontinuation attempt (*n* = 108) and a treatment duration of more than 6 years favored TFR with ropeg-IFN, but the difference between the two arms were not statistically significant (Fig. S1).

### Safety

**Fig. 3:**
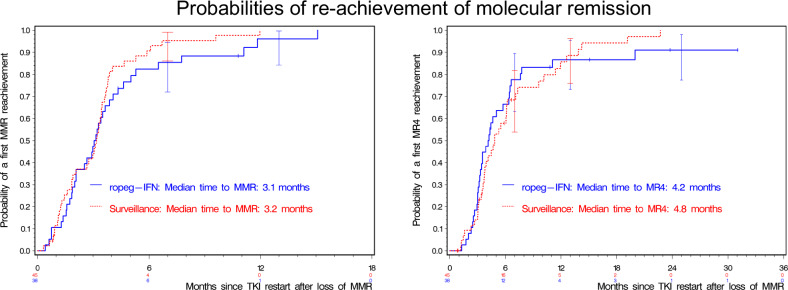
Probabillities of reachievement of molecular remission after loss of MMR. Left panel: Median time to reachievement of MMR was 3.1 months in the ropeg-IFN group vs. 3.2 months in the placebo group. Right panel: Median time to MR [4] was 4.2 months in the ropeg-IFN group vs. 4.8. months in the placebo group. MMR, major molecular remission; MR [4], molecular remission BCR::ABL ≤ 0.01% according to international scale; TKI tyrosine kinase inhibitor.

Ninety patients lost MMR after TKI stop and were candidates for restarting TKI. Molecular data on 83 patients were available after TKI restart. Of those, 79 patients re-achieved at least MMR, 78 within 12 months. The median time to re-achievement of MMR was 3 months (Fig. 3, IQR: 2-4 months). Of the 4 patients who did not regain MMR, one patient withdrew consent and others were observed for only 1, 4, and 11 months after treatment restart. Of the evaluable 83 patients, 72 patients re-achieved a first MR [4] after a median time of 4.4 months (Fig. 3). Of 11 patients who did not, 7 patients had a follow-up after TKI restart of less than 7 months and had no MR [4] within this time. The 4 other patients without MR [4] re-achievement had their last evaluations at 11, 15, 24, and 31 months after restart.

**Fig. 4:**
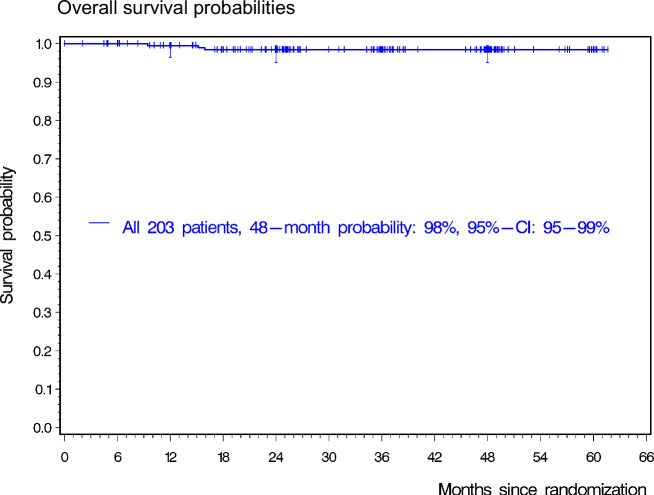
Overall survival probability. There were three deaths in the trial. One patient died due to a cardiac arrest while in MMR. The cause of death was unknown for the second patient. A third patient died after falling from stairs. The latter two patients had regained deep molecular response at the level MR4 after re-commencing TKI therapy for MMR-loss. At 48 months, 53 patients were still under observation. CI confidence interval.

After a median observation time of 36 months (IQR: 25–48 months), none of the patients progressed and three patients died after 9, 15, and 16 months (Fig. 4). One patient died due to a cardiac arrest while in MMR. The cause of death was unknown for the second patient. A third patient died after falling from stairs. There were no CML-specific deaths in the trial.

### Ropeg-IFN dosing, safety and toxicity

Patients randomized to the ropeg-IFN arm were scheduled for a maximum of 32 biweekly administrations during the fifteen months of maintenance treatment. Ninety four of 95 patients who were randomized to ropeg-IFN received at least one ropeg-IFN dose. Of those, 58 patients had a ropeg-IFN treatment observation time of at least 15 months and maintained MMR (MMR cohort). 41 patients in the MMR cohort (71%) actually received at least 30 biweekly ropeg-IFN injections, albeit at a reduced dose in four patients. The remaining 17 patients in the MMR cohort (29%) received less than at least 30 ropeg-IFN injections due to AE (*n* = 4) or ropeg-IFN-discontinuation for other reasons (*n* = 13). Of the other 36 ropeg-IFN patients who were not observed for at least 15 months without loss of MMR, 32 patients discontinued ropeg-IFN due to loss of MMR, 1 patient due to an AE and 3 patients because of other reasons. The median ropeg-IFN dose in the 94 patients receiving at least one ropeg-IFN dose was 92 µg (range, 3–104 µg).

174 of 202 patients (86,1%) of the safety population experienced at least one AE. There were 87 patients with any AE in the ropeg-IFN arm (92,6%) and 87 patients with any AE in the surveillance arm (80,6%). Lower grade AEs were frequent in both arms (Supplemental Table 1). Thirty-two higher grade AEs (WHO° 3 or 4) were observed in the ropeg-IFN arm and 26 higher grade AEs (only WHO° 3) in the surveillance group (Table 2). Of the 29 reported SAEs in 21 patients with moderate to severe intensity, 7 SAEs in six patients were evaluated as possibly related to ropeg-IFN (*n* = 5) or surveillance (*n* = 2) (Supplemental Table 2). Together, ropeg-IFN maintenance treatment was well tolerated in CML patients in TFR with no new safety signals for ropeg-IFN.

**Table 2:** Incidence of high-grade adverse events (safety population).

Characteristics	All patients (*n* = 203)	Surveillance (*n* = 108)	Ropeg-IFN (*n* = 95)
**Age at diagnosis (years)**			
median	46	47	45
range	14–83	14–83	19–74
**Age at randomization (years; median, range)**	55, 20–88	56, 20–88	53, 24–78
**Sex – no. (%)**			
Female	68 (33)	42 (39)	26 (27)
Male	135 (67)	66 (61)	69 (73)
**Median time on TKI at baseline (years; median, range)**	7,8 (2,5–19,7)	7,9 (2,5–19,7)	7,7 (3–19,1)
**TKI at baseline – no (%)**			
imatinib	86 (42)	44 (41)	42 (44)
nilotinib	64 (32)	35 (32)	29 (31)
dasatinib	47 (23)	26 (24)	21 (22)
bosutinib	4 (2)	2 (2)	2 (2)
ponatinib	1 (1)	1 (1)	1 (1)
**prior TKI stops – no (%)**			
none	163 (80)	86 (80)	77 (81)
1 or more	40 (20)	22 (20)	18 (19)
**Prognostic high risk at diagnosis**			
ELTS - no	128		
high risk – no (%)	19/15	13/19	6/10
EUTOS - no	132		
high risk – no (%)	18/14	11/15	7/12
SOKAL	128		
high risk – no (%)	30/23	17/25	13/22
EURO - no	125		
high risk – no (%)	16/13	7/10	9/16

All data are presented as No. (%) unless otherwise indicated.

*COVID* coronavirus disease, *PNP* peripheral neuropathic pain, *ropeg-IFN* ropeginterferon alfa-2b.

Safety population (patients, who received at least one time study medication).

## Discussion

In the randomized ENDURE trial, IFN maintenance conferred no additional benefit in sustaining TFR among CML patients who had achieved a deep molecular remission with TKI monotherapy. While this finding is consistent with results from the large randomized German CML-V (TIGER) trial — which likewise failed to demonstrate a significant TFR benefit from IFN maintenance, albeit following first line nilotinib plus IFN induction therapy [20]—it clearly contrasts with a large body of indirect evidence that had implied a potential clinical value for IFN in improving TFR outcomes in the TKI era.

This discrepancy is noteworthy, given that IFN monotherapy induces complete cytogenetic responses in approximately 20% of CML patients [42–45], a considerable proportion of whom may achieve durable treatment-free remission after IFN discontinuation [6]. In addition, IFN has been shown to intensifying molecular responses when combined with TKI therapy [14–17], which is clinically relevant because early and deep molecular remission is a well-established prerequisite for achieving successful TFR [23]. Furthermore, in vivo studies have demonstrated IFN-induced expansion of CML-specific cytotoxic T and NK cells [10–13, 19, 35, 46–48]. Collectively, these findings supported the hypothesis that IFN might augment durable disease control in TKI-pretreated patients by enhancing immunological effector mechanisms following TKI cessation.

However, the ENDURE study results do not support this assumption. IFN maintenance failed to improve the probability of sustained TFR in patients who had received standard TKI monotherapy, demonstrating that, in unselected patients, IFN provides no additional benefit beyond what is already achieved through long-term TKI therapy [37, 48]. Poor tolerability is an unlikely explanation for this outcome, as dose reductions were infrequent and severe adverse events rare. In fact, owing to its novel biochemical and pharmacological characteristics, ropeg-IFN has a markedly favorable toxicity profile compared to conventional IFN formulations or pegylated variants [16, 38].

Although the ENDURE trial failed to confirm a benefit in its primary endpoint, it was crucial in objectively testing and contextualizing previous non-randomized or translational evidence that had suggested an interferon-related improvement in TFR rates. In this sense, the ENDURE trial may be regarded as concluding the long-standing exploration of IFN-based strategies in CML, pending a clearer mechanistic distinction between IFN- and TKI-associated pathways to TFR.

## Supplementary information

Study protocol

Study protocol - Amendment

Supplemental Figure 1

Supplemental Tables

## Data Availability

The datasets used during the current study are available from the corresponding author on reasonable request.
