# Supplementary material for: Randomized phase 3 trial of Ropeginterferon alfa-2b versus surveillance after tyrosine kinase inhibitor discontinuation in chronic myeloid leukemia (ENDURE/CML-IX)
Source: Leukemia. 2026 Jan 12;40(2):410–7. doi: 10.1038/s41375-025-02859-1 (PMC12875868; doi:10.1038/s41375-025-02859-1)
Supplement: Supplementary file 1 — Study protocol [file 41375_2025_2859_MOESM1_ESM.pdf]

Clinical Study Protocol

EFFICACY AND SAFETY OF PEGYLATED-PROLINE- INTERFERON ALPHA 2B (AOP2014) IN MAINTAINING DEEP MOLECULAR REMISSIONS IN PATIENTS WITH CHRONIC MYELOID LEUKEMIA (CML) WHO DISCONTINUE ABL-KINASE INHIBITOR THERAPY - A RANDOMIZED PHASE II, MULTICENTER TRIAL WITH POST-STUDY FOLLOW-UP

Authors: A. Burchert, A. Hochhaus, S. Saussele, K. Piskulak, M. Pfirrmann, C. Schade-Brittinger

**Short Title:**	**ENDURE-CML (CML-IX study)**
**Protocol Code**	KKS-227
**EudraCT Number:**	2016-001030-94
**Clinical Trial Register Number:**	NCT03117816
**Investigational Product:**	Pegylated-Proline-interferon alpha-2b (AOP2014)
**Sponsor:**	Philipps University Marburg<br>Biegenstr. 10<br>D-35037 Marburg
**Coordinating Investigator:**	Prof. Dr. Andreas Burchert<br>Department of Hematology/Oncology/Immunology<br>University Hospital Marburg<br>Baldingerstrasse<br>D-35043 Marburg
**Date:**	04.12.2017
**Version:**	V04 F

This document and all its including parts are property of Prof. Dr. A. Burchert, Prof. Dr. A. Hochhaus, PD Dr. Susanne Saussele on behalf of the CML study group / CML study alliance and KKS Marburg. It is at confidential disposal to the members of the study team in the participating centers. The use of such confidential information must be restricted to the recipient for the agreed purpose and must not be disclosed, published or otherwise communicated to any unauthorized person, for any reason, in any form whatsoever without prior written approval of the parties stated above.

Protocol Approval Signatures

I have read this protocol and agree to conduct the study in accordance with all commitments of this protocol as well as with the current version of the Declaration of Helsinki, ICH-GCP E6 Guideline (International Conference on Harmonization - Good Clinical Practice) and applicable national laws and regulatory requirements. I also agree to handle all information concerning this study confidentially.

Representative of the Sponsor

Carmen Schade-Brittinger
KKS Marburg
Karl-von-Frisch-Straße 4
D-35043 Marburg

C. Schade-Brittinger
Signature

06.12.17
Date

Coordinating Investigator

Prof. Dr. med. Andreas Burchert
Department of
Hematology/Oncology/Immunology
University Hospital Marburg
Baldingerstr. 1
D-35043 Marburg

Signature

04.12.2017
Date

Biometrician

PD. Dr. rer. biol. hum. Markus Pfirrmann
Institut für Medizinische
Informationsverarbeitung, Biometrie und
Epidemiologie (IBE)
Marchioninistr. 15
D-81377 München

Signature

Date

German CML Study Group

Prof. Dr. med. Andreas Hochhaus
Universitätsklinikum Jena
Klinik für Innere Medizin II
Abteilung Hämatologie und Internistische
Onkologie
Erlanger Allee 101
D-07740 Jena

Signature

Date

PD Dr. med. Susanne Saußele
(Subprojekt QoL)
Medizinische Fakultät Mannheim der
Universität Heidelberg
III. Medizinische Klinik
Pettenkoferstrasse 22
D-68169 Mannheim

Signature

Date

Protocol Approval Signatures

I have read this protocol and agree to conduct the study in accordance with all commitments of this protocol as well as with the current version of the Declaration of Helsinki, ICH-GCP E6 Guideline (International Conference on Harmonization - Good Clinical Practice) and applicable national laws and regulatory requirements. I also agree to handle all information concerning this study confidentially.

Representative of the Sponsor

Carmen Schade-Brittinger
KKS Marburg
Karl-von-Frisch-Straße 4
D-35043 Marburg

Signature

Date

Coordinating Investigator

Prof. Dr. med. Andreas Burchert
Department of
Hematology/Oncology/Immunology
University Hospital Marburg
Baldingerstr. 1
D-35043 Marburg

Signature

Date

Biometrician

PD. Dr. rer. biol. hum. Markus Pfirrmann
Institut für Medizinische
Informationsverarbeitung, Biometrie und
Epidemiologie (IBE)
Marchioninistr. 15
D-81377 München

Signature

05/12/17
Date

German CML Study Group

Prof. Dr. med. Andreas Hochhaus
Universitätsklinikum Jena
Klinik für Innere Medizin II
Abteilung Hämatologie und Internistische
Onkologie
Erlanger Allee 101
D-07740 Jena

Signature

Date

PD Dr. med. Susanne Saußele
(Subprojekt QoL)
Medizinische Fakultät Mannheim der
Universität Heidelberg
III. Medizinische Klinik
Pettenkoferstrasse 22
D-68169 Mannheim

Signature

Date

Protocol Approval Signatures

I have read this protocol and agree to conduct the study in accordance with all commitments of this protocol as well as with the current version of the Declaration of Helsinki, ICH-GCP E6 Guideline (International Conference on Harmonization - Good Clinical Practice) and applicable national laws and regulatory requirements. I also agree to handle all information concerning this study confidentially.

**Representative of the Sponsor**<br>Carmen Schade-Brittinger<br>KKS Marburg<br>Karl-von-Frisch-Straße 4<br>D-35043 Marburg	Signature<br><br>Date
**Coordinating Investigator**<br>Prof. Dr. med. Andreas Burchert<br>Department of Hematology/Oncology/Immunology<br>University Hospital Marburg<br>Baldingerstr. 1<br>D-35043 Marburg	Signature<br><br>Date
**Biometrician**<br>PD. Dr. rer. biol. hum. Markus Pfirrmann<br>Institut für Medizinische Informationsverarbeitung, Biometrie und Epidemiologie (IBE)<br>Marchioninistr. 15<br>D-81377 München	Signature<br><br>Date
**German CML Study Group**<br>Prof. Dr. med. Andreas Hochhaus<br>Universitätsklinikum Jena<br>Klinik für Innere Medizin II<br>Abteilung Hämatologie und Internistische Onkologie<br>Erlanger Allee 101<br>D-07740 Jena	[signature]<br>Signature<br>4 12 17<br>Date<br>UNIVERSITÄTSKLINIKUM JENA Klinik für Innere [illegible] Onkologie [illegible]
PD Dr. med. Susanne Saußele<br>(Subprojekt QoL)<br>Medizinische Fakultät Mannheim der Universität Heidelberg<br>III. Medizinische Klinik<br>Pettenkoferstrasse 22<br>D-68169 Mannheim	Signature<br><br>Date

Protocol Approval Signatures

I have read this protocol and agree to conduct the study in accordance with all commitments of this protocol as well as with the current version of the Declaration of Helsinki, ICH-GCP E6 Guideline (International Conference on Harmonization - Good Clinical Practice) and applicable national laws and regulatory requirements. I also agree to handle all information concerning this study confidentially.

Representative of the Sponsor

Carmen Schade-Brittinger
KKS Marburg
Karl-von-Frisch-Straße 4
D-35043 Marburg

Signature

Date

Coordinating Investigator

Prof. Dr. med. Andreas Burchert
Department of
Hematology/Oncology/Immunology
University Hospital Marburg
Baldingerstr. 1
D-35043 Marburg

Signature

Date

Biometrician

PD. Dr. rer. biol. hum. Markus Pfirrmann
Institut für Medizinische
Informationsverarbeitung, Biometrie und
Epidemiologie (IBE)
Marchioninistr. 15
D-81377 München

Signature

Date

German CML Study Group

Prof. Dr. med. Andreas Hochhaus
Universitätsklinikum Jena
Klinik für Innere Medizin II
Abteilung Hämatologie und Internistische
Onkologie
Erlanger Allee 101
D-07740 Jena

Signature

Date

PD Dr. med. Susanne Saußele
(Subprojekt QoL)
Medizinische Fakultät Mannheim der
Universität Heidelberg
III. Medizinische Klinik
Pettenkoferstrasse 22
D-68169 Mannheim

Signature

5.12.17
Date

Consent Statement to Study Protocol

Principal Investigator (PI) and Deputy PI at site

I have read this protocol and agree to conduct the study in accordance with the study protocol, the current version of the Declaration of Helsinki, ICH-GCP E6 Guideline (International Conference on Harmonization - Good Clinical Practice) and, applicable national laws and regulatory requirements. I also agree to handle all information concerning this study confidentially.

I will ensure that all personnel involved in the study under my direction will be informed about the contents of this study protocol and will receive all necessary instructions for performing the study according to the study protocol.

Principal Investigator at the site

____

Name in printed letters

________________ ______________________________

Date Signature

Deputy Principal Investigator at the site

____

Name in printed letters

________________ ______________________________

Date Signature

Consent Statement to Study Protocol

Subinvestigators at site

I have read this protocol and agree to conduct the study in accordance with the study protocol, the current version of the Declaration of Helsinki, ICH-GCP E6 Guideline (International Conference on Harmonization - Good Clinical Practice) and, applicable national laws and regulatory requirements. I also agree to handle all information concerning this study confidentially.

Subinvestigator

Name in printed letters

Date Signature

Subinvestigator

Name in printed letters

Date Signature

Subinvestigator

Name in printed letters

Date Signature

Subinvestigator

Name in printed letters

Date Signature

Study Personnel

**Coordinating Investigator**<br>**(LKP according to §40 AMG)**<br>**and**<br>**Contact for medical questions**	**Prof. Dr. med. Andreas Burchert**<br>Klinik für Hämatologie, Onkologie und Immunologie<br>Universitätsklinikum Gießen und Marburg GmbH, Standort Marburg<br>Baldingerstraße<br>35043 Marburg<br>Phone: 06421 586 6511/ -6512<br>Fax: 06421 586 5062<br>E-mail: burchert@staff.uni-marburg.de
**Sponsor**	**Philipps-University Marburg**<br>Biegenstraße 10<br>35037 Marburg<br>**Representative of the Sponsor**<br>Koordinierungszentrum für Klinische Studien (KKS)<br>Philipps-Universität Marburg<br>Karl-von-Frisch-Straße 4<br>35043 Marburg
**Statistician**	**PD Dr. rer. biol. hum. Markus Pfirrmann**<br>Institut für Medizinische Informationsverarbeitung, Biometrie und Epidemiologie (IBE)<br>Marchioninistraße 15<br>81377 München<br>Phone: 089 4400-77489<br>Fax: 089 4400-77491<br>E-Mail: pfi@ibe.med.uni-muenchen.de
**Project Management**	**Dr. Karin Weide und**<br>**Kerstin Balthasar**<br>KKS Marburg<br>Karl-von-Frisch-Straße 4<br>35043 Marburg<br>Phone: 06421-28-66785 / 28 66558<br>Fax: 06421-28 66517
**Monitoring**	**Schantl Pharma Service GmbH**<br>Herderstraße 16<br>65185 Wiesbaden
**SAE-Management /**<br>**Contact for SAE reporting**	**KKS Marburg**<br>Safety Division<br>Karl-von-Frisch-Straße 4<br>35043 Marburg<br>Phone: 06421-28 66510 or -28 66554<br>Fax: 06421-28 66559<br>E-Mail: sae-management@kks.uni-marburg.de
**Data Management**	**KKS Marburg**<br>Data Management Division<br>Karl-von-Frisch-Straße 4<br>35043 Marburg<br>Fax: 06421-28 66516

**FAX-Randomization at KKS Marburg**	**KKS Marburg**<br>Data Management<br>Fax: 06421-28 66516
**Data Safety Monitoring Committee (DSMC)**	**Prof. Dr. A. Reiter, Hämatologie, Uni Mannheim**<br>**Prof. Dr. M. Stelljes, Hämatologie, Uni Münster**<br>**Heinz Haverkamp, Universitätsklinikum Köln**
**Steering Committee**	**Prof. Dr. med. Andreas Burchert**<br>(contact details please see “Coordinating Investigator”)<br><br>**Prof. Dr. med. Andreas Hochhaus**<br>Dep. Internal Medicine II, Hematology/Oncology<br>University Hospital Jena<br>Erlanger Allee 101<br>07740 Jena<br>Phone: 03641 9-324200<br>Fax: 03641 9-324202<br><br>**Prof. Dr. med. Susanne Saussele**<br>III. Internal Medicine Department<br>University Hospital Mannheim<br>University Heidelberg<br>Pettenkoferstr. 23<br>68169 Mannheim<br>Phone: 0621-383-6966<br>Fax: 0621-383-6968<br><br>**PD Dr. Markus Pfirrmann**<br>Institut für Medizinische Informationsverarbeitung, Biometrie und Epidemiologie (IBE)<br>(contact details please see „Statistician“)<br><br>**Carmen Schade-Brittinger**<br>Koordinierungszentrum für klinische Studien (KKS)<br>Karl-von-Frisch-Straße 4<br>35043 Marburg<br>Phone: 06421-286-6458<br>Fax: 06421-286-6517
**Manufacturer of study medication**	**AOP Orphan Pharmaceuticals AG**<br>Wilhelminenstraße 91/II f<br>A-1160 Wien<br>Phone: +43 1 503 72 44-0

**Translational Laboratory**	
• ***Reference Molecular Laboratory BCR-ABL mRNA***	**Prof. Dr. med. Andreas Hochhaus**<br>Universitätsklinikum Jena<br>Medizinische Universitäts-Laboratorien<br>Hämatologisch-onkologisches Speziallabor<br>Postfach 100236<br>07702 Jena<br>Phone: 03641 9-324266<br><br>**Prof. Dr. med. Susanne Saussele**<br>Wissenschaftliches Labor<br>III. Medizinische Klinik<br>Universitätsmedizin Mannheim<br>Pettenkoferstr. 22<br>68169 Mannheim
• ***Immune marker & stem cell*** **laboratory**	**AG Prof. Dr. med. Andreas Burchert**<br>Laboratory for Molecular and translational leukemia research<br>Philipps University Marburg<br>Hans Meerwein Straße 3<br>35043 Marburg
• ***Interferon Response***	**Prof. Dr. med. Steffen Koschmieder / Frau Kristina Feldberg**<br>Hämatologisches Labor<br>Klinik für Hämatologie, Onkologie, Hämostaseologie und Stammzelltransplantation<br>RWTH Uniklinik Aachen<br>Pauwelsstr. 30<br>52078 Aachen<br>Phone: 0241-8036102

Concept, design and strategic recruitment plan of the *ENDURE* – CML study was discussed and approved by the CML-study group steering committee / CML study alliance at the CML-study meeting in Weimar (17./18.04.2015)

Prof. Dr. G. Baerlocher, Bern (SAKK)	Prof. Dr. R. Hehlmann, Mannheim
Prof. Dr. T. Brümmendorf, Aachen	Prof. Dr. S. Krause, Erlangen
Prof. Dr. G. Ehninger, Dresden	Prof. Dr. P. Le Coutre, Berlin
Prof. Dr. J. Hasford, München	Prof. Dr. A. Neubauer, Marburg
Prof. Dr. D. Heim, Basel (SAKK)	Prof. Dr. D. Niederwieser, Leipzig
Prof. Dr. A. Hochhaus, Jena	Prof. Dr. A. Burchert, Marburg
PD Dr. M. Pfirrmann, IBE München	

.

Table of Contents

PROTOCOL APPROVAL SIGNATURES **2**

STUDY PERSONNEL **5**

TABLE OF CONTENTS **8**

LIST OF ABBREVIATIONS AND DEFINITION OF TERMS **12**

1 SYNOPSIS **14**

1.1 STUDY FLOW CHART ENDURE-CML 20
1.2 ENDURE-CML: STUDY SPECIFIC PROCEDURES FOR ARM A (AOP2014) 21
1.3 ENDURE-CML: STUDY SPECIFIC PROCEDURES FOR ARM B (SURVEILLANCE) 22

2 INTRODUCTION **24**

2.1 BACKGROUND 24
2.1.1 Chronic myeloid leukemia 24
2.1.2 CML treatment with tyrosine kinase inhibitors (TKI) 24
2.1.3 Molecular response under TKI therapy 24
2.1.4 TKI discontinuation in CML in deep molecular remission 24
2.2 INTERFERON ALPHA TO IMPROVE THE RATE OF DURABLE TKI DISCONTINUATION 25
2.3 PEGYLATED-PROLINE-INTERFERON ALPHA-2B (AOP2014) 26
2.3.1 Pharmacokinetics 26
2.3.2 Clinical experience with AOP2014 27
2.4 RATIONALE FOR THE STUDY 28
2.5 RISK BENEFIT ASSESSMENT 28

3 STUDY OBJECTIVES **29**

3.1 PRIMARY OBJECTIVE 29
3.2 SECONDARY OBJECTIVES 29
3.3 POST STUDY FOLLOW UP (PSFU) OBJECTIVES 29

4 STUDY ENDPOINTS **30**

4.1 PRIMARY ENDPOINT 30
4.2 SECONDARY ENDPOINTS 30

5 POST STUDY FOLLOW UP ENDPOINTS **30**

5.1 SECONDARY ENDPOINTS 30

6 RANDOMIZATION **31**

7 BLINDING **31**

8 INVESTIGATIONAL PLAN **32**

8.1 OVERALL STUDY DESIGN 32
8.2 DISCUSSION OF STUDY DESIGN 32
8.2.1 TKI discontinuation 32
8.2.2 Initial combined TKI and AOP2014 treatment in arm A 32
8.2.3 AOP2014 as maintenance therapy in molecular remission 32
8.2.4 AOP2014 treatment duration 32
8.2.5 Post Study Follow Up (PSFU) 33
8.2.6 Expected treatment improvement by AOP2014 33

9 STUDY POPULATION **33**

9.1 DEFINITION OF DEEP MOLECULAR REMISSION 33
9.2 INCLUSION CRITERIA 33
9.3 EXCLUSION CRITERIA 34
9.4 FEASIBILITY OF RECRUITMENT 35

10 STUDY MEDICATION (IMP)....... 35

10.1 CHARACTERISTICS OF INVESTIGATIONAL MEDICINAL PRODUCT (IMP)....... 35
10.2 POTENTIAL TOXICITY IN PATIENTS....... 35
10.3 SELECTION OF DOSE IN THE STUDY....... 38
10.3.1.1 Dose reductions and modifications....... 38
10.4 MANUFACTURER OF STUDY MEDICATION....... 40
10.5 HANDLING AND STORAGE OF THE MEDICATION....... 40
10.6 REFERENCE DOCUMENT....... 40
10.7 LABELING, DRUG SUPPLY AND ACCOUNTING....... 40
10.8 PRIOR AND CONCOMITANT THERAPY....... 41

11 STUDY MEDICATION (NON-IMP)....... 41

11.1 CHARACTERISTICS OF NON-INVESTIGATIONAL MEDICINAL PRODUCT (NON-IMP)....... 41
11.2 SIDE EFFECTS CAUSED BY DISCONTINUATION OF TKI THERAPY:....... 41

12 STUDY PROCEDURES AND METHODS....... 42

12.1 STUDY PROCEDURES....... 42
12.1.1 Screening Visit and Registration....... 42
12.1.2 Randomization and Baseline Visit (V1)....... 42
12.1.2.1 Randomization....... 42
12.1.2.2 Baseline Visit (V1) - Month 1....... 42
12.1.3 Visit 2 – 12 (Month 2 – Month 13.5)....... 43
12.1.4 End-of-Treatment assessment Visit 13 (Month 15)....... 44
12.1.5 Premature End-of-Treatment assessments....... 44
12.1.6 Surveillance phase (month 15 to month 25)....... 44
12.1.7 Post Study Follow Up (PSFU)....... 44
12.1.8 Study Schedule....... 45
12.2 STUDY METHODS....... 45
12.2.1 Demography....... 45
12.2.2 EUTOS Score....... 45
12.2.3 Euro and Sokal Scores....... 45
12.2.4 ELTS score....... 45
12.2.5 Medical and pre-treatment history....... 45
12.2.6 Physical Examination / Vital Signs / Body Temperature....... 45
12.2.7 ECOG Performance Status....... 45
12.2.8 Clinical Laboratory Evaluation....... 46
12.2.8.1 Hematology....... 46
12.2.8.2 Coagulation....... 46
12.2.8.3 Clinical Chemistry....... 46
12.2.9 Autoimmunity....... 46
12.2.10 BCR-ABL measurement and genetic studies....... 47
12.2.11 Other Translational studies....... 47
12.2.12 Total Blood Volume to be collected from Each Patient....... 48
12.2.13 Pregnancy test....... 48
12.2.14 Local Tolerability....... 48
12.2.15 Concomitant treatment....... 48
12.2.16 Quality of Life Questionnaires....... 49
12.2.17 Patient Diary....... 49

13 DISCONTINUATION CRITERIA....... 49

13.1 WITHDRAWAL OF PATIENTS FROM STUDY TREATMENT....... 49
13.2 PREMATURE DISCONTINUATION OF THE STUDY....... 50
13.2.1 Single center....... 50
13.2.2 Study as a whole....... 50

14 SAFETY....... 50

14.1 DEFINITION....... 50
14.1.1 Adverse Events....... 50

14.1.2 *Adverse Reaction (AR)* 50
14.1.3 *Unexpected Adverse Reaction (UAR)* 51
14.1.4 *Serious Adverse Event (SAE) or Serious Adverse Reaction (SAR)* 51
14.1.5 *Suspected Unexpected Serious Adverse Reaction (SUSAR)* 51
14.2 Documentation of Adverse Events 52
14.3 Severity 52
14.4 Causality 52
14.4.1 *Seriousness* 53
14.4.2 *Outcome* 53
14.5 Documentation and reporting of Serious Adverse Events 53
14.6 Exceptions from SAE-reporting 54
14.7 Expectedness for IMP (Arm A) 54
14.8 Expectedness for Non-IMP 54
14.9 Clinical assessment of relatedness for Non-IMP (both Arms) 54
14.10 Pregnancy 55
14.11 Adverse events of special interest 55
14.12 SUSAR reporting procedure 55
14.13 Safety-Manual 55

15 DATA MANAGEMENT 56

15.1 EDC-System (e-CRF) and Data Management 56

16 STATISTICAL CONSIDERATIONS 57

16.1 Hypotheses 57
16.2 Analysis Populations 58
16.3 Demographic and Other Baseline Characteristics 58
16.4 Efficacy Variables 58
16.5 Quality of life assessment 58
16.6 Safety Variables 59
16.7 Methods of Analysis 59
16.8 Interim Analyses 60
16.9 Analyses of "off study follow-up" 61
16.10 Sample Size Calculation 61
16.11 General principles of statistical analyses 62

17 ADMINISTRATION 62

17.1 Source Data and Patient Files 62
17.2 Data Safety and Monitoring Committee (DSMC) 63
17.3 Monitoring, Audit and Inspection 63
17.3.1 *Monitoring* 63
17.3.2 *Audit and Inspections* 63
17.4 Investigator Site File (ISF) and archiving 63
17.5 Protocol violation and discrepancies 64

18 ETHICAL CONSIDERATIONS 64

19 ETHICAL AND REGULATORY ASPECTS 64

19.1 Investigator's responsibilities 64
19.2 Further responsibilities 64
19.3 Patient information and consent 64
19.4 Patient insurance 65
19.5 Ethics Committee (EC) or Institutional Review Board 65
19.6 Regulatory Authorities 66
19.6.1 *Notification to competent authority* 66
19.6.2 *Notification to the local competent authority* 66
19.7 Changes to Study Protocol 66
19.8 Safety of subjects, immediate danger 67
19.9 Pre-conditions before study start 67

20 FINANCIAL ASPECTS 67

21 FINAL REPORT 67

22 REGISTRATION 68

23 PUBLICATION 68

24 REFERENCES 69

TABLE 1 STUDY SPECIFIC PROCEDURES FOR ARM A 21
TABLE 2 STUDY SPECIFIC PROCEDURES FOR ARM B 22
TABLE 3 RECOMMENDED AOP2014 DOSAGE MODIFICATIONS FOR NEUTROPENIA AND THROMBOCYTOPENIA 38
TABLE 4 RECOMMENDED DOSAGE MODIFICATION FOR DEPRESSION MANAGEMENT 39
TABLE 6 RECOMMENDED DOSE MODIFICATIONS IN CASE OF OTHER NON-HEMATOLOGICAL TOXICITIES 39
TABLE 7 ECOG PERFORMANCE STATUS CRITERIA 46
TABLE 8 HEMATOLOGY ALERT VALUES 46
TABLE 9 TYPE AND VOLUME OF BLOOD SAMPLING 48
TABLE 10 CAUSALITY ASSESSMENT CRITERIA 53
TABLE 11 DEFINITIONS OF ANALYSIS POPULATIONS 58

GERMAN CML.STUDY GROUP

List of Abbreviations and Definition of Terms

ADL	=	activities of daily living
AE	=	adverse event
ALT	=	alanine aminotransferase
ANA	=	antinuclear antibodies
ANCOVA	=	analysis of covariance
AMG	=	Arzneimittelgesetz
AP	=	alkaline phosphatase
AOP2014	=	Pegylated-Proline-interferon alpha-2b
AST	=	aspartate aminotransferase
BCR-ABL	=	Breakpoint cluster region-Abelson Onkogen
BfArM	=	Bundesinstitut für Arzneimittelmedizinprodukte
CA	=	competent authority
CML	=	chronic myeloid leukemia
CHO	=	chinese hamster ovary
CSR	=	clinical study report
CTCAE	=	Common Terminology Criteria for Adverse Events
DNA	=	deoxyribonucleic acid
DPBS	=	Dulbecco's phosphate-buffered saline
DSMC	=	data safety monitoring committee
e-CRF	=	electronic case report form
ECOG	=	Eastern Cooperative Oncology Group
EDTA	=	ethylene diaminetetraacetic acid
ELISA	=	enzyme-linked immunosorbent assay
FACS	=	fluorescence activated cell sorting
FDA	=	Food and Drug Administration
GCP	=	Good Clinical Practice
GGT	=	gamma glutamyltransferase
hERG	=	human ether-à-go-go-related gene
HIV	=	human immunodeficiency virus
HR	=	hazard ratio
ICH-GCP	=	International Conference on Harmonization - Good Clinical Practice
IB	=	Investigator´s Brochure
IMP	=	Investigational Medicinal Product
ITT	=	Intention to treat
ICF	=	informed consent form
ICH	=	International Conference on Harmonisation
IEC	=	independent ethics committee
IFN	=	interferon alpha
IS	=	international scale
ISF	=	Investigator Site File
irRC	=	immune-related response criteria
IWRS	=	interactive web response system
LDH	=	lactate dehydrogenase
MedDRA	=	Medical Dictionary for Regulatory Activities
MMR	=	major molecular response
MR^4	=	deep molecular remission
NCI	=	National Cancer Institute
OC RDC	=	Oracle Clinical Remote Data Capture
OS	=	overall survival
PBMC	=	peripheral blood mononuclear cells
pDC	=	plasmacytoid dendritic cells
pegIFN	=	pegylated interferon
PFS	=	progression free survival: no increase in BCR-ABL ratio to >0.1% (IS)

PS	=	performance score
PCR	=	Polymerase chain reaction
PI	=	Principal Investigator
SAE	=	serious adverse event
sc	=	subcutaneous
SAR	=	Serious Adverse Reaction
SDV	=	Source Data Verification
SmPC	=	summary of product characteristics
SPD	=	sum of the products of the 2 largest perpendicular diameters
SUSAR	=	suspected unexpected serious adverse reaction
TKI	=	tyrosine kinase inhibitor
TFR	=	treatment free remission
TEAE	=	treatment-emergent adverse event
ULN	=	upper limit of normal
vs	=	versus
WHO	=	World Health Organisation

1 Synopsis

**Title:**	EFFICACY AND SAFETY OF PEGYLATED-PROLINE-INTERFERON ALPHA 2B IN MAINTAINING DEEP MOLECULAR REMISSIONS IN PATIENTS WITH CHRONIC MYELOID LEUKEMIA (CML) WHO DISCONTINUE ABL-KINASE INHIBITOR THERAPY - A RANDOMIZED PHASE II, MULTICENTER TRIAL WITH POST-STUDY FOLLOW-UP
**Acronym**	ENDURE-CML (CML-IX study)
**Sponsor**	Philipps-University Marburg
**Representative the Sponsor**	Koordinierungszentrum für Klinische Studien (KKS) Marburg<br>Karl-von-Frisch-Straße 4<br>35043 Marburg
**Coordinating Investigator (LKP according to § 40 AMG)**	Prof. Dr. med. Andreas Burchert<br>Klinik für Hämatologie, Onkologie und Immunologie<br>Universitätsklinikum Gießen und Marburg GmbH<br>Standort Marburg<br>Baldingerstraße<br>35043 Marburg<br>Phone: +49 (0) 6421 586 6511/ -6512<br>Fax: +49 (0) 6421 586 5062<br>E-mail: burchert@staff.uni-marburg.de
**Investigational Product:**	AOP2014 as pre-filled auto-injection pen for subcutaneous injection, containing 250 µg AOP2014 / 0.5 ml. The solution also contains inactive ingredients (sodium chloride, polysorbate 80, benzyl alcohol, sodium acetate, and acetic acid). The solution is colorless to light yellow.
**Name of active ingredient:**	Pegylated-Proline-interferon alpha-2b
**Indication:**	Chronic Myeloid Leukemia
**Number of Sites:**	About 20 trial sites in Germany
**Study Objectives:**	**Primary Objective:**<br>To evaluate the efficacy of AOP2014 administered bi-weekly subcutaneously (s.c.) in preventing molecular relapse (loss of MMR) in CML patients, who discontinue ABL tyrosine kinase inhibitor therapy (TKI) in deep molecular remission of MR^4 or better ($MR^{4.5}$, or MR^5).

Secondary Objectives:

To assess tolerability and toxicity of AOP2014

To assess quality of life before and after TKI discontinuation

To evaluate the safety of maintenance therapy with AOP2014

To assess overall survival

To explore the value of 95 CD86+pDC / 10^5 lymphocytes at baseline in predicting risk of molecular relapse (loss of MMR)

Explore immunological and genetic biomarkers to study biology of TFR, and identify predictors IFN response (e.g. mRNA sequencing of whole blood or leukocyte subpopulations, PD-L1-, PD1-, CD62L- measurements by FACS on peripheral blood subsets, T-cell activation and exhaustion marker measurements, PR1-CTL assessment and cytokines). Evaluation of cytokines/chemokines (i.e., IL-6, IFN-α, IL 10, and others).

Study Design:

A randomized, open-label assessor blinded, multi-center, controlled phase II trial

Number of Patients:

214 patients have to be randomized

Test Product, Dosing, and Mode of Administration:

The investigational arm A:

The AOP2014 will be administrated by subcutaneous injection (self-administration). The pre-filled auto injection pen contains 0.5 ml of verum (equivalent to 250 µg active substance). Each pen should be used for two injections.

Study treatment will be escalated to full maintenance dose of 100µg as follows:

- 50 µg AOP2014 at day 0 (Baseline Visit) and 14 days thereafter.
- From month 2 patients will receive a full dose of 100 µg, every two weeks. First 100 µg dose has to be applied 2 weeks after the last application of 50 µg AOP2014.

There will be an overlapping treatment with AOP2014 and TKI for one month. After one month, the TKI therapy will be stopped and patient will receive only AOP2014 treatment for the next 14 months. Afterwards patient will enter the surveillance phase which will be continued until end of month 25.

The comparator arm B:

This is an open-label study with a “surveillance” group as comparator arm.

Similar as in the arm A, patient will discontinue TKI therapy one month after randomization. From then on patient will receive no further treatment.

Randomization:

Randomization will be performed centrally by the Coordinating center for Clinical Trials in Marburg. Eligible patients will be randomized in a 1:1 ratio to receive AOP2014 or surveillance by stratified randomization according to the trial site and prior failure of a discontinuation attempt (yes/no).

Screened patients who were not randomized must be registered at KKS Marburg. The reason should be noted on the respective form.

Study Population:

Criteria for inclusion:

1. Signed written informed consent form
2. Capability and willingness to comply with study procedures and ability to self-administration of the study drug
3. Male or female aged ≥ 18 years
4. At least three years of TKI therapy
5. BCR-ABL-positive, chronic phase CML patients with a transcript level according to the international scale (IS) of at least MR^4, or better ($MR^{4.5}$, MR^5). MR^4 is defined as (i) detectable disease ≤0.01% BCR-ABL IS or (ii) undetectable disease in cDNA with ≥10,000 ABL or ≥24,000 GUS transcripts for at least one year. There have to be at least three consecutive PCR-results with MR^4 or better within the last year (+ 2 months) before study entry. The latest of these PCRs must be a confirmatory MR^4 measurement prior to randomization by the EUTOS-certified Study Reference Laboratories for PCR (BCR-ABL mRNA) in Mannheim or Jena. No PCR-results in the last year before randomization can be worse than MR^4. If the last PCR was not done within last two months from baseline (day 0) in Jena or Mannheim, the PCR sample must be sent to Jena or Mannheim at screening.
6. Patients who had failed to discontinue TKI in a prior discontinuation attempt are eligible for this protocol, if they fulfil criterion 5 after retreatment with TKI. A prior TKI discontinuation failure must be specifically indicated at inclusion and documented
7. Adequate organ function: especially total bilirubin, lactate dehydrogenase [LDH], aspartate aminotransferase [AST],

alanine aminotransferase [ALT] and coagulation parameters ≤ 2 × upper limit of normal (ULN)

8. Adequate hematological parameters: platelet count $\geq 100\times10^9$/L; white blood cell count $\geq 2.5\times10^9$/L; lymphocytes $\geq 1.0\times10^9$/L; hemoglobin≥9.0 g/dL or 5.59 mmol/L
9. Female patients with reproductive potential must agree to maintain highly effective methods of contraception by practicing abstinence or by using at least two methods of birth control from the date of consent through the end of the study. If abstinence could not be practiced, a combination of hormonal contraceptive (oral, injectable, or implants) **and** a barrier method (condom, diaphragm with a vaginal spermicidal agent) has to be used. Male patients must agree to use condoms during study participation.
10. Negative serum pregnancy test in women of childbearing potential.
11. Date of diagnosis of CML confirmed by laboratory PCR must be known.

Exclusion Criteria

1. Rare variants of BCR-ABL not quantifiable by RT-PCR according to the international scale (IS)
2. Current or previous autoimmune diseases requiring treatment
3. Immunosuppressive treatment of any kind
4. Prior allogeneic stem cell transplantation
5. Prior pegylated IFN therapy. Prior low dose conventional IFN treatment with ≤ 3 x 3 Mio I.E. / week for less than 1 year is acceptable
6. History of TKI resistance within the last 4 years of TKI therapy
7. History of accelerated phase or blast crisis
8. Hypersensitivity/allergy to the active substance or excipients of the formulation
9. Severe hepatic dysfunction or decompensated cirrhosis
10. Thyroid disease that cannot be controlled by conventional therapy
11. Epilepsy or other disorders of the central nervous system

12. Severe cardiac disease history including unstable or uncontrolled cardiac disease in the previous 6 months
13. Any history of retinopathy e.g. retinal detachment, degeneration or thromboembolic events
14. Clinically significant concomitant diseases or conditions, which, in the opinion of the investigator, would lead to an unacceptable risk for the patient to participate in the study (please refer also to the actual Investigator Brochure)
15. Other malignancy, except adequately treated superficial bladder cancer, basal or squamous cell carcinoma of the skin, or other cancer(s) for which the patient has been disease free for more than 3 years
16. Active or uncontrolled infections at the time of randomization
17. Pregnant and/or nursing women
18. Use of antibiotic therapy within the last 2 weeks prior to randomization
19. Concurrent use of molecular targeted therapy
20. Tested HIV sero-positivity or tested active hepatitis B or C infection
21. Participation in another clinical study with other investigational drugs within 14 days prior to randomization
22. Vaccination within 1 month prior to randomization
23. Any medical, mental, psychological or psychiatric condition (particularly severe depression, suicidal ideation or suicide attempt) that in the opinion of the investigator would not permit the patient to complete the study or comply to study procedures
24. Drug and/or alcohol abuse

Primary endpoint: The primary efficacy endpoint is molecular relapse free survival, RFS 7 months after randomization.

**Secondary endpoints:**	• RFS 13 months after randomization<br>• RFS 25 months after randomization<br>• Safety, tolerability and toxicity based on incidences of adverse events, serious adverse events, frequency of clinical laboratory tests by worst toxicity grade<br>• Quality of life (QoL)<br>• To explore the value of 95 CD86+pDC / 10^5 lymphocytes at baseline (before TKI stop) as a predictor of RFS<br>• Overall survival<br>• Explore immunological and genetic biomarkers to study biology of TFR, and identify predictors IFN response<br>• Evaluation of cytokines/chemokines (i.e., IL-6, IFN-α, IL 10, and others)<br>• Kinetics of BCR-ABL transcript level over time after TKI stop
**Safety:**	Adverse events, serious adverse events (AEs, SAEs)
**Statistical analysis:**	Four hypotheses are tested in hierarchical order. To avoid inflation of type 1 error (false rejection of a null hypothesis), further confirmatory testing has to be stopped as soon as a null hypothesis could not be rejected. All four hypotheses are tested at significance level 0.05. Null hypotheses 1, 2, and 4 deal with probabilities of molecular relapse-free survival 7, 13, and 25 months after randomization, respectively; arms A and B are compared with the uncorrected chi-square test. Null hypothesis 3 investigates molecular relapse-free survival as a time-to-event variable; the two arms are compared with the log-rank test.
**Time Schedule:**	Recruitment period (months): 18<br>Duration of study per patient (months): 25<br>First patient in to last patient out (months): 43<br>End of the main study (months): after 49<br>Post Study Follow Up (months): 35

1.1 Study flow chart ENDURE-CML

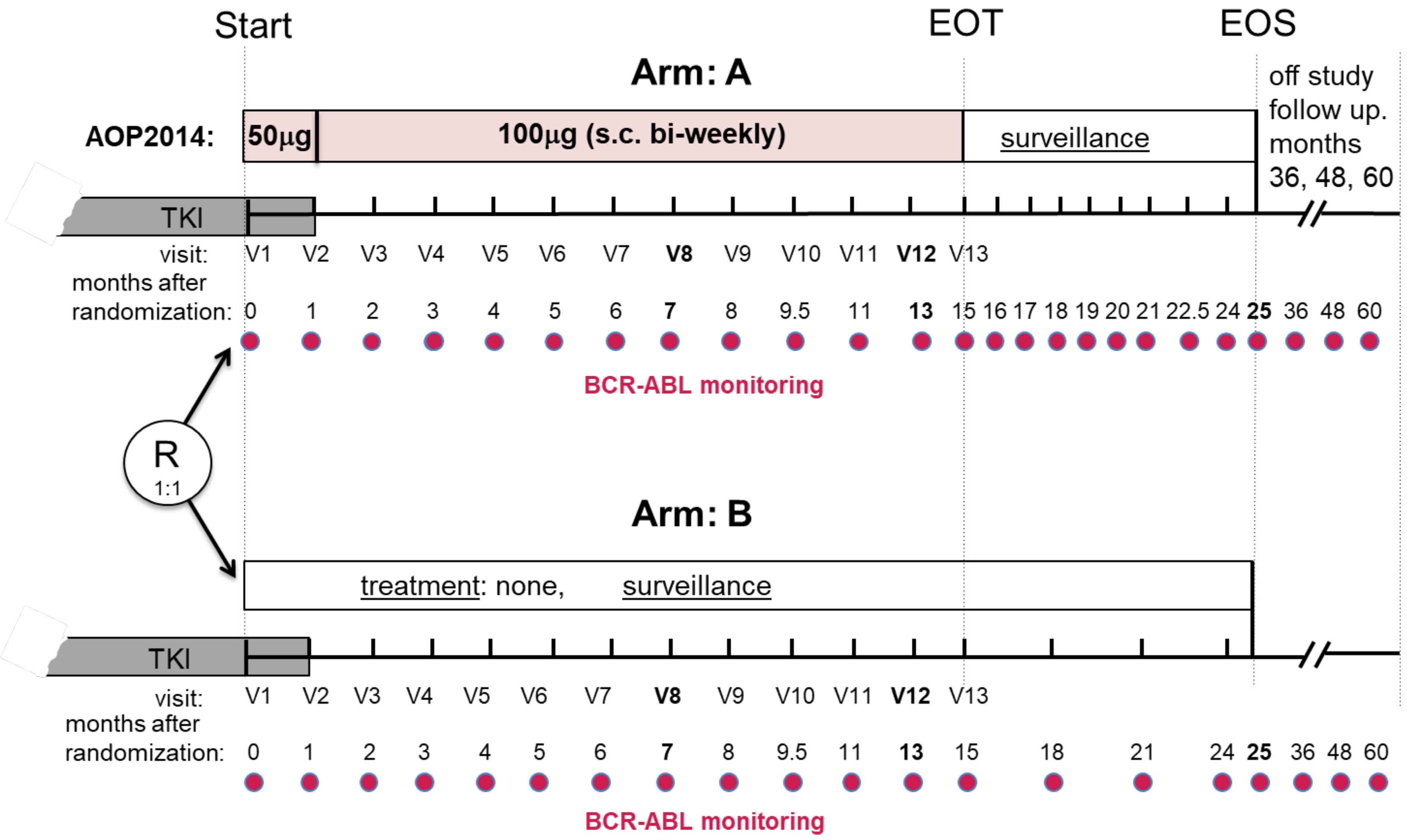

1.2 ENDURE-CML: study specific procedures for ARM A (AOP2014)

	Screening (within 2 months)	Baseline (day 0)	ARM A (AOP2014 50 µg or 100 µg s.c. / biweekly): Treatment phase											End-of-Treatment [3]	Surveillance Phase									Post Study Follow Up [9]
**Visit Number**		**1**	**2**	**3**	**4**	**5**	**6**	**7**	**8**	**9**	**10**	**11**	**12**	**13**										
**Months after randomization** [1]	**-2**	**0**	**1**	**2**	**3**	**4**	**5**	**6**	**7**	**8**	**9,5**	**11**	**13**	**15**	**16**	**17**	**18**	**19**	**20**	**21**	**22,5**	**24**	**25**	**36, 48, 60**
Informed consent	X																							
Inclusion and exclusion criteria	X																							
Serologic testing Anti-HIV, HCV+HBs	X																							
Randomization [2]		X																						
Demographic data, Sokal, EUTOS, EURO, ELTS score	X																							X
Medical history, pre-treatment history	X																							X
Physical examination / vital signs / body temperature	X	X	X	X	X	X	X	X	X	X	X	X	X	X	X		X			X		X	X	
ECOG PS	X	X	X	X	X	X	X	X	X	X	X	X	X	X	X		X			X		X	X	
Safety laboratory analyses [4]	X	X	X	X	X	X	X	X	X	X	X	X	X	X	X									
Blood Count																X	X	X	X	X	X	X	X	
BCR-ABL transcripts (IS) [5]	X	X	X	X	X	X	X	X	X	X	X	X	X	X	X	X	X	X	X	X	X	X	X	X
Pregnancy testing [6]	X	X	X	X	X	X	X	X	X	X	X	X	X	X	X									
Autoimmunity (ANA, TSH) screen	X		X						X					X										
Serum for immune parameters		X							X					X										
Cellular Immune-Marker (FACS) and stem cell persistence [7]		X	X						X					X	X									
IFNa prediction scores [8](PB)		X	X						X			X		X										
TKI intake for 30 days		X																						
AOP2014 dosing (biweekly, **50 µg for the first 4 weeks**, from **month 2 full dose of 100 µg**) and accounting		**50 µg**	**100 µg**																					
		X	X	X	X	X	X	X	X	X	X	X	X	X										
Local tolerability assessment		X	X	X	X	X	X	X	X	X	X	X	X	X										
Quality of life		X	X	X	X	X	X	X	X	X	X	X	X	X										
Concomitant treatment	X	X	X	X	X	X	X	X	X	X	X	X	X	X	X		X			X		X	X	
Adverse events	X	X	X	X	X	X	X	X	X	X	X	X	X	X	X									

Table 1 Study specific procedures for ARM A

GERMAN CML.STUDY GROUP

1.3 ENDURE-CML: study specific procedures for ARM B (surveillance)

	Screening (within 2 months)	Baseline (day 0)	ARM B (surveillance)											End-of-Treatment [3]	Surveillance Phase				Post Study Follow Up [9]
**Visit Number**		**1**	**2**	**3**	**4**	**5**	**6**	**7**	**8**	**9**	**10**	**11**	**12**	**13**					
**Months after randomization** [1]	**-2**	**0**	**1**	**2**	**3**	**4**	**5**	**6**	**7**	**8**	**9,5**	**11**	**13**	**15**	**18**	**21**	**24**	**25**	**36, 48, 60**
Informed consent	X																		
Inclusion and exclusion criteria	X																		
Serologic testing Anti-HIV, HCV+HBs	X																		
Randomization [2]		X																	
Demographic data, Sokal, EUTOS, EURO, ELTS score	X																		X
Medical history, pre-treatment history	X																		X
Physical examination / vital signs / body temperature	X	X	X	X	X	X	X	X	X	X	X	X	X	X	X	X	X	X	
ECOG PS	X	X	X	X	X	X	X	X	X	X	X	X	X	X	X	X	X	X	
Safety laboratory analyses [4]	X		X	X	X	X	X	X	X	X	X	X	X	X	X				
Blood Count																X	X	X	
BCR-ABL transcripts (IS) [5]	X	X	X	X	X	X	X	X	X	X	X	X	X	X	X	X	X	X	X
Pregnancy testing [6]	X																		
Autoimmunity (ANA, TSH) screen	X													X					
Serum for immune parameters		X							X					X					
Cellular Immune-Marker (FACS) and stem cell persistence [7]		X	X						X					X	X			X	
IFNa prediction scores [8] (PB)		X	X						X			X		X				X	
TKI intake for 30 days		X																	
Quality of life		X	X	X	X	X	X	X	X	X	X	X	X	X				X	
Concomitant treatment	X	X	X	X	X	X	X	X	X	X	X	X	X	X	X	X	X	X	
Adverse events	X	X	X	X	X	X	X	X	X	X	X	X	X	X					

Table 2 Study specific procedures for ARM B

ANA = antinuclear antibodies; ECOG PS = Eastern Cooperative Oncology Group performance score; FACS = fluorescence activated cell sorting; IS = international scale; TKI = ABL-specific tyrosine kinase inhibitor (e.g., imatinib, dasatinib, nilotinib)

Legend to table 1a and table 1b

1. Visits can be pre- or postponed by 7 days.
2. Randomization could be done up to two working days prior to potential study treatment start. If the patient will not meet all the inclusion criteria / fulfill the exclusion criteria, or will withdraw his informed consent before the randomization, he/she must be registered by KKS Marburg using the Screening/Randomization form
3. A pre-mature end of treatment (EOT) assessment must be performed before months 15 for reasons outlined in section 13 and latest at the scheduled next visit. A regular end of treatment visit can be pre- or postponed by 10 days.
   IMPORTANT: a premature end of treatment, in case of molecular relapse (loss of MMR) or withdrawal of informed consent, irrespective of whether occurring in arms A or B requires to immediately resume TKI therapy. **After the premature EOT Visit has been performed, a molecular BCR-ABL follow up every three months until month 25 is required.** After month 25 off study follow ups will occur annually, as indicated in the flow chart.
4. Safety laboratory analysis should not be older than 7 days before start of study treatment with AOP2014 in arm A. If the Baseline Visit (V1) will not be performed within 1 week from Screening Visit, the safety laboratory analyses assessments have to be repeated on V1 for arm A. This includes: complete blood counts including differential blood count (machine), Na, K, Ca, Krea, Urea, GOT, GPT, AP, GGT, LDH, Bilirubine, total Protein, PT, PTT, Glucose (see 12.1.2 and 12.2.8).
5. BCR-ABL PCR must be performed at one of the two EUTOS-certified laboratories named in section 12.2.10: 20 – 30 ml blood are required.
   BCR-ABL PCR must have been confirmed three times with at least MR^4 please refer to inclusion criterium number 5.
6. For every visit HCG from serum only in Arm A in women of childbearing potential.
   Additional HCG urine test for home use > 28 days only in Arm A in women of childbearing potential.
7. For immune / stem cell marker 30ml (CD86+pDCs) of heparin and 5ml Serum are required (see 12.2.11). Shipment of samples to Marburg should be performed Monday-Thursday and is possible by regular mail.
8. For interferon alpha (IFN) response and adverse event prediction scores (see 12.2.11), please send 9 to 10 ml EDTA peripheral blood (PB) by regular mail to Aachen.
9. PSFU: Patients, who are in TFR 25 months after randomization, are recommended to undergo quantitative BCR-ABL RT-PCR (IS) monitoring every three months. However, an “off study” documentation of molecular remission will be required only once yearly for three years, namely 36, 48 and 60 months after randomization.
   For all patients, the following data will be documented in PSFU:
   a) Survival (y/n)
   b) Type of current treatment for CML
   c) Demographic data including treatment history and relevant medical history (secondary malignancies, cardiac disease, auto-immune disease, progression of CML)
   d) BCR-ABL RT-PCR (IS)

2 Introduction

2.1 Background

2.1.1 Chronic myeloid leukemia

Chronic myeloid leukemia (CML) is caused by the BCR-ABL chromosomal translocation, which results from a reciprocal translocation between the long arms of chromosomes 9 and 22. The emerging fusion protein $p210^{BCR-ABL}$ constitutively activates multiple oncogenic signalling pathways, leading to the expansion of immature and mature myeloid cells in bone marrow and blood[1,2]. The natural course of the disease is characterized by progression from a benign, chronic phase with leucocytosis, thrombocytosis and left shifted peripheral blood to acceleration and blast crisis[3]. The progressed phases are associated with a poor prognosis and cure at this stage can only be achieved with allogenic stem cell transplantation[4].

2.1.2 CML treatment with tyrosine kinase inhibitors (TKI)

Conventional standard therapy of CML is the treatment with ABL-specific TKI. In a randomized comparison between imatinib and the previous standard therapy, interferon alpha (IFN) in the IRIS trial[5], imatinib was significantly superior to IFN, both, in terms of tolerability and efficacy (i.e., durable cytogenetic treatment response). Subsequently, second generation TKI were introduced into CML therapy in order to improve tolerability and to further increase efficacy. In two large randomized trials, nilotinib, a 20-times more potent ABL-kinase inhibitor than imatinib, showed improved early molecular response rates and less progression to accelerated phase and blast crisis [6]. In analogy, dasatinib, which is approximately 300-times more potent than imatinib, also demonstrated improved cytogenetic remission rates after 12 months of treatment and deeper and sustained molecular remissions compared to imatinib [7].

2.1.3 Molecular response under TKI therapy

Response to TKI therapy is monitored by cytogenetic and / or real-time quantitative PCR monitoring according to the revised criteria of the European Leukemia Net (ELN) [8]. In brief, BCR-ABL minimal residual disease (MRD) level are quantitated as a BCR-ABL level according to the international scale (IS) [9,10]. After 12 months of treatment, a molecular remission at the level of below or equal to 0.1% (IS) - referred to as major molecular remission (MMR) - is considered as optimal response. A deeper molecular remission (MR) - that is, $<0.01\%$ (MR^4), $<0.0032\%$ $MR^{4.5}$ or less (MR^5) - indicates persistence of less CML MRD, but only limited evidence suggests that a remission depth better than MMR could be associated with improved clinical outcomes such as better progression free or overall survival [11]. However, achieving MR^4, $MR^{4.5}$ or MR^5 is clinically meaningful, because it qualifies for TKI discontinuation trials.

2.1.4 TKI discontinuation in CML in deep molecular remission

Because CML stem cells are TKI-insensitive [12,13], conventional TKI treatment in CML must be indefinite in order to prevent recurrence of disease. Indeed, first imatinib discontinuation attempts in CML patients with undetectable BCR-ABL MRD, relapse occurred rapidly [14]. However, some patients still may successfully discontinue imatinib[15]. The first systematic prospective investigation of TKI treatment discontinuation in CML was performed by the STIM-study. 41% of the patients who discontinued imatinib with undetectable BCR-ABL MRD remained durably free of relapse for now over 4 years [16]. These results were confirmed in the A-STIM and TWISTER studies. In the smaller TWISTER study, which used similar inclusion and relapse criteria as the STIM study, relapse rate was 53% after a median of 30-41 months [17]. No relapses occurred after 24 months. For patients who were 2 years or longer in at least $MR^{4.5}$ at the time of stopping imatinib, the relapse rate after 12 months was 36% in the A-STIM trial. However, in A-STIM the definition of relapse was less strict compared to TWISTER and STIM. Relapse in A-STIM was defined as the loss of MMR (BCR-ABL >

0.1%, IS) [18], whereas relapse in TWISTER and STIM was defined as any loss of $MR^{4.5}$ [17] or loss of "undetectable" BCR-ABL mRNA [16]. **Thus, approximately 40% of the patients who discontinue TKI after $MR^{4.5}$ will relapse**. $MR^{4.5}$ was an essential inclusion criterion in the aforementioned TKI discontinuation studies, but only approximately 40 to 45% of all chronic phase CML patients will achieve $MR^{4.5}$ after 8 to 9 years of imatinib treatment [11,19]. When considering also early progressions due to resistance, this translates into a **proportion of approximately only 15% of all newly diagnosed CML patients who will eventually become durably treatment free after TKI discontinuation**.

2.2 Interferon alpha to improve the rate of durable TKI discontinuation

Most CML patients relapse if TKI treatment is withdrawn. Treatment modalities that would improve the proportion of patients who can successfully discontinue imatinib are currently unknown.

Until the advent of imatinib, IFN was used as standard therapy in chronic phase CML patients. In contrast to imatinib, IFN induces complete cytogenetic remissions (CCyR) in only a small minority of CML patients [20-23]. IFN is also significantly less potent in the induction of molecular remissions when compared to imatinib [24]. In contrast to TKI-induced remissions, optimal molecular responses under IFN will rarely, if ever, reach a level of deep molecular response (e.g. $MR^{4.5}$) [25]. In spite of this, successful therapy discontinuation has frequently been observed with patients in IFN-induced CCyR [26,27]. In fact, a BCR-ABL/ABL ratio of below 0.045%, which would correspond approximately to BCR-ABL levels in the range of an MMR measured in the standardized international scale BCR-ABL PCR, was predictive for a very high rate of relapse free survival (RFS) after IFN therapy discontinuation [25]. In contrast, patients lacking MR^4 or $MR^{4.5}$ under TKI therapy have no reasonable chance to stay free of molecular recurrence after TKI discontinuation; consequently these patients were excluded from previous TKI discontinuation studies. Indeed, even if a sustained MR^4 / $MR^{4.5}$ was reached, a molecular relapse occurs in approximately 40-60% after stopping imatinib [14-18,28,29].
Thus, there is evidence that mechanisms of remission induction and maintenance differ significantly between TKI- versus IFN-induced remissions [30-33]. It was therefore rational to attempt to complement suspected different modes of action of TKI and IFN within TKI/IFN combination therapy studies [34-37]. Two of these trials, which used pegylated forms of IFN together with imatinib reported a significantly improved early molecular response with the combination [35,36]. A more recent trial combining nilotinib and pegIFN confirmed this result and showed high $MR^{4.5}$ rates within the first year of treatment [38].
We have previously shown that CML patients receiving an upfront imatinib/IFN combination therapy show low rates of early progression and a trend towards deepening of their molecular remission despite the absence of selective Abl-kinase inhibition [39].
After a median of 8-years of follow up this patient cohort presented a molecular relapse-free survival (loss of MMR) of 73% (8/11 pts.) and 84% (5/6 pts.) for patients who discontinued imatinib in MMR and MR^4/$MR^{4.5}$, respectively. Ten patients discontinued IFN after a median of 4.5 years (range, 0.24 to 9.3). After a median of 2.8 years (range, 0.7-5.1) nine of them remain in ongoing treatment-free remission with MR^5 (n=6) and $MR^{4.5}$ (n=3) [40].
Thus, IFN seems to cooperate with TKI in the induction of molecular remissions and may promote the maintenance of deep molecular remissions without further TKI treatment. The underlying mechanisms of IFN activity are not well understood, but may involve immune processes. IFN, but not TKI has been suggested to elicit anti-leukemic immune responses [30-32].
IFN-mediated control of BCR-ABL-positive MRD would explain, why successful therapy discontinuation with high MRD levels (2-3 logs higher than in the STIM trials) has only been observed in IFN patients [25,27] but never with TKI.

However, IFN toxicity can limit its applicability [41,42]. To overcome this problem, better tolerable and also more efficacious pegylated IFN 2a and IFN 2b formulations were introduced. In a phase II study

pegylated IFN 2a doubled the rate of CCyR [42]. When combined with imatinib, 90 µg pegylated IFN 2a weekly or 50 µg pegylated 2b were more effective in terms of inducing molecular remissions than imatinib alone [35,36]. Nevertheless, within the first 12 months of combination therapy a significant proportion of the patients in these two trials discontinued IFN due to intolerance [35,36].
Tolerability may be significantly improved by lowering the doses. In a the above mentioned Nilotinib / pegIFN trial, a pegIFN2a dose of 45 to 90 µg weekly was well tolerated (with 73% remaining on treatment after 12 months) and efficacious [38]. The minimally required, effective IFN dose has never been systematically defined in CML. Our recent study showed that much lower doses of IFN than conventionally used in previous studies may be sufficient to exert IFN´s beneficial effects [40]. The duration of maintenance therapy is also not defined. In our cohort study, the median duration of IFN maintenance therapy was 54 months [40] - longer than perhaps needed. On the other hand, in a small study on 6 patients, a median of five months of IFN maintenance was ineffective [43]. Previous recommendations from the M.D. Anderson Cancer Center and a large European registry survey suggested that conventional IFN therapy should be continued for two to three years after obtaining a complete cytogenetic remission with IFN monotherapy [44, 26]. However, Hochhaus et al. have shown that not the duration of maintenance treatment, but rather the depth of remission below at least MMR achieved with IFN treatment is critical to predict relapse risk [25].

2.3 Pegylated-Proline-Interferon alpha-2b (AOP2014)

2.3.1 Pharmacokinetics

The pharmacokinetic and pharmacodynamic profile of AOP2014 has been investigated in a study in cynomolgous monkeys (4/group). In the first phase, the pharmacokinetic and pharmacodynamic profiles of AOP2014 were compared with PegIFN 2a (Pegasys®) when administered subcutaneously (30 µg/kg). In the second phase, the pharmacokinetic and pharmacodynamic profiles of AOP2014 were evaluated following intravenous (30 µg/kg) and subcutaneous (300 µg/kg) administration.
There were no treatment related clinical signs or significant changes in body weight. In Phase 1, AOP2014 exhibited a pharmacokinetic profile similar to Pegasys®. In Phase 2, intravenous administration of AOP2014 at the same dose resulted in greater serum concentration of AOP2014 from 2–96 hours in comparison to subcutaneous administration in Phase 1. Relative bioavailability of AOP2014 was calculated to be 80%. Subcutaneous administration of AOP2014 at 300 µg/kg led to even higher AOP2014 concentrations at 6 to 144 hours post dose. Relative bioavailability was 42%.
In healthy adult male patients, AOP2014 reached maximal plasma concentrations (1.8 to 21.3 ng/mL) after 75 to 90 hours (tmax) when 24 to 225 µg had been applied subcutaneously (n = 4 to 5). The tmax values observed did not show any dose dependence. However, after subcutaneous administration of 270 µg maximal levels (24.8 ng/m) were seen after 116 hours (n = 5). With subcutaneous doses of 24 to 225 µg, the elimination half-life was determined as 61 to 92 hours with no obvious dose dependence (n = 4 to 5). However, after a subcutaneous dose of 270 µg the elimination half-life was 118 hours (n = 5).
The prolonged plasma half-life and the resulting increase in AUC is hoped to result in an improved therapeutic window of peg-IFN-alpha-2b.

2.3.2 Clinical experience with AOP2014

The Phase I/II study of AOP2014 in PV is currently ongoing (completed recruitment of 51 patients and collecting follow-up information on efficacy and safety). The AOP2014 formulation with the strength 0.18 mg/ml is being used in this phase I/II study. The maximum tolerated dose (MTD), long term safety and efficacy of AOP2014, administered subcutaneously every 14 days, are the main objectives of the study. Patients with confirmed PV diagnosis, age equal or older than 18 years, both naïve and cytoreduction pre-treated were eligible. After establishing the MTD, an extended cohort of 25 additional patients was planned to be recruited.

European LeukemiaNet criteria were used for response assessment. Interim analysis of efficacy and safety data was presented at the last, 2012 ASH annual meeting (Gisslinger et al, 2012, oral presentation and abstract). 34 patients were included into this analysis, 25 in Phase I (dose-finding) and 9 in the Phase II (cohort extension).

Median time from diagnosis was 24 months (range 0–180). 12 patients (35%) had been pre-treated with HU (mean past duration of HU pre-treatment 39 months, mean daily HU dose 950 mg). Median number of phlebotomies in the past 3 months prior to inclusion was 1 (range 0–8), a total of 21 patients (62%) were regularly phlebotomized at least once in three months prior to study entry. 11 patients (32%) had a history of thrombotic complications. Median Hct at baseline was 42% (range 36–51). Median WBC and platelet counts were 10.6*109/l (range 3.9–20.4) and 452×109/l (range 141–1019), respectively. 17 patients (50%) had splenomegaly at baseline. The median reported treatment duration was 41 weeks (range: 1 day – 80 weeks), 11 patients completed 1 year on treatment. Doses from 50 to 540 µg every two weeks were tested, the highest tested dose, *i.e* 540 µg was determined as MTD since no DLTs occurred in the study. The mean administered dose (both Phase I and II patients) was 287 µg. After 28 weeks of treatment (21 evaluable patients), 71% of patients had hematological response (7 CR, 33%; 8 PR, 38%), at week 36 (19 evaluable patients) 8 patients (42%) achieved a CR and 8 patients (42%) a PR, overall response rate (ORR, CR+PR) was 84%. At week 52 (1 year; 11 evaluable patients), 5 patients (46%) had CR and 5 (46%) PR, ORR was 91%; 8 (73%) patients presented with completely normalized blood values, all evaluable patients did not require phlebotomy at this time point. 4 patients (of 12 evaluable for this measurement, 33%) still had an enlarged spleen at week 52. At week 76, 2 evaluable patients were complete responders. At week 52, 1 patient (of 9 evaluable, 11%) developed partial molecular response, at week 68 3 patients (of 7 evaluable, 43%) had partial molecular response. One patient with allelic burden of 22% at baseline developed complete molecular response at week 36 (still ongoing). Mainly grade 1 and 2 adverse events were reported. A total of 358 adverse events occurred. 27 patients (79%) suffered from drug-related adverse events. 9 patients (26%) developed serious adverse events; 4 SAEs were considered to be treatment related. 5 patients (15%) discontinued their study participation prematurely, 3 of them due to adverse events (deterioration of underlying disease and two cases of depression). Acceptable tolerability and sustained clinical benefits have been demonstrated in PV patients measured as overall response rate of above 90% with CRs of 46% at one year after treatment start. Most of the patients did not require phlebotomy and their hematological parameters fell with the normal range.

Based on these results the phase III randomized study PROUD-PV has previously been initiated. The aim of the study is to provide confirmatory evidence that AOP2014 is non-inferiority to HU, as measured by normalization of blood parameters and spleen size. If this aim is achieved, the study will be used as a pivotal, registration study to apply for marketing authorization of AOP2014 in patients with PV. Afterwards there was performed the CONTINUATION-PV and PEN-PV study. CONTINUATION-PV Study is an open-label, Phase IIIb extension study assessing the long-term efficacy and safety of AOP2014 in PV patients who completed the AOP2014 arm of the PROUD-PV Study in comparison to patients who completed the HU arm of PROUD-PV Study and are managed according to standard of care/best available treatment.

PEN-PV Study is an open-label, single-arm Phase III study to assess the self-administration of AOP2014 using the pre-filled pen intended for commercial use in 36 patients with PV.

In 2011, orphan designation for Ropeginterferon alfa-2b (AOP2014) for the treatment of PV was granted to AOP by the European Commission.

2.4 Rationale for the study

Current standard therapy in CML is indefinite TKI treatment. This is frequently associated with side effects, unknown long-term toxicities and also significant costs. There is currently no known treatment modality, which improves the proportion of patients who can successfully discontinue TKI therapy.

1. There is a significant medical need to identify treatment concepts in CML, which enable a discontinuation of an otherwise life-long therapy (with TKI). As eluted in section 2, mounting evidence suggests that a pegylated formulation of IFN could by such a substance. It exerts a substantial synergism with TKI and also has the potential to induce a durable TFR, when applied sequentially after upfront TKI therapy. Based on this rationale, the primary aim of this study will be to increase the proportion of patients, who can permanently discontinue TKI treatment using temporary AOP2014 maintenance therapy, which will be started after stopping the TKI. Obviously, only TFR is of maximum value to patients, because it will avoid any chronic side effects and long-term toxicities of TKI, and significant annual costs (approx. 30-50.000 €).

2. There is currently no other established or experimental treatment, which has been shown to increase the rate of sustained deep molecular remissions after TKI discontinuation in CML (see 2.3). This makes this study unique.

3. The study will provide unprecedented biological insights into the effects of AOP2014 in controlling minimal residual stem cell disease induced by long-term prior TKI therapy. It will also enable to better understand mechanism of immune responses induced by IFN to control CML.

4. If the study would be positive, the findings could become treatment-changing for patients in deep molecular remission under TKI and willing to tolerate a temporary additional treatment.

2.5 Risk benefit assessment

Patients, who will enter this study, will benefit from a 50% chance to durably discontinue TKI therapy. TKI discontinuation itself is very safe. There is no risk of disease progression after TKI stop and no risk of failure to regain deep molecular remission, in the event of MMR loss. Restarting with the previous TKI will essentially always restore deep molecular remission. This evidence is based on large clinical study cohorts of more than 1.000 documented patients[15-18,45,46].
Secondly, IFN was the previous standard drug in CML. It is a safe drug and, as eluted in 2.2 and 2.4 has previously been shown - in uncontrolled studies - to enable induction of a durable TFR. It offers therefore a reasonable chance to increase the overall rate of TFR in CML patients.

Due to the longstanding experience with the therapeutic use of human IFN in the treatment of diverse malignancies (melanoma, lymphoma, CML) and infectious diseases (hepatitis C) IFN can be considered as a safe drug. Potential toxicities and side effects are well predictable and consist mainly of temporary flu-like symptoms. However, rarely, there are also significant side effects such as induction of auto-immune diseases (thyroiditis, vitiligo, sarcoidosis etc.). Hair loss, psychiatric symptoms and other auto-immune symptoms may also occur. All side effects can usually be controlled by dose reductions or treatment discontinuation. Toxicities require extremely rarely active treatment intervention.

This altogether suggests that benefits associated with a durable TKI discontinuation induced by AOP2014 by far overweigh potential risks associated with AOP2014 treatment.
Any indication that AOP2014 maintenance, will negatively affect relapse rates or progression after TKI discontinuation cannot be identified, further underscoring the positive benefit/risk ratio.

3 Study Objectives

The purpose of the study is to evaluate the efficacy and safety of pegylated-proline-interferon alpha-2b (AOP2014) administered biweekly for a maximum of 15 months in patients with chronic phase of CML, who discontinued TKI treatment with a residual BCR-ABL mRNA level of at least MR^4.

3.1 Primary objective

The primary objective of this study will be to evaluate the efficacy of AOP2014 administered bi-weekly subcutaneously (s.c.) in preventing molecular relapse (loss of MMR) in CML patients, who discontinue ABL tyrosine kinase inhibitor therapy (TKI) in deep molecular remission of MR4 or better (MR4.5, or MR5).

3.2 Secondary objectives

- To assess tolerability and toxicity of AOP2014.
- To assess quality of life before and after TKI discontinuation.
- To evaluate the safety of maintenance therapy with AOP2014
- To assess overall survival.
- To explore the value of 95 CD86+pDC / 10^5 lymphocytes at baseline in predicting risk of molecular relapse (loss of MMR)
- Carry out immunological and genetic biomarker studies to study biology of TFR, and identify predictors IFN response (e.g. by mRNA sequencing of whole blood or leukocyte subpopulations, PD-L1-, PD1-, CD62L- measurements by FACS, T-cell activation and exhaustion marker measurements)

All patients are eligible to continue observation in the Post-Study Follow-up phase.

3.3 Post Study Follow Up (PSFU) Objectives

- Documentation of durability of rate of TFR and RFS (BCR-ABL molecular remission status according to IS)
- Assessment of overall survival (OS)
- Descriptive: type of current treatment for CML, if TFR was lost (demographic data including treatment history)

4 Study Endpoints

4.1 Primary endpoint

The primary efficacy endpoint is molecular relapse free survival, RFS 7 months after randomization.

Relapse is defined as loss of major molecular remission, MMR, which is any increase of the BCR-ABL ratio to > 0.1% according to the international scale (IS). Time to relapse is defined as the time from randomization to relapse.

4.2 Secondary endpoints

- RFS 13 months after randomization
- RFS 25 months after randomization
- Safety, tolerability and toxicity based on incidences of adverse events, serious adverse events, frequency of clinical laboratory tests by worst toxicity grade
- Quality of life (EORTC QLQ-C30 and EORTC-QLQ-CML24 questionnaires) The QoL assessment in this study is planned to gain information on the QoL of CML patients under stopping conditions. The data will be compared between the treatment groups and to QoL of normal population. Furthermore, results of the CML24 module should be shared with the EORTC group to complete the validation of this questionnaire
- Explore the value of 95 CD86+pDC / 10^5 lymphocytes at baseline (before TKI stop) as a predictor of TFR
- Overall survival (OS), defined as the time between the date of randomization and the date of death from any cause.
- Explore immunological and genetic biomarkers to study biology of TFR, and identify predictors IFN response (e.g. mRNA sequencing of whole blood or leukocyte subpopulations, PD-L1-, PD1-, CD62L- measurements by FACS on peripheral blood subsets, T-cell activation and exhaustion marker measurements, PR1-CTL assessment and cytokines).
- Evaluation of cytokines/chemokines (i.e., IL-6, IFN-α, IL 10, and others).
- Kinetics of BCR-ABL transcript level over time after TKI stop

5 Post study Follow Up endpoints

- Molecular relapse-free survival 36, 48 and 60 months after randomization for patients in arm A and B, who were in TFR at the end of month 25.

5.1 Secondary endpoints

- Comparison of overall survival (OS) in arms A and B, defined as the time between the date of randomization and the date of death from any cause
- Type of treatment (arms A and B)
- Comparison of demographic data including treatment history and relevant medical history (secondary malignancies, cardiac disease, auto-immune disease, progression of CML)

6 Randomization

Randomization in this study can be ethically and medically justified. First, randomization into surveillance with no further CML-specific treatment (arm B) is justified because of 2.1.4, and because there is currently no treatment modality known to improve relapse rates after TKI discontinuation. Randomization into the AOP2014 maintenance arm (arm A) is justified, because of available evidence showing that IFN may improve relapse rates [39,40].

Randomization will be performed centrally by faxing the randomization form to the:

**Koordinierungszentrum für klinische Studien (KKS)**
**Philipps-University Marburg**
**Karl-von-Frisch-Straße 4**
**D-35043 Marburg**
**Monday-Thursday 8.00–16.00 h**
**Friday 08.00–14.00 h**
**FAX: +49(0)6421-28 66516**
**Tel.: +49(0)6421-286 6456**

Randomization will not be possible during public holiday.
The randomization of an eligible patient can take place if all inclusion criteria and none of the exclusion criteria are fulfilled. Therefore the investigator completes the study specific randomization form, which is a part of the Investigator's Folder (ISF), and sends it to KKS Marburg *via* fax. In this form the investigator has to fill in also the Patient-ID (Pat-ID). Pat-ID is combined from the site number (2-digit, starting with 01) and a sequencing number of the patients starting with 001 for each site. This unique Pat-ID is used for all documentation on e-CRF and DCF.

KKS Marburg reports the randomization result back to the center.

The chance for randomization to the investigational arm or the standard arm is 1:1. Permuted block randomization will be used, stratified by:

- site
- prior failure of a TKI discontinuation attempt (yes/no)

7 Blinding

This is an open-label assessor blinded study. Subjects and investigators will be not blinded to the treatment assignment. A placebo-controlled randomization was not considered meaningful, because flu-like side effects regularly occur and would make a blinded, placebo-controlled treatment futile.

8 Investigational Plan

8.1 Overall Study Design

This clinical study is a randomized, multi-center, phase II, open-label assessor blinded study design with "surveillance" as comparator arm (see 1.1).

8.2 Discussion of Study Design

8.2.1 TKI discontinuation

TKI discontinuation is currently not a recommended standard in deep molecular remission of CML. However, TKI discontinuation can be recommended within clinical trials. First, because it has previously been shown in clinical trials that TKI discontinuation is safe, as long as adequate molecular monitoring can be ensured and thus re-commencing TKI after loss of CMR (complete molecular remission) or MMR [15-18]. Secondly, stopping a TKI in $MR^{4.5}$ offers a chance of approximately 50-60% to durable discontinues a TKI (see 2.1.4).

8.2.2 Initial combined TKI and AOP2014 treatment in arm A

The reason for an overlapping AOP2014/TKI treatment during the first month would be cause T-cell activation and dendritic cell maturation before Abl-kinase activity is released. It is expected that concomitant therapy before TKI discontinuation enables also a better adaption to initial AOP2014 side effects before mono-therapy start. This might translate also in increased compliance to AOP2014 monotherapy.

8.2.3 AOP2014 as maintenance therapy in molecular remission

Pegylated-Proline-interferon alpha-2b (AOP2014) will be used as maintenance therapy in CML, because available literature suggests an important activity of this compound in the treatment of CML [35,38,42 39,40]. As mentioned above (see 2.2) the optimal dose of pegIFN formulations (pegIFN 2a or pegIFN 2b) and of AOP2014 has not been defined in CML. Also the optimal treatment duration of IFN in CML is unknown.

A combination of 90 μg pegIFN 2a weekly resulted in improved molecular response rates compared to imatinib alone. At the reduced dose of 45 μg pegIFN 2a was better tolerated and effective[38].

Thus, the maintenance dose of **100 μg AOP2014 s.c. biweekly**, equivalent to approximately 70 μg of pegIFN 2a (Pegasys®) weekly, will be used as the standard dose in ENDURE-CML Study.

8.2.4 AOP2014 treatment duration

The optimal duration of IFN therapy to maintain deep molecular remission induced by TKI induction therapy is unknown. In a small uncontrolled study, 6 months of IFN maintenance after TKI discontinuation were ineffective in sustaining TFR [43]. On the other hand, an IFN maintenance duration of more than 4 years may not be needed [40]. In CML, high avidity anti-leukemic T-cell clones are depleted at de novo diagnosis, but can be re-shaped in remission after longer therapy [31,32]. For example, IFN treatment of at least two to three years restored the skewed T-cell receptor repertoire in hairy cell leukemia [47] and stimulated expansion of specific, high avidity anti-leukemic T-cell clones in complete cytogenetic responders [32]. Since IFN-induced cytogenetic remissions emerge significantly more slowly than with imatinib (in median more than 12 months after commencing treatment [24,48]) the minimum effective IFN maintenance treatment duration is estimated to be probably between 1 and 2 years.

Patients in arm A are therefore treated with AOP2014 for 15 months and then observed for an additional 10 months without any therapy. Since most patients relapse within 6 months off any TKI (only 7% of all relapses occurred after months +9 after TKI discontinuation in the A-STIM trial[18]), the evaluation of RFS 25 months after randomization (last visit) will enable an objective judgment about the efficacy of AOP2014 in preventing relapses after TKI discontinuation.

8.2.5 Post Study Follow Up (PSFU)

In order to assess long-term sustainability of remissions in both arms, an annual off-study molecular and clinical follow up and e-CRF documentation is deemed appropriate for additional 3 years.
This follow phase is purely observational. The assessments performed during this time do not differ from routine standard of care procedures.

8.2.6 Expected treatment improvement by AOP2014

The required study population was calculated to show an improvement in relapse free survival of 20% with AOP2014 maintenance compared to the surveillance arm. This would be clinically meaningful and clinically relevant.

9 Study Population

CML patients having been treated with TKI for at least three years and being in at least MR4 or better MR will be informed by the investigator about the purpose, risks and benefits of the ENDURE-CML study.

Eligible female patients with childbearing potential will continue to use highly effective methods of contraception as advised and standard for TKI treatment

9.1 Definition of deep molecular remission

Definition of deep molecular remission is as follows:

1. MR4 (MR4) (either (i) detectable disease ≤0.01% BCR-ABL (IS) or (ii) undetectable disease in cDNA with ≥10,000 ABL ≥ 24,000 GUS transcripts)
2. MR4.5: either (i) detectable disease ≤0.0032% BCR-ABL (IS) or (ii) undetectable disease with in cDNA with ≥32,000 ABL or ≥ 77,000 GUS transcripts
3. Samples with a total of <10,000 or <32,000 ABL transcripts (< 24,000 or <77,000 GUS transcripts) (i.e. sum of the replicates if replicate analysis is performed) should be considered as inevaluable for MR4.0 or MR4.5, respectively

9.2 Inclusion Criteria

1. Signed written informed consent form
2. Capability and willingness to comply with study procedures and ability to self-administration of the study drug
3. Male or female aged ≥ 18 years
4. At least three years of TKI therapy
5. BCR-ABL-positive, chronic phase CML patients with a transcript level according to the international scale (IS) of at least MR^4, or better ($MR^{4.5}$, MR^5). MR^4 is defined as (i) detectable disease ≤0.01% BCR-ABL IS or (ii) undetectable disease in cDNA with ≥10,000 ABL or ≥24,000 GUS transcripts for at least one year. There have to be at least three consecutive PCR-results with MR^4 or better within the last year (+ 2 months) before study entry. The latest of these PCRs must be a confirmatory MR^4 measurement prior to randomization by the EUTOS-certified Study Reference Laboratories for PCR (BCR-ABL mRNA) in Mannheim or Jena. No PCR-results in the last year before randomization can be worse than MR^4. If the last PCR was not done within

last two months from baseline (day 0) in Jena or Mannheim, the PCR sample must be sent to Jena or Mannheim at screening.

6. Patients who had failed to discontinue TKI in a prior discontinuation attempt are eligible for this protocol, if they fulfil criterion 5 after retreatment with TKI. A prior TKI discontinuation failure must be specifically indicated at inclusion and documented.
7. Adequate organ function: especially total bilirubin, lactate dehydrogenase [LDH], aspartate aminotransferase [AST], alanine aminotransferase [ALT] and coagulation parameters ≤ 2 × upper limit of normal (ULN)
8. Adequate hematological parameters: platelet count ≥100×109/L; white blood cell count ≥2.5×109/L; lymphocytes ≥1.0×109/L; hemoglobin≥9.0 g/dL or 5.59 mmol/L
9. Female patients with reproductive potential must agree to maintain highly effective methods of contraception by practicing abstinence or by using at least two methods of birth control from the date of consent through the end of the study. If abstinence could not be practiced, a combination of hormonal contraceptive (oral, injectable, or implants) and a barrier method (condom, diaphragm with a vaginal spermicidal agent) has to be used. Male patients must agree to use condoms during study participation.
10. Negative serum pregnancy test in women of childbearing potential.
11. Date of diagnosis of CML confirmed by laboratory PCR must be known.

9.3 Exclusion Criteria

1. Rare variants of BCR-ABL not quantifiable by RT-PCR according to the international scale (IS)
2. Current or previous autoimmune diseases requiring treatment
3. Immunosuppressive treatment of any kind
4. Prior allogeneic stem cell transplantation
5. Prior pegylated IFN therapy. Prior low dose conventional IFN treatment with ≤ 3 x 3 Mio I.E. / week for less than 1 year is acceptable
6. History of TKI resistance within the last 4 years of TKI therapy
7. History of accelerated phase or blast crisis
8. Hypersensitivity/allergy to the active substance or excipients of the formulation
9. Severe hepatic dysfunction or decompensated cirrhosis
10. Thyroid disease that cannot be controlled by conventional therapy
11. Epilepsy or other disorders of the central nervous system
12. Severe cardiac disease history including unstable or uncontrolled cardiac disease in the previous 6 months
13. Any history of retinopathy e.g. retinal detachment, degeneration or thromboembolic events
14. Clinically significant concomitant diseases or conditions, which, in the opinion of the investigator, would lead to an unacceptable risk for the patient to participate in the study (please refer also to the actual Investigator Brochure)

15. Other malignancy, except adequately treated superficial bladder cancer, basal or squamous cell carcinoma of the skin, or other cancer(s) for which the patient has been disease free for more than 3 years
16. Active or uncontrolled infections at the time of randomization
17. Pregnant and/or nursing women
18. Use of antibiotic therapy within the last 2 weeks prior to randomization
19. Concurrent use of molecular targeted therapy
20. Tested HIV sero-positivity or tested active hepatitis B or C infection
21. Participation in another clinical study with other investigational drugs within 14 days prior to randomization
22. Vaccination within 1 month prior to randomization
23. Any medical, mental, psychological or psychiatric condition (particularly severe depression, suicidal ideation or suicide attempt) that in the opinion of the investigator would not permit the patient to complete the study or comply to study procedures
24. Drug and/or alcohol abuse

9.4 Feasibility of recruitment

The study will be performed at about 20 centers in Germany. The participating CML-sites will be selected by the Coordinating Investigator and have adequate staff and experience in treating CML patients and in conducting clinical trials with adequate time, the targeted patient population and technical expertise to comply with the protocol. The study will be conducted within the German CML study group.

10 Study medication (IMP)

10.1 Characteristics of Investigational Medicinal Product (IMP)

Test Treatment: AOP2014 (PEG-Pro-IFNα-2b). Drug product is a clear colorless or slightly yellowish liquid solution.
Drug substance (P1101) is a mono-pegylated form of drug intermediate (P1040; Proline-IFNα-2b) produced in Escherichia coli cells by recombinant DNA technology. P1040 drug intermediate proline-interferon a-2b is a human interferon alpha-2b with an additional proline residue at its N-terminus that, in total, has 166 amino acids in length. P1040 is covalently modified with a 40 kilodalton (kDa) branched PEG-aldehyde. The pegylation site is predominantly at the N-terminal proline of P1040 drug intermediate. The IFNα-2b component of P1101 is the mature natural active form of the protein.

10.2 Potential Toxicity in Patients

The following safety data of AOP2014 refer to the current IB version (10.1).

Please note that data on adverse reactions/adverse events may change over the time. For that reason it is necessary that the investigator is familiar with the current version of the IB.

Adverse events related to AOP2014 treatment observed during the clinical trial program listed by system organ class and frequency (very common (≥ 1/10), common (≥ 1/100 to < 1/10), uncommon (≥ 1/1,000 to < 1/100), rare (≥ 1/10,000 to < 1/1,000), very rare (< 1/10,000) or not known (cannot be estimated from available data)

Infections and infestations	*common*: rhinitis<br>*uncommon*: upper respiratory tract infection, oral herpes, herpes zoster, oral candidiasis, sinusitis, oesophageal candidiasis, vulvovaginal mycotic infection, fungal skin infection, hordeolum, onychomycosis, body tinea
Blood an lymphatic system disorders	*very common*: leukopenia, thrombocytopenia<br>*common:* pancytopenia, neutropenia, anaemia
Immune system disorders	*uncommon*: basedow's disease
Endocrine disorders	*common*: hypothyroidism, thyroiditis<br>*uncommon*: hyperthyroidism
Metabolism and nutrition disorders	*common:* decreased appetite<br>*uncommon:* hypertriglyceridaemia
Psychiatric disorders	*common*: depression, insomnia, anxiety, mood altered, mood swings, listless<br>*uncommon*: acute stress disorder, hallucination, emotional distress, nervousness, apathy, nightmare, irritability<br>*not known*: suicide attempt, suicidal ideation, aggression, bipolar disorder, mania, confusional state<br>(Reported as adverse reactions during treatment with other interferon alpha products.)
Nervous system disorders	*common*: headache, dizziness, hypoesthesia, somnolence, paraesthesia<br>*uncommon*: peripheral motor neuropathy, radiculopathy, migraine, mental impairment, tremor, aura
Eye disorders	*common*: dry eye<br>*uncommon*: visual impairment, vision blurred, ocular discomfort<br>*not known*: retinopathy, retinal haemorrhage, retinal exudates, retinal artery occlusion, retinal vein occlusion, retinal detachment<br>(Reported as adverse reactions during treatment with other interferon alpha products.)
Ear and labyrinth disorders	*uncommon*: deafness, tinnitus, vertigo
Cardiac disorders	*uncommon*: atrial fibrillation, atrioventricular block, intracardiac thrombus, aortic valve incompetence, cardiovascular disorder
Vascular disorders	*common*: microangiopathy<br>*uncommon*: raynaud's phenomenon, umbilical haematoma, flushing
Respiratory, thoracic and mediastinal disorders	*common*: dyspnoea<br>*uncommon*: pneumonitis, cough, epistaxis, throat irritation<br>*not known*: lung infiltration, pneumonia, pulmonary arterial hypertension<br>(Reported as adverse reactions during treatment with other interferon alpha products.)
Gastrointestinal disorders	*common*: diarrhoea, nausea, abdominal pain, constipation, abdominal distension, dry mouth

	uncommon: gastritis, abdominal wall disorder, flatulence, frequent bowel movements, odynophagia, gingival bleeding
Hepatobiliary disorders	*common*: liver disorder, gamma-glutamyltransferase increased, alanine aminotransferase increased, aspartate aminotransferase increased, blood alkaline phosphatase increased<br>*uncommon*: hepatotoxicity, hepatomegaly
Skin and subcutaneous tissue disorder	*common*: pruritus, alopecia, rash, erythema, psoriasis, xeroderma, dermatitis acneiform, hyperkeratosis, hyperhidrosis<br>*uncommon*: photosensitivity reaction, skin exfoliation, nail dystrophy, dry skin
Musculoskeletal and connective tissue disorders	*very common*: arthralgia, myalgia<br>*common*: arthritis, pain in extremity, musculoskeletal pain, bone pain, muscle spasms<br>*uncommon*:, sjogren's syndrome, muscular weakness, neck pain, groin pain
Renal and urinary disorders	*uncommon:* cystitis haemorrhagic, dysuria, micturition urgency, urinary retention
Reproductive system and breast disorders	*uncommon:* erectile dysfunction
General disorders and administration site conditions	*very common:* influenza like illness, fatigue<br>*common:* pyrexia, injection site reaction, asthenia, chills, general physical health deterioration, injection site erythema<br>*uncommon*: injection site pain, injection site pruritus, sensitivity to weather change
Investigations	*common*: anti-thyroid antibody positive, blood thyroid stimulating hormone increased, body temperature increased, antinuclear antibody positive<br>*uncommon*: blood uric acid increased, blood lactate dehydrogenase increased, coombs test positive, weight decreased

Adverse events related to AOP2014 treatment (as listed in **IB Version 10.1, 21 March 2017, Table 5-12)**

Furthermore the Notification to investigators was published (dated 11 August 2017), listing the following new adverse events with possible relation:

- · Acute myocardial infarction
- · Urinary tract infection
- · Suicide

10.3 Selection of dose in the study

In the phase III PROUD-PV study (EUDRA-CT 2012-005259-18), AOP2014 is used at the starting dose of 100µg biweekly. This is equivalent to a pegIFN 2a dose of approximately 70 µg weekly. Previous clinical studies suggested that a dose of 45-90µg pegIFN weekly is therapeutically effective. At the lower dose of 45µg pegIFN weekly was associated with a well manageable toxicity [35,38,40]. **Therefore, a dose of 100µg AOP2014 to be administered biweekly was chosen as the standard dose for the ENDURE-CML study.**

To achieve a good tolerability of AOP2014 the study treatment will be escalated to full maintenance dose of 100µg as follows: 50 µg AOP2014 every 14 days for the first 4 weeks. From week 5 patients will receive full dose of 100µg once per two weeks.

10.3.1.1 Dose reductions and modifications

Dose modifications will be performed in the case of hematological or non-hematological side effects. A complete list of AOP2014 side effects can be found in the current “Investigator´s Brochure ” (see 10.6). There is limited experience with AOP2014 overdosage. The study, conducted in Austria (AOP Peginvera study) is an ongoing phase I/II study with the primary objective to identify the maximum tolerated dose (MTD) of AOP2014 in patients, diagnosed with polycythemia vera. Safety and tolerability is being assessed and an exploratory analysis of efficacy and biomarker modulation will be performed in this study. 51 patients were included into the study (recruitment closed). No DLTs were observed at the dose levels up to 540 µg every two weeks, and sound efficacy observations could be done. There is no specific antidote for pegIFN. Hemodialysis and peritoneal dialysis are not effective.

10.3.1.1.1 Dose modification in case of hematological toxicity

Table 3 displays the recommended AOP2014 dosage modifications due to neutropenia, or thrombocytopenia. Following improvement of the adverse reaction, neutropenia or thrombocytopenia, consider re-escalation of the dosage back to the previous dosage.

Laboratory Values	Recommended pegIFN Dosage
**Neutropenia**	
ANC < 750 cells/mm³	Reduce to 50 µg once every 14 days<br>Increase to standard dose once ANC count returns to >750 cells/mm³
ANC < 500 cells/mm³	Hold treatment until ANC values return to 1000 cells/mm³ or more. Reinstitute at 50 µg once every 14 days and monitor ANC.<br>Increase to standard dose once ANC count returns to >750 cells/mm³
**Thrombocytopenia**	
Platelet < 50,000 particles/mm³	Reduce to 50 µg once every 14 days
Platelet < 25,000 particles/mm³	Discontinue treatment until platelets return to 75.000 particles/mm³ or more. Reinstitute at 50 µg once every 14 days and monitor platelets.<br>Increase to standard dose once platelet count returns to >75.000 cells/mm³

Table 3 Recommended AOP2014 dosage modifications for neutropenia and thrombocytopenia

10.3.1.1.2 Dose modifications in case of non-hematological toxicity: depression

Depression Severity	Initial Depression Management (4-8 weeks)		Depression Management After 8 Weeks		
	Dosage Modification	Visit Schedule	Depression Severity Remains Stable	Depression Severity Improves	Depression Severity Worsens
Mild	No change	Evaluate once weekly by visit and/or phone	Continue weekly visit schedule	Resume normal visit schedule	Consider psychiatric consultation. Discontinue AOP2014 or reduce dosage to 50 µg every 14 days
Moderate	Decrease AOP2014 dosage to 50 µg once every 14 days	Evaluate once weekly (office visit at least every other week).	Consider psychiatric consultation. Continue reduced dosing	If symptoms improve and are stable for 4 weeks, you may resume normal visit schedule. Continue reduced dosage or return to normal dosage	Obtain immediate psychiatric consultation. Discontinue AOP2014 permanently.
Severe	Discontinue AOP2014 permanently	Obtain immediate psychiatric consultation.	Psychiatric therapy necessary.		

Table 4 Recommended dosage modification for depression management

10.3.1.1.3 Dose modifications in case of non-hematological toxicity: Liver toxicity

Following improvement of liver toxicity, re-escalation of the dosage back to the previous dosage should be warranted.

Laboratory Abnormality	Severity	Recommended AOP2014 dosing modification
Increased transaminase (ALT, AST)	For persistent or increasing elevations ≥5 but <10 x ULN	Modify dosage to 50 µg every 2 weeks
	For persistent ALT values ≥10 x ULN	Discontinue treatment

Table 5 Recommended Dose modification in case of liver toxicity

10.3.1.1.4 Dose modifications in case of other non-hematological toxicity

Severity	Recommended AOP2014 Dosage Modification
WHO I - II CTC	No dosage modification
WHO II CTC	Modify dosage to 50 µg every 2 weeks
WHO III CTC	Pause treatment until recovery to WHO I or II CTC
WHO IV CTC	Discontinue treatment

Table 6 Recommended Dose modifications in case of other non-hematological toxicities

For WHO CTC version and list please refer to the NCI CTC4.03.

10.4 Manufacturer of Study Medication

In brief, the drug substance production is performed as a four-stage process at 40 L scale:

(1) Fermentation and E. coli cell harvest,

(2) Isolation of P1040 drug intermediate (Proline-IFNα-2b) inclusion bodies,

(3) Refolding and purification of P1040 drug intermediate, and

(4) Pegylation, purification, and formulation of final Ropeginterferon alfa-2b drug substance (AOP2014)

The study medication will be manufactured by AOP Orphan Pharmaceuticals AG (Austria).

10.5 Handling and Storage of the Medication

Before injection, pen containing the AOP2014 should be kept at room temperature for 10 min. After cleaning the injection site with an alcohol swab, the correct dose of AOP2014 will be self-administered by the patient according to the instructions given by the treating study physician and the Instructions for Use provided with the pen. Self-administration will be documented in the e-CRF. A patient diary will be provided and shall help the patient to document AOP2014 injections, injected dose of AOP2014, side effects, and concomitant mediation, if necessary. Since a pre-filled auto-injection pen contains 0.5ml of AOP2014, equivalent to 250 µg active substance, each pen can be used for two injections using disposable needles that must be fixed to the pen before the infection. Thus, each pen contains sufficient substance for up to 4 weeks.

AOP2014 study medication must be stored until use in the fridge (2-8°C), in the outer carton in order to protect from light.
At the study center the refrigerator will be located in a locked room accessible only to authorized personnel. The temperature will be recorded on a temperature log.
Patients will be instructed to transport and store study medication at home, according to the storage requirements.

10.6 Reference Document

The current version of the AOP2014 Investigator´s Brochure (IB) is kept in the Investigator Site File (ISF). The AOP Orphan Pharmaceuticals AG is responsible for updates of the reference document and KKS Marburg for passing on the Coordinating Investigator, Ethics Committee, and Competent Authority and to the study centers. In case of changes in the IB the Coordinating Investigator has to re-evaluate the risk-benefit assessment of the study.

10.7 Labeling, Drug Supply and Accounting

CSM Clinical Supplies Management Europe GmbH will provide sites with the study medication at the request of KKS Marburg.

Study medication will be delivered to the study centers in the cooled containers. 1 pre-filled auto-injection pen with 0.5ml active AOP2014 substance and 2 injection needles will be packed together in the paper carton box. Pens and cartoon packages labels will comply with the legal requirements of Germany and will be printed in German. They will supply no information about the patient.

At the study center, the responsible designated site personnel have to confirm the receipt of study drug, by completing and faxing the enclosed form to CSM Clinical Supplies Management Europe GmbH (contact details on the form). The confirmation of receipt (in original) has to be filed in the ISF. The investigator must maintain an accurate record of the shipment in a drug accountability form. At the study center the study medication must be handled and stored safely and properly, and kept in a secured location to which only the investigator and designated site personnel have an access. Upon receipt it has to be stored according to the instructions specified on the labels.

Study medication will be dispensed at the investigator`s site by an authorized person. On each label designated site personnel has to fill in the adequate Pat.ID (first 2-digits will be a site number and next 3-digits will refer to sequencing number of the patient). Each patient will be provided with an instruction for use and an adequate supply for self-administration at home until their next scheduled visit. All study treatment dispensed will be recorded in a patient specific drug accounting log. Moreover, patients have to return all carton boxes and pens either containing unused study drug or the empty one, on a regular basis, at the end of the study, or at the time of study drug discontinuation. The Investigator or his/her designee will account for all study medication dispensed and returned.

At the conclusion of the study and, as appropriate during the course of the study, the Investigator will return all unused study pens, drug labels and a copy of the completed drug accountability forms.
Unless specifically instructed by KKS Marburg, beside from used pens the Investigator must not destroy any carton boxes, drug labels, or unused drug supply. Only after receiving written authorization by KKS Marburg, the Investigator will be instructed to either send all of the pens (containing unused study drugs) to the address provided at the time of authorization for destruction or to dispose of unused drug according to local regulations. Each disposal has to be documented and filed in the ISF.

All drug supplies are to be used only for this protocol and not for any other purpose.

10.8 Prior and Concomitant Therapy

Medication for concomitant diseases should be continued and will be documented in the e-CRF. Any experimental therapy is not allowed while on study. In addition, the TKI or immune-stimulatory (e.g. IFN) or immune-suppressive medication (e.g., steroids) are contra-indicated during the course of the study. However, musculoskeletal pain may occur in up to 30% of the patients within 1-6 weeks after TKI discontinuation. Symptoms then resemble rheumatic polymyalgia. They should be treated with paracetamol or non-steroidal anti-inflammatory drugs. Since corticosteroids may offset the effects of AOP2014, steroids should generally not be prescribed. Only in the rare cases that intolerable symptoms occur, steroids may be given at 10-20 mg prednisone p.o. and tapering within 2 weeks.
Patients cannot be vaccinated 1 month prior or during the period of study medication application.

11 Study medication (non-IMP)

11.1 Characteristics of non-Investigational Medicinal Product (non-IMP)

TKI is administered in the same way as before the start of the study for one more month.
Trade is used for this purpose.

11.2 Side effects caused by discontinuation of TKI therapy:

In approximately 20% of the patients, a "discontinuation syndrome" may occur after discontinuation of the TKI tablets. This means that the following symptoms may occur:

Bones and muscle pain, joint and tendon pain, joint inflammation (preferably the upper extremities of the shoulder girdle and the cervical spine)

The symptoms begin approximately 21 days after discontinuation of therapy and last up to months.

More frequently affected are patients who had already had bone joint and muscle problems earlier, or a particularly long TKI therapy.

In addition, a deterioration of an existing diabetes mellitus was observed and the occurrence of acne.

12 Study Procedures and Methods

12.1 Study Procedures

12.1.1 Screening Visit and Registration

CML patients having been treated with TKI for at least three years and being in at least MR^4 or better MR will be informed by the investigator about the purpose, risks and benefits of the ENDURE-CML study. Patients will obtain adequate time to consider participation in the study. The informed consent has to be signed at the clinic, during the Screening Visit at the latest and prior to any study-related assessment or procedure (see Table 1 and Table 2).
Eligible female patients with childbearing potential will continue to use highly effective methods of contraception as advised and standard for TKI treatment.

The Screening Visit includes:

- Obtain signed written informed consent before performing any study-related procedures[1]
- Verification of inclusion and exclusion criteria[1]
- Relevant demographics, Sokal, EUTOS, EURO and ELTS score
- Recording of medical and pre-treatment history
- Physical examination, vital signs and body temperature
- ECOG performance score
- BCR-ABL-PCR test at study reference laboratory in Jena or Mannheim
- Clinical Laboratory Evaluation
- Serum pregnancy test
- Serum HIV test
- Serum test for excluding active and chronic hepatitis B and C
- Autoimmunity (ANA, TSH) screen
- Assessment of concomitant medications and AEs

[1] If the patient will not meet all the inclusion criteria / fulfill the exclusion criteria, or will withdraw his informed consent before the randomization, he/she must be registered by KKS Marburg using the Screening/Randomization form (filled in the ISF).

12.1.2 Randomization and Baseline Visit (V1)

12.1.2.1 Randomization

When all of the inclusion criteria and none of the exclusion criteria are met, the randomization of an eligible patient can take place within the Baseline Visit (see 6).

12.1.2.2 Baseline Visit (V1) - Month 1

Time between Screening Visit and Baseline Visit should not exceed 2 months.

The Baseline Visit includes:

- Serum for immune parameters
- FACS and stem cell persistence
- IFNa prediction score
- TKI intake
- AOP2014 dosing and drug accountability (only arm A)
- Physical examination, vital signs and body temperature
- ECOG performance score
- BCR-ABL-PCR test at study reference laboratory in Jena or Mannheim

- Clinical Laboratory Evaluation
- Serum/urine pregnancy test
- Local tolerability assessment (only arm A)
- QoL
- Concomitant medications and AEs

Safety laboratory analysis should not be older than 7 days before start of study treatment with AOP2014 in Arm A. If the Baseline Visit (V1) will not be performed within 1 week from Screening Visit, the safety laboratory analyses assessments have to be repeated on V1 for arm A. This includes: complete blood counts including differential blood count (machine), Na, K, Ca, Krea, Urea, GOT, GPT, AP, GGT, LDH, Bilirubine, total Protein, PT, PTT, Glucose.

At Baseline Visit all of the patient will receive a patient diary. Moreover, patients who were randomized in arm A will be provided with enough pens to last until next scheduled visit.

Female patients are required to have a negative pregnancy test. Additional pregnancy urine tests should be given to woman with childbearing potential in arm A for using at home in case the time between visits exceeds 28 days. The investigator or study nurse has to explain the application of urine pregnancy tests.

12.1.3 Visit 2 – 12 (Month 2 – Month 13.5)

Patients will be seen according to the schedule outlined in study specific procedures for Arm A and B (Table 1 and Table 2). These tables list all necessary assessments and indicate with an “X” when they have to be performed. Each visit can be pre- or postponed by 7 days.

<u>**For patients randomized into arm A,**</u> treatment phase starts with the date of randomization and first AOP2014 injection. Patients randomized into arm A will injected **50 µg AOP2014 at day 0 (Baseline Visit) and 14 days thereafter. From month 2, patients will receive a full dose of 100 µg, every two weeks. First 100 µg dose has to be applied 2 weeks after the last application of 50 µg AOP2014.**

Importantly, only during the first month AOP2014 will be administered together with the TKI. After one month, the TKI will be stopped.

<u>**Patients randomized into arm B**</u>, will discontinue TKI one month after randomization. From this moment patient will receive no further treatment.

The following tests/evaluations have to be performed:

- Physical examination, vital signs and body temperature
- ECOG performance score
- BCR-ABL-PCR test at study reference laboratory in Jena or Mannheim
- Clinical Laboratory Evaluation
- Serum/urine pregnancy test
- Autoimmunity screen (<u>only arm A: Visit 2 and Visit 8)</u>
- Serum for immune parameters (<u>only Visit 8)</u>
- FACS and stem cell persistence (<u>only Visit 2 and Visit 8)</u>
- IFNa prediction score <u>(only Visit 2, Visit 8 and Visit 11</u>)
- AOP2014 dosing and drug accountability (<u>only arm A</u>)
- Local tolerability assessment (<u>only arm A</u>)
- QoL
- Concomitant treatments and AEs

Additionally, at each visit, patients (arm A) will be provided with enough pens to last until next scheduled visit.

12.1.4 End-of-Treatment assessment Visit 13 (Month 15)

A regular End-of-Treatment (EOT) assessment will be performed 15 months after randomization in **arms A and B** (procedures are outlined in Table 1 and Table 2).

Patients who relapse or otherwise discontinue the study treatment **prematurely,** before month 25**,** will also undergo an EOT visit prematurely; see 12.1.5. This visit should be scheduled closely to the date when the reason leading to treatment discontinuation (see 13) occurred, but latest at the date of the scheduled next visit. The further molecular BCR-ABL RT-PCR (IS) follow up will then be every three months until month 25 (12.1.6). A molecular and demographic/clinical Post Study Follow Up will be once every year for another 3 years (see study flow chart 1.1, Table 1 and Table 2 and 12.1.7).

IMPORTANTLY: In case of molecular relapse (loss of MMR) or withdrawal of informed consent TKI treatment must be resumed immediately in arms A or B, irrespective of the phase of study.

12.1.5 Premature End-of-Treatment assessments

Patients, who relapse **in arm A or arm B**, will resume TKI therapy and perform an early (premature) EOT visit. The molecular BCR-ABL RT-PCR (IS) follow up will then be performed and documented in the e-CRF (every 3 months after resuming the TKI as in surveillance phase until month 25 (see study flow chart 1.1 and Table 1 and Table 2).

12.1.6 Surveillance phase (month 15 to month 25)

In **arm A** surveillance phase refers to the time between month +15 to +25 (see study flow chart 1.1 and Table 1 and Table 2).

CAVE: the scheduled follow up differs slightly in arm A compared to arm B in that additional BCR-ABL RT-PCR (IS) assessments are required 16, 17, 19, 20, and 22.5 months after randomization (see study flow chart 1.1 and Table 1 and Table 2).

For patients randomized into **arm B,** surveillance phase refers to the entire time between month 0 to 25, because these patients never receive any study specific medication.

Patients, who relapse during surveillance phase **in arm A or arm B**, will resume TKI therapy and again perform an EOT visit; see 12.1.5. The molecular BCR-ABL RT-PCR (IS) follow up will then be performed and documented in the e-CRF (every 3 months after resuming the TKI until month 25 (see study flow chart 1.1 and Table 1 and Table 2).

12.1.7 Post Study Follow Up (PSFU)

Patients, who are in TFR 25 months after randomization, are recommended to undergo quantitative BCR-ABL RT-PCR (IS) monitoring every three months. However, an “off study” documentation of molecular remission will be required only once yearly for three years, namely 36, 48 and 60 months after randomization (see study flow chart 1.1 and Table 1 and Table 2). For all patients, the following data will be documented in PFSU 36, 48 and 60 months after randomization:

a) Survival (y/n)
b) Type of current treatment for CML
c) Demographic data including treatment history and relevant medical history (secondary malignancies, cardiac disease, auto-immune disease, progression of CML)
d) BCR-ABL RT-PCR (IS)

12.1.8 Study Schedule

- Recruitment period (months): 18
  Duration of study per patient (months): 25
- First patient in to last patient out (months): 43
- End of the study (months): after 49
  End of the study is defined as “Last Patient Last Visit and Database Closure”.
- Post Study Follow Up (months): 35

12.2 Study methods

12.2.1 Demography

Examined demographics include for example age and sex (see also 16.3).

12.2.2 EUTOS Score

The European Treatment and Outcome Study (EUTOS) risk score for chronic myeloid leukemia (CML) was derived from multivariate analysis of response of 2060 patients treated with imatinib for CML between 2002 and 2006. The score is applied at diagnosis, before starting therapy. The EUTOS score is calculated as (7 x basophil) + (4 x spleen), where “basophil” is basophils as a percentage of peripheral blood leukocytes and “spleen” is spleen size palpable below left costal margin [49].
The EUTOS score can be calculated online at: http://bloodref.com/myeloid/cml/eutos.

12.2.3 Euro and Sokal Scores

The Euro and Sokal scores [50,51] are calculated based on the following clinical and laboratory parameters that have to be assessed prior to CML –specific therapies including other than TKI.The Euro and Sokal scores can be calculated online at: **http://bloodref.com/myeloid/cml/sokal-hasford**.More information on the calculation of EUTOS, Euro, and Sokal score is available under:[52].

12.2.4 ELTS score

A recent score developed for long-term outcome of imatinib-treated patients is the EUTOS long-term survival score (ELTS score)[53]. As with the other three scores, score calculation should be based on clinical and laboratory parameters at diagnosis, prior to any CML-specific therapy including other than TKI. Its calculation can be performed via http://www.leukemia-net.org/content/leukemias/cml/elts_score/index_eng.html.

12.2.5 Medical and pre-treatment history

The medical and pre-treatment history will be assessed only once at Screening and will include: previous and ongoing clinically significant disorders.

12.2.6 Physical Examination / Vital Signs / Body Temperature

At each visit a complete physical examination will be performed. Moreover the blood pressure (systolic and diastolic), pulse rate and body temperature will be measured as scheduled in Table 1 and Table 2.

12.2.7 ECOG Performance Status

The performance status of the patients will be evaluated (Table 1 and Table 2) according to the ECOG performance status: ECOG = Eastern Cooperative Oncology Group.

ECOG Performance Status Criteria

Grade	Criteria and Description According to ECOG
0	Fully active, able to carry on all pre-disease performance without restriction
1	Restricted in physically strenuous activity but ambulatory and able to carry out work of a light or sedentary nature (e.g., light house work, office work)
2	Ambulatory and capable of all self-care but unable to carry out any work activities. Up and about more than 50% of waking hours
3	Capable of only limited self-care; confined to bed or chair more than 50% of waking hours
4	Completely disabled. Cannot carry on any self-care. Totally confined to bed or chair
5	Dead

Table 7 ECOG Performance Status Criteria

12.2.8 Clinical Laboratory Evaluation

Blood samples for safety evaluation (hematology, clinical chemistry, and coagulation parameters) will be collected as scheduled in Table 1 and Table 2. Determination of safety laboratory parameters will be performed at the central laboratories. Any abnormalities in any of the laboratory parameters will be judged in relation to the reference ranges from the laboratory and to the clinical relevance assessed by the investigator. The investigator will grade any clinically relevant parameter outside the normal range according to the NCI CTCAE (Version 4.03).

12.2.8.1 Hematology

Two (2) ml of blood will be placed in an ethylene diamine tetraacetic acid (EDTA) tube and the following parameters will be measured: hemoglobin, platelet count, leukocyte count, neutrophiles.

The clinical significance of each hematology value exceeding the alert values presented in Table 8 should be assessed and should be recorded in the e-CRF. When a value is clinically significant, an AE should be recorded.

Hematology Alert Values

Parameter	Alert Value
Neutrophil count	$\leq 1.0 \times 10^9$/L
Hemoglobin	≤ 9.0 g/dL or 5.59 mmol/L
Leukocyte count	$\leq 2.5 \times 10^9$/L
Platelet count	$\leq 100 \times 10^9$/L

Table 8 Hematology Alert Values

12.2.8.2 Coagulation

Five (5) ml of blood will be taken and placed in a citrate tube for determination of prothrombin time and activated partial thromboplastin time.

12.2.8.3 Clinical Chemistry

Nine (9) ml of blood will be taken and placed in a serum separation tube and the following parameters will be measured: ALT, AST, GGT, LDH, total bilirubin, AP, glucose, urea-N, creatinine kinase, creatinine, triglycerides, total protein, albumin, sodium, potassium, and calcium.
Additionally a serum pregnancy test will be performed every visit in female patients with childbearing potential and at Screening visit a serum test for Anti-HIV and a serum test for excluding active and chronic hepatitis B and C.

12.2.9 Autoimmunity

Four (4) ml of blood sample will be taken and placed in a serum separator tube for analysis of ANA and TSH.

12.2.10 BCR-ABL measurement and genetic studies

Because of the critical importance of a timely and accurate BCR-ABL quantitation, **it is important that all PCR measurements outlined in** Table 1 and Table 2 **will be performed in due time and only by the indicated reference laboratories** (see Study Personnel). After inclusion, starting with the screening procedure, BCR-ABL PCR assessments will be performed centrally at the EUTOS-certified Study Reference Laboratories for PCR (BCR-ABL mRNA) in Mannheim or Jena (see Study Personnel and Table 1 and Table 2).

The total amount of blood sampling depends on the respective visit (see 12.2.12). For BCR-ABL monitoring 20-30 ml of peripheral blood will be collected at every visit. This will occur by a non-risky peripheral venous puncture. Blood sampling for BCR-ABL monitoring is an obligatory procedure and critical to determine the primary endpoint. The blood will be sent directly to the BCR-ABL reference laboratory (see Study Personnel and Table 1 and Table 2). Here, the BCR-ABL mRNA level will be quantitated after isolation of total mRNA from peripheral blood cells. Unused RNA and cDNA will be stored in a pseudonymized manner using the Patient ID. Unused RNA or cDNA from patients who agreed to take part in the translational studies (by signing additional consent form) will be stored and can be used for subsequent PCR tests or for later independent research purposes.

12.2.11 Other Translational studies

At the Baseline Visit, at Visit 2, Visit 8, Visit 11 and at the EOT (Visit 13) and month 18 and 25, approximately 45 ml blood will be sampled for research purposes (see 12.2.12) if the patient has agreed to the scientific projects in a separate consent form. The donation of this additional amount of blood will not require an additional venous puncture.

Of special interest is to explore the value of 95 CD86+pDC / 10^5 lymphocytes at baseline (before TKI stop) as a predictor.

Serum (5 ml) will be analyzed to evaluate cytokine/chemokine profiles associated with response. The tubes will be sent by regular mail to the IMMUNE MARKER AND STEM CELL LABORATORY in Marburg (see Study Personnel and Table 1 and Table 2). Here it will be centrifuged for at least 10 minutes at about 1800 to 2000 × g. The serum will be collected, divided into 2 aliquots and stored at -80°C ± 10°C until analysis. Serum samples will be diluted and assayed with antibodies specific for the respective cytokines/chemokines following standard ELISA protocols. All samples will be measured against a standard curve. All standard and experimental samples will be analyzed in duplicate. Optical density will be read on a Packard Fusion reader and the resulting values analyzed against the standard curve value using the Fusion Reader Data Analysis software. Results will be expressed as pg/ml or ng/ml.

Moreover, Heparin blood (30 ml) will be for fluorescence activated cell sorting (FACS) will be collected in collection tubes as scheduled in the Table 1 and Table 2 (see also 12.2.12). The blood samples will be shipped at room temperature by regular mail to the IMMUNE MARKER AND STEM CELL LABORATORY at the University Hospital in Marburg (see Study Personnel and Table 1 and Table 2). The frequency and activation status of pDC, monocytes, B and T cells, and other immune cell populations will be evaluated. To determine the individual response of each patient to IFN stimulation *in vitro*, cytokine and chemokine production of PBMC will be analyzed. Therefore, PBMC may be also treated with IFN for 24 hours. Incubation time was optimized for the kinetics of the different parameters.

Finally, cells from the peripheral blood will be analyzed to assess whether the response to IFN and/or IFN-typical adverse events (depression, autoimmune phenomena) can be predicted from biomarkers measured before and initiation of IFN treatment and during treatment. Such predictive potential has

been demonstrated in patients with hepatitis C or malignant melanoma, who were treated with IFN [54-58]. For these studies, EDTA blood (9 to 10 ml) from the peripheral blood will be used and subjected to nucleic acid (RNA, DNA) and signal transduction (protein) analyses in the laboratory of Prof. Koschmieder at the University Hospital of Aachen (see Study Personnel and Table 1 and Table 2). Samples will be taken before and during IFN therapy from patients in the Arm A and also those in the comparator Arm B (to control for IFN effects).

The patient can participate in the study without donating blood for those accompanying translational projects. The biomaterial sampled during this study cannot be used for commercial purpose.

12.2.12 Total Blood Volume to be collected from Each Patient

The total amount of blood to be taken from each patient during the study cannot be calculated because the study duration will differ for each patient. For better overview the type and volume of blood sample has been summarized in this table (Table 9).

Type and Volume of Blood Sampling

Type of Sample (Including Screening Assessments)	Volume per Sample
**Safety**	
- Hematology	2 ml EDTA
- Coagulation	5 ml Citrate
- Clinical chemistry	9 ml Serum
- ANA, TSH	4 ml Serum
**Total**	**20 ml**
**Translational studies**	
- Serum immune parameters	5 ml Serum
- FACS immune profile	30 ml Heparin
- IFN prediction score	9-10 ml EDTA
**Total**	**44 ml**
**BCR-ABL PCR monitoring**	**20-30 ml EDTA**
**Total per Screening visit**	**~ 40 ml**
**Total per Baseline visit, V7 and EOT**	**~ 84 ml**
**Total per other visits**	**~ 36 ml**

Table 9 Type and Volume of Blood Sampling

12.2.13 Pregnancy test

A serum or urine pregnancy test will be performed at Screening Visit and at every visit (only arm A), for women who are considered of childbearing potential. Additional urine pregnancy tests will provided at baseline visit for usage at home in case the next study visit exceeds 28 days.

12.2.14 Local Tolerability

For patients randomized into arm A, local tolerability will be assessed. The following parameters will be evaluated:

- pain
- erythema
- swelling
- induration

12.2.15 Concomitant treatment

Drugs that are considered necessary for the patient's welfare may be given at the discretion of the Investigator. However, all of the concomitant medications need to be recorded in the e-CRF (see also Prior and Concomitant Therapy 10.8.).

12.2.16 Quality of Life Questionnaires

QoL will be assessed with the EORTC Quality of Life Questionnaire (QLQ-C30) version 3, the CML24 module.
QoL questionnaires must be filled out at the hospital/practice when the patient comes for a scheduled visit. The questionnaires will be handed out to the patients by the investigator or a study nurse prior to seeing the doctor for clinical evaluations. Patients will be asked to fill out the questionnaires as completely and accurately as possible. The average time to complete the entire questionnaire is approximately 10-15 minutes. QoL should be assessed at time-points indicated in 12.1 and Table 1 and Table 2.

12.2.17 Patient Diary

At Baseline Visit patients will receive a "patient diary form" to document the self-administration of study medication, toxicities/AEs and new concomitant medication. These sheets will be used by the treating doctor for documentation in the patient charts as data source for the e-CRF.

13 Discontinuation criteria

13.1 Withdrawal of patients from study treatment

All patients have the right to withdraw from the study at any time, for any reason, and without penalty or loss of benefits to which the patient is otherwise entitled. If the patient chooses to withdraw, the investigator must be informed immediately.

In case of premature end of the individual study therapy upon request of the patient, all efforts should be made to keep the patient in the study for follow-up. This is only possible if the subject will be informed verbally on the medically preferred option to still continue with the study surveillance procedures. The patient will then give his/her informed consent by means of a written, signed and dated consent form for the follow-up period. **The study visit(s) will then be continued according to protocol until the regular end of study (intention to treat).**

Reasons to prematurely discontinue study treatment in arm A or treatment free surveillance in comparator arm B **may** also be terminated by the investigator for one of the following reasons:

- Intolerable AEs / side effects of the study medication
- Severe (S)AE which make it necessary to stop study treatment permanently
- Abnormal laboratory value(s) which make it necessary to stop study treatment
- Non-compliance with the study protocol

Study treatment **must be** terminated for one of the following reasons:

- Pregnancy
- Reaching the primary endpoint, which is defined as an increase of the BCR-ABL level to > 0.1% (IS) at any time, corresponding to loss of MMR at any time
- Withdrawal of patient's consent to study treatment
- Study treatment termination by the investigator

In case of molecular relapse (loss of MMR) or withdrawal of informed consent TKI treatment must be resumed immediately in arms A or B, irrespective of the phase of study.

If the investigator terminates the treatment of patient prematurely, he has to inform the patient about his decision and has to record the primary reason for withdrawal in the patient file and to document the end of treatment in the e-CRF. Final study evaluations will be done according to the EOT visit. If the patient caused the premature withdrawal, all data before termination may be used for final analysis please also refer to 12.1.5 Premature End-of-Treatment assessments.

13.2 Premature discontinuation of the study

A possible premature discontinuation of a single center or of the study as a whole must be documented adequately with reasons being stated and information must have to be conveyed according to national requirements (e.g. EC, CA, local regulatory authorities).

13.2.1 Single center

The Coordinating Investigator together with KKS Marburg, the Competent Authority (CA; BfArM) and the Ethics Committee (EC) has the right to discontinue this study at any time for reasonable medical or administrative reasons in any single center. Possible reasons for termination of the study could be but are not limited to:

- Unsatisfactory enrolment with respect to quantity or quality
- Inaccurate or incomplete data collection
- Unexpected accumulation of SAE/AE
- Major failure to adhere to the study protocol

13.2.2 Study as a whole

The Coordinating Investigator together with KKS Marburg, the CA and the EC has the right to terminate this clinical study as a whole at any time for reasonable medical or administrative reasons. For example:

- Unexpected accumulation of SAE/AE
- Change of risk-benefit considerations

If the study is prematurely terminated or suspended for any reason, the Investigator/institution should promptly inform the EC, CA and study patient or patient's legal representative and should assure appropriate therapy and follow-up for the patients.

14 Safety

14.1 Definition

14.1.1 Adverse Events

An "adverse event" is defined as follows:

"Any untoward medical occurrence in a patient or clinical trial subject administered a medicinal product and which does not necessarily have a causal relationship with this treatment".1

"An adverse event can therefore be any unfavorable and unintended sign (including an abnormal laboratory finding, for example), symptom or disease temporally associated with the use of a medicinal product, whether or not considered related to the medicinal product".2

14.1.2 Adverse Reaction (AR)

An 'adverse reaction' is defined as follows:

[1] European Parliament and the Council, 2001: Directive 2001/20/EC. Article 2 (m).
[2] European Medicines Agency, 2006: ICH Topic E 2 A. Clinical Safety Data Management: Definitions and Standards for Expedited Reporting, Step 5. Section 2A.

'All untoward and unintended responses to an investigational medicinal product related to any dose administered'.[3]

'The definition implies a reasonable possibility of a causal relationship between the event and the IMP. This means that there are facts (evidence) or arguments to suggest a causal relationship'.[4]

14.1.3 Unexpected Adverse Reaction (UAR)

An unexpected 'adverse reaction' is defined as follows:

'An adverse reaction, the nature or severity of which is not consistent with the applicable product information (e.g. investigator’s brochure for an unauthorized investigational product or summary of product characteristics for an authorized product)'.[5]

14.1.4 Serious Adverse Event (SAE) or Serious Adverse Reaction (SAR)

A serious adverse event or serious adverse reaction is defined as follows:

'Any untoward medical occurrence or effect that at any dose:

- results in death,
- is life-threatening,
- requires hospitalization or prolongation of existing hospitalization,
- results in persistent or significant disability or incapacity,
- or is a congenital anomaly or birth defect'.[6]

'NOTE: The term "life-threatening" in the definition of "serious" refers to an event in which the patient was at risk of death at the time of the event; it does not refer to an event which hypothetically might have caused death if it were more severe'.[7]

The severity of an adverse reaction is largely determined by the outcome of the medical occurrence. An adverse reaction should only be termed "serious" if hospitalization did in fact take place as a result of it. As a rule, hospitalization is the admission to a hospital with at least one overnight stay.[8]

The presentation of a patient in the emergency room (casualty center, health care center) alone without subsequent in-patient admission does not yet fulfill the criterion hospitalization. However, it should be proofed whether any of the other criteria mentioned above justifies an adverse reaction being classified as "serious" or at least "medically significant.[9]

14.1.5 Suspected Unexpected Serious Adverse Reaction (SUSAR)

A SUSAR is an adverse event which

- has a reasonable possibility of a causal relationship to an IMP,
- is serious; and

---

[3] European Parliament and the Council, 2001: Directive 2001/20/EC. Article 2 (n).
[4] European Commission, 2011: Detailed guidance on the collection, verification and presentation of adverse event/reaction reports arising from clinical trials on medicinal products for human use ('CT-3'). Section 7.2.1, No. 45
[5] European Parliament and the Council, 2001: Directive 2001/20/EC. Article 2 (p).
[6] European Parliament and the Council, 2001: Directive 2001/20/EC. Article 2 (o).
[7] International Conference of Harmonisation, 2001: ICH Harmonised Tripartite Guideline Clinical Safety Data Management: Definitions and standards for expedited reporting - E2A: Section 2 B.
[8] Cf. Bundesinstitut für Arzneimittel und Medizinprodukte (BfArM) und Paul-Ehrlich-Instsitut und Bundesinstitut für Impfstoffe und biomedizinische Arzneimittel, 2010: 6. Bekanntmachung zur Anzeige von Nebenwirkungen und Arzneimittelmissbrauch nach § 63b Absatz 1 bis 9 des Arzneimittelgesetzes (AMG) vom 19.1.2010. Page 8.
[9] Cf. Bundesinstitut für Arzneimittel und Medizinprodukte (BfArM) und Paul-Ehrlich-Instsitut und Bundesinstitut für Impfstoffe und biomedizinische Arzneimittel, 2010: 6. Bekanntmachung zur Anzeige von Nebenwirkungen und Arzneimittelmissbrauch nach § 63b Absatz 1 bis 9 des Arzneimittelgesetzes (AMG) vom 19.1.2010. Page 8.

- is unexpected.[10]

Therefore a SUSAR is not consistent with the applicable product information (e.g. Investigator's Brochure (IB) for an unauthorized investigational medicinal product or Summary of Product Characteristics (SmPC) for an authorized medicinal product.

14.2 Documentation of Adverse Events

Adverse events have to be recorded on case report forms (CRF) from the time the informed consent has been signed, up until end of study (month 15) for arm B plus an additional safety visit for arm A (month 16).

14.3 Severity

Severity of AEs will be graded according to the 'National Cancer Institute - Common Terminology Criteria for Adverse Events' (CTCAE – Version-No.4.0).[11]

14.4 Causality

The assessment as to whether there is a reasonable possibility of a causal relationship (between the AE and the administration of study medication/TKI) is made by the reporting investigator according to following categories: [12]

Causality term	Assessment criteria
**Certain**	• Event or laboratory test abnormality, with plausible time relationship to drug intake<br>• Cannot be explained by disease or other drugs<br>• Response to withdrawal plausible (pharmacologically, pathologically)<br>• Event definitive pharmacologically or phenomenologically (i.e. an objective and specific medical disorder or a recognized pharmacological phenomenon)<br>• Rechallenge satisfactory, if necessary
**Probable**	• Event or laboratory test abnormality, with reasonable time relationship to drug intake<br>• Unlikely to be attributed to disease or other drugs<br>• Response to withdrawal clinically reasonable<br>• Rechallenge not required
**Possible**	• Event or laboratory test abnormality, with reasonable time relationship to drug intake<br>• Could also be explained by disease or other drugs<br>• Information on drug withdrawal may be lacking or unclear

[10] See European Commission, 2011: Detailed guidance on the collection, verification and presentation of adverse event/reaction reports arising from clinical trials on medicinal products for human use ('CT-3'). Section 7.3, No. 56.
[11] The term 'severity' is used here to describe the intensity of a specific event.
[12] Cf. Uppsala Monitoring Centre. The Use of the WHO-UMC system for standardised case causality assessment. Internet publication: http://who-umc.org/Graphics/24734.pdf (access 9th January 2013)

**Unlikely**	• Event or laboratory test abnormality, with a time to drug intake that makes a relationship improbable (but not impossible)<br>• Disease or other drugs provide plausible explanations
**Not related**	• No causal relationship
**Unassessable**	• Cannot be judged because information is insufficient or contradictory<br>• Data cannot be supplemented or verified<br>• Report suggesting an adverse reaction
**Not applicable**	• If any of causal relationship does not make sense (e. g. patient didn't receive any study medication prior to the start date of the event)

Table 10 Causality Assessment Criteria

14.4.1 Seriousness

The judgement as to whether the adverse event is serious is made by the reporting investigator.

14.4.2 Outcome

The outcome of adverse events has to be described by following criteria:

- recovered/resolved
- recovering/resolving
- not recovered/not resolved
- recovered/resolved with sequelae
- ongoing
- fatal
- unknown

Each adverse event has to be followed up:

- until the termination
- until improving is not to be expected
- until database is closed

14.5 Documentation and reporting of Serious Adverse Events

All serious adverse events (SAEs) have to be recorded on a study specific SAE form from the time the informed consent has been signed, until end of study (month 15) for arm B plus an additional safety visit for arm A (month 16). After that period of time, only serious adverse reactions (events related to study medication) have to be reported. The SAE form has to be completed in English. The Investigator must fax all serious adverse events (SAEs) **within 24 hours after awareness** of the event to:

KKS Marburg

Philipps-Universität Marburg

Karl-von-Frisch-Str. 4

D-35043 Marburg

FAX-No.: +49 (0)6421-28 66 559

The initial SAE report should be carried out by the investigator immediately, even if not all data are available.

The minimum information of an SAE report should include:

- Center-No.
- Patient-No.
- Name of reporting investigator
- Medical term of the SAE
- Name of study medication
- Causality assessment (relation between the reported event and the administration of study medication)

Furthermore, it is required for completing the SAE-Report:

- Start date of SAE
- Start date study medication
- Date of last dose (study medication) prior to SAE
- Seriousness criteria
- SAE outcome (at the time of the initial SAE report)
- Severity / Intensity of SAE

Relevant follow-up information must be faxed as soon as possible. SAE-Follow-Up reports also have to be recorded on the study specific SAE pages.

The medical term of the SAE should be an event, reaction or diagnosis rather than a list of symptoms. It is important to enter the most appropriate event term in the corresponding field. **Only one event term/diagnosis** should be entered on each SAE form. If more than one SAE is to be reported for the same patient, a separate SAE form for each SAE has to be completed.

In the case of death of a trial subject, the investigator has to provide any additional information necessary as requested by KKS Marburg (Sponsor), the competent authorities concerned and ethics committees concerned.

14.6 Exceptions from SAE-reporting

The following hospitalizations are not considered SAEs in this study:

Elective surgery or any other elective medical procedure that was planned prior or after signing consent.

14.7 Expectedness for IMP (Arm A)

KKS Safety management determines the expectedness of an adverse reaction according to the actual version of the Investigator´s Brochure (IB).[13]

- Expected event: already described in the ☐ SmPC ☒ IB
- Unexpected event: not described in the ☐ SmPC ☒ IB

14.8 Expectedness for Non-IMP

Not applicable.

14.9 Clinical assessment of relatedness for Non-IMP (both Arms)

The investigator will also evaluate the Non-IMP-relatedness after discontinuation of the medication.

[13] European Commission, 2011: Detailed guidance on the collection, verification and presentation of adverse event/reaction reports arising from clinical trials on medicinal products for human use ('CT-3'). Section 7.2.3.2, No. 51, 52.

14.10 Pregnancy

Pregnancies must be reported and followed up analogous to a SAE-report. In general, pregnant women should be excluded from clinical trials where the drug is not intended for use in pregnancy. If a patient becomes pregnant during administration of the drug, treatment should generally be discontinued if this can be done safely. It is the responsibility of the investigator to inform KKS pharmacovigilance **within 24 hours after awareness** of the pregnancy by using the study specific pregnancy reporting form (blank form is filed in ISF). Follow-up evaluation of the pregnancy, fetus, and child is very important and has to be reported on the study specific pregnancy reporting form. The pregnancy reporting form, follow-up evaluation and the pregnancy outcome report have to be stored in the ISF.[14]

14.11 Adverse events of special interest

- none

14.12 SUSAR reporting procedure

According to national legislation and European directives and guidelines, KKS Marburg (Sponsor) of the clinical trial has to report all SUSARs to the competent authority and ethic Committee in all Member States concerned and to the European clinical trials database (Eudravigilance Clinical Trial Module - EVTCM).

'The Sponsor shall ensure that all relevant information about suspected serious unexpected adverse reactions that are fatal or life-threatening is recorded and reported as soon as possible to the competent authorities in all the Member States concerned, and to the Ethics Committee, and in any case no later than seven days after knowledge by the Sponsor of such a case, and that relevant follow-up information is subsequently communicated within an additional eight days'.[15]

'All other suspected serious unexpected adverse reactions shall be reported to the competent authorities concerned and to the Ethics Committee concerned as soon as possible but within a maximum of 15 days of first knowledge by the Sponsor'.[16]

Furthermore it is the Sponsor's responsibility to inform all investigators.[17]

14.13 Safety-Manual

KKS Safety management performs the process of SAE-Assessment. In this context a study specific Safety-Manual has to be prepared. The Safety-Manual contains a detailed description of all procedures concerning the documentation and reporting of AEs, SAEs and SUSARs. Additionally the Safety Manual describes the preparation of the Development Safety Update Report (DSUR), the Benefit-Risk-Assessment and the process of immediate actions to prevent the trial subjects from immediate risks.[18, 19]

[14] European Medicines Agency, 2006: ICH Topic E8. General Considerations for Clinical Trials. Step 5, Section 3.1.4.3 a).
[15] European Commission, 2011: Detailed guidance on the collection, verification and presentation of adverse event/reaction reports arising from clinical trials on medicinal products for human use ('CT-3'). Section 7.1, No. 37.
[16] European Commission, 2011: Detailed guidance on the collection, verification and presentation of adverse event/reaction reports arising from clinical trials on medicinal products for human use ('CT-3'). Section 7.1, No. 37.
[17] See European Commission, 2011: Detailed guidance on the collection, verification and presentation of adverse event/reaction reports arising from clinical trials on medicinal products for human use ('CT-3'). Section 7.10, No. 108.
[18] See European Commission, 2011: Detailed guidance on the collection, verification and presentation of adverse event/reaction reports arising from clinical trials on medicinal products for human use ('CT-3'). Section 8, No. 124.
[19] See Bundesministerium für Justiz, 2012: GCP-Verordnung, § 11, §13 (4), §13 (5), §13 (6).

15 Data Management

15.1 EDC-System (e-CRF) and Data Management

The study will use an electronic case report form (e-CRF/EDC-System) for data collection and documentation, which is hosted by KKS Marburg. The data are entered directly via web browser to the e-CRF and are transferred via encryption (HTTPS (TSL/SSL)) to the central database.

Access to the e-CRF is only allowed for persons who are documented as trial personnel. Each person who is allowed to make entries in the e-CRF receives a personal username and the URL for database login upon request (User-ID request). The initial password, which has to be changed at first login, is transmitted automatically by email to the user upon request (Forgot Password?) to the personal email address, which is recorded in the system.

Before a user gets access to the productive environment, the user account is only activated for training. After the user has activated its account the user management at KKS enables the user for the appropriate site. The access level in the e-CRF depends on the group membership (investigator, study nurse, monitor, etc.). Thus, it is ensured that only authorized persons have access to the EDC system in order to document or monitor patient data. Users with monitoring function are not able to enter or change patient's data. They have the possibility to view the data write protected (review function) and they can use additional review functionality in case of any implausibility or questions/queries.

The completed e-CRF must be electronically signed (authorization) at the end of each visit by an investigator for each patient.

In order to ensure the anonymity of the patient data, the patient data in the e-CRF are recorded with a patient number consisting of a center number and a consecutive number. An allocation list (e.g. Rando-Log) containing the patient's patient number and the identifying data of the patient is only kept in the center.

Users of the EDC system receive training material (EDC Manual), which is provided by the KKS. Furthermore, on the homepage of the KKS Marburg under the heading Online Services (https://www.kks.uni-marburg.de/index.php/online-services) the prerequisites for using the system and further notes (FAQ). The EDC-Manual is part of the ISF and contains detailed instructions for using the EDC system. If necessary KKS Marburg will provide additional training material and required documentation for the users. For training purpose of data entry and data review a training site is included in the database.

In a multistage procedure, the given data will be checked electronically for their plausibility and consistency. Even during data collection, implausible data will be flagged automatically by implemented validation checks. Detected inconsistencies and missing or implausible data will be clarified with queries (electronically or paper-based) and necessary changes will be carried out.

The EDC system has an implemented audit trail. This assures that any documentation and/or changes to database items are traceable anytime.

At the end of study, the database will be closed after data cleaning process. This process will be documented according to SOPs of KKS Marburg.

The pseudonymized patient data recorded in the e-CRF are stored by the KKS Marburg in accordance with legal requirements.

16 Statistical Considerations

16.1 Hypotheses

Four hypotheses are tested in hierarchical order. To avoid inflation of type 1 error (false rejection of a null hypothesis), further confirmatory testing has to be stopped as soon as a null hypothesis could not be rejected. All four hypotheses are tested at significance level 0.05.

At first,

Null hypothesis 1: "The probability of molecular relapse-free survival (RFS) 7 months after randomization / 6 months after stopping TKI is not different between the two arms A and B"

is tested against the

Alternative hypothesis 1: "The probability of molecular RFS 7 months after randomization / 6 months after stopping TKI is different between the two arms A and B".

In case null hypothesis 1 can be rejected,

Null hypothesis 2: "The probability of molecular RFS 13 months after randomization / 12 months after stopping TKI is not different between the two arms A and B"

is tested against the

Alternative hypothesis 2: "The probability of molecular RFS 13 months after randomization / 12 months after stopping TKI is different between the two arms A and B".

In case null hypothesis 2 can be rejected,

Null hypothesis 3: "No difference in the molecular RFS probabilities between the two arms A and B at any time"

is tested against the

Alternative hypothesis 3: "Molecular RFS probabilities between the two arms A and B are different".

In case null hypothesis 3 can be rejected,

Null hypothesis 4: "The probability of molecular RFS 25 months after randomization / 24 months after stopping TKI is not different between the two arms A and B"

is tested against the

Alternative hypothesis 4: "The probability of molecular RFS 25 months after randomization / 24 months after stopping TKI is different between the two arms A and B".

16.2 Analysis Populations

Definitions of Analysis Populations

Intention-to-treat (ITT) population	All randomized patients
Safety population	All randomized patients who receive at least one dose of treatment in Arm A or attended at Visit 1 in Arm B. The safety population will be used for the analyses of all safety parameters.
Per-protocol (PP) population	All patients in the ITT population for whom no major protocol violations/deviations occurred

Table 11 Definitions of Analysis Populations

16.3 Demographic and Other Baseline Characteristics

All demographic characteristics, history of disease, CML baseline characteristics (BCR-ABL ratio,), prior treatment history (TKI treatment duration, type of TKI used, IFN therapy, prior unsuccessful discontinuation attempt), and CML characteristics (Sokal, EURO, EUTOS, and ELTS score at primary CML diagnosis, type of BCR-ABL transcript, cytogenetics at primary CML diagnosis) will be tabulated by treatment group and summary measures will be given for the intention-to-treat (ITT) population.

16.4 Efficacy Variables

The primary efficacy endpoint is molecular relapse-free survival (RFS).

Time to relapse is defined as the time from randomization to relapse (loss of MMR = increase of BCR-ABL ratio to > 0.1% according to the IS) or to death from any cause. Survivors without relapse will be censored on the last date they were known to be alive.

The secondary efficacy endpoints are:

- Treatment outcome correlation with predefined biomarkers (> or < 95 CD86$^+$pDC before TKI discontinuation, PD-1L, PD1 expression, PR1-CTL and cytokines in peripheral blood measured at date of randomization and at designated time point thereafter).
- Overall survival is defined as time from randomization to death from any cause. Survivors will be censored on the last date they were known to be alive.
- The QoL assessment in this study is planned to gain information on the QoL of CML patients under stopping conditions. The data will be compared between the treatment groups and to QoL of normal population. Furthermore, results of the CML24 Module should be shared with the EORTC group to complete the validation of this questionnaire.

16.5 Quality of life assessment

Primary objective:

- determine prospectively QoL of patients after stopping +/- IFN from randomization until EOS. This is a secondary endpoint and therefore mandatory.

Secondary objectives:

- to evaluate the impact of treatment discontinuation of QoL according to treatment arm
- to evaluate the impact of IFN therapy on QoL and to validate the CML24 module

The aim of QoL evaluation in this study is to get a better understanding of the effects of the treatment in terms of frequency and degree of related side effects from the perspective of the patients.

16.6 Safety Variables

The following safety assessments are considered as safety endpoints:

- Safety profile of AOP2014 in terms of the incidence of AEs graded according to NCI CTC Version 4.03;
- Autoimmunity induced by AOP2014 in terms of ANA.

16.7 Methods of Analysis

The analyses of the primary and secondary efficacy endpoints will be performed for the ITT population. All randomized patients will be analysed within the group they were randomized to, not regarding their actual further treatment. In addition, the confirmatory analysis of the primary endpoint will be performed for the PP population as sensitivity analysis.

Primary efficacy endpoint

The four hypotheses will be tested in hierarchical order, see above. Null hypotheses 1, 2 and 4 will be tested with the two-sided, uncorrected chi-square test. Null hypothesis 3 will be tested with the two-sided log-rank test. The result of BCR-ABL ratio according to the IS needs to be observed. In case of null hypotheses 1, 2, and 4, the BCR-ABL ratio according to the IS has to be available. For the times 6, 12, and 24 months after stop of TKI (i.e. 7, 13, and 25 months after randomization), a time interval around the corresponding time is allowed. Each result should be relatable to exactly one of the visits scheduled in the protocol. Accordingly, intervals are defined by the time in the middle between two visits (schedule of visits see e.g. study flow chart). As visits are scheduled 6, 7, and 8 months after randomization, the interval for relating a result to the 7-month visit is given by 6.5 to 7.5 months after randomization. With visits planned 11, 13, and 15 months, the interval for relating a result to the 13-month visit is given by 12 to 14 months, and finally, with visits foreseen 24, 25, and 36 months after randomization, the result should be recorded within the interval from 24.5 to 30.5 months after randomization. In case of more than one observation within an interval, the approach of Pfirrmann et al.59 will be chosen in order to determine the decisive result. In case of the observation of an extremely unfavourable result prior to the upper time limit of an interval, this result will be carried forward and will be rated as “no MMR/relapse”. Extremely unfavourable results are accelerated disease, blast crisis, or death from any course. Details on the outcome will be reported. For analyzing null hypothesis 3, it is important to ensure that the BCR-ABL ratio is evaluated at all times foreseen in Table 1. Accelerated disease and blast crisis imply a prior loss of MMR. Molecular RFS probabilities over time will be described by Kaplan-Meier estimates. RFS probabilities over time will be compared by the log-rank test.

Allogeneic stem cell transplantation in chronic phase or restart of TKI after prior stopping of TKI and without prior loss of MMR will be rated as a drop-out in case this happened prior to an evaluation which would be needed to determine the result for an interval corresponding to hypothesis 1, 2, or 4. In case of hypothesis 3, the observation time of a patient is censored at the time of allogeneic stem cell transplantation or of a restart of TKI without a prior loss of MMR. In this case, the censoring means a loss of information reducing the precision of the estimated probabilities of molecular relapse-free survival but should not alter the estimated probabilities themselves. Accordingly, it is important that the reasons for the reintroduction be clearly stated in any case. Estimated with the Kaplan-Meier method, the probabilities of molecular relapse-free survival describe what to expect, what can be really achieved, if the possibility of a premature TKI restart would not exist.

In case the event “restart of TKI without a prior loss of MMR” has to be regarded as informative with respect to the probabilities of molecular relapse-free survival, the Kaplan-Meier method is not

appropriate. Instead, the cumulative probabilities of a molecular relapse over time have to be estimated by the cumulative incidence function (CIF) 60. The event “restart of TKI without a prior loss of MMR” is then regarded as a competing risk.

Whatever the situation, non-informative or informative censoring, the cumulative incidence function will be calculated (in addition). It will present the cumulative probabilities of a molecular relapse under consideration of the presence of the competing event “restart of TKI without a prior loss of MMR” and also provide an estimation of the probabilities of this competing event itself.

Differences between the treatment strategies prior to discontinuation will be tested by the Gray test (Gray RJ et al., Ann Stat, 1988), if the assumption of non-informative censoring does not hold.

As explorative analyses, depending on the endpoint, multivariate logistic or Cox regression analyses will be performed to analyse the influence of baseline covariates such as the effect of different duration of prior TKI therapy.

Secondary efficacy endpoints
All analyses of secondary efficacy endpoints will be performed by appropriate descriptive statistics.

The value of $CD86^{+}$pDC / 10^{5} lymphocytes at Screening Visit as a predictor of RFS will be tested within a Cox model. As treatment group (A or B) is assumed to be associated with RFS, for validation, the Cox model will be stratified for treatment group to remove its influence. However, to also judge the prognostic value of $CD86^{+}$pDC additional to treatment group, treatment group and a possible interaction term between both factors will be considered together in an unstratified Cox model.

Apart from attempting validation of the prognostic influence for CD86+pDC / 105 lymphocytes, the prognostic influence of further clinical, immunological, and genetic biomarkers on relapse-free survival is investigated. To perform these explorative analyses, candidate variables are considered as part of a multivariate Cox model stratified for treatment.

For overall survival the same analyses as for RFS over time will be performed. Whether multivariate analyses are possible depends on the number of events.

All secondary endpoints will be evaluated at the final analysis which is performed after the 25-month follow-up for all randomized patients was collected.

Safety endpoints
The safety analyses will be based on the safety population. Safety variables will be tabulated by treatment group and listings will be given.

Cumulative probabilities of the occurrence of adverse events will be calculated. Firstly, cumulative probabilities for the occurrence of any adverse event, secondly the occurrence of any adverse event of grade 3 or 4 will be plotted by cumulative incidence curves. Cumulative incidence curves will consider competing risks. One competing risk would be death without prior recording of any adverse event.

Using cumulative incidence curves, also probabilities for the observation of particular adverse events of interest are considered.

16.8 Interim Analyses

Two interim analyses are planned. Firstly, after the BCR-ABL/ABL ratio 7 months after randomization is available for all patients still under study and secondly, after the BCR-ABL/ABL ratio 13 months after randomization is available for all patients still under study. Accordingly, the first and second hypotheses will be tested, respectively. At the final analyses, hypotheses 3 and 4 will be tested.

16.9 Analyses of “off study follow-up”

Molecular relapse-free survival (RFS) will be reported at the three fixed times, 36, 48, and 60 months after randomization. Again, at the final post study follow-up analysis, molecular RFS will also be analysed as a time-to-event variable.

Secondary endpoints

As before, overall survival probabilities will also be estimated at the final post study follow-up analysis.

The type of current treatment for CML will be described.

Demographic data including treatment history and relevant medical history (secondary malignancies, cardiac disease, auto-immune disease, progression of CML) will be compared between arm A and B in a descriptive manner.

16.10 Sample Size Calculation

Molecular relapse-free survival (RFS) will be tested at three fixed times, 7, 13, and 25 months after randomization. In arm B, without any treatment after stopping TKI, the probabilities of molecular RFS at 7, 13, and 25 months are assumed to be 55%, 50%, and 45%. At each time, an increase by an absolute value of 20% in arm A is expected. At all three times, confirmatory testing is intended. To avoid an inflation of type I error, the hypotheses are put in hierarchical order. At all times, a power of 80% is planned. It is a characteristic of the binomial distribution that the variance is the higher, the nearer the probabilities are to the level of 50%. Accordingly, sample size estimation needs to be based on the assumptions at 25 months.

Patients in the standard arm B are expected to have a molecular relapse-free survival probability of 45% 24 months after discontinuation of TKI treatment. In the investigational arm A, we expect that the molecular RFS probability after 24 months of discontinuation of TKI can be increased by 20% as compared with the standard arm. Accordingly, 192 patients need to be randomized 1:1 to be able to reject the null hypothesis of same relapse-free survival probabilities with a two-sided uncorrected chi-square test at a significance level of 0.05 and a power of 80%.With an expected drop-out of 10%, the 1:1 randomization of 214 patients is necessary. The sample size calculation was performed with the program “PS Power and Sample Size Calculations", Version 3.1.2 (Dupont WD et al., Controlled Clin Trials, 1997).

The hierarchical ordering of the hypotheses will be in accordance with the chronological ordering of the times. Assuming that all drop-outs will occur before 6 months (the most adverse case), 192 patients would be ready for analysis. With these 192 patients randomized 1:1, a molecular RFS probability of 55% at 7 months in arm B and of 75% in arm A, we would be able to reject the null hypothesis of same relapse-free survival probabilities with a two-sided uncorrected chi-square test at a significance level of 0.05 with a power of 83%.

With 192 patients randomized 1:1, a molecular RFS probability of 50% at 13 months in arm B and of 70% in arm A, we would be able to reject the null hypothesis of same relapse-free survival probabilities with a two-sided uncorrected chi-square test at a significance level of 0.05 with a power of 81%.

Apart from testing three hypotheses at fixed times, molecular RFS probabilities are investigated over time. Accordingly, molecular RFS will also be analyzed as a time-to-event variable. Assuming exponential survival times, an RFS probability of 45% at 25 months corresponds to a hazard of 0.0333 and a median survival time of 20.82 months. With an RFS probability of 65%, the hazard in the experimental arm A is 0.0179 and the hazard ratio of arm B to arm A is 1.8603. Of course, the assumption of exponential distributions does not fit – we will rather have more molecular relapses earlier, meaning higher hazards in both arms. Thus, if we assess power on the assumption of

exponential distributions, we will most likely end up with an estimation of a minimum power to be expected. It is further assumed that testing will be performed only after all patients had their minimum follow-up of 25 months. Again, being cautious in avoiding overestimation of power, accrual time was set to 0 months – this in accordance with the assumption that hardly any molecular relapse will occur after 25 months. Under all these assumptions and with 192 evaluable patients randomized 1:1, the power to reject the hypothesis of same molecular RFS probabilities with the log-rank test will be (slightly above) 80%. Again, sample size calculation was performed with the program “PS Power and Sample Size Calculations", Version 3.1.2

16.11 General principles of statistical analyses

As for testing the four confirmatory hypotheses, for explorative testing, the level of significance will be 0.05. It will be stated that all analyses apart from the four pre-planned hypotheses is explorative. Point estimations will be given together with their 95% confidence interval. In general, the software for analysis will be SAS version 9.4 or a later distributed version. Only for statistics which will not be available in SAS, the open source programming language R will be used.

17 Administration

17.1 Source Data and Patient Files

The investigator has to keep a written or electronic patient file for every patient participating in the clinical study. In this file, the available demographic and medical information of a patient has to be documented, in particular the following: name, date of birth, sex, height, weight, patient history, concomitant diseases and concomitant drug (including changes during the study), statement of entry into the study, study identification, patient number, the date and process of informed consent, all study visit dates, predefined performed examinations and clinical findings, observed AEs (if applicable), and reason for withdrawal from the study if applicable. It should be possible to verify the inclusion and exclusion criteria for the study from the available data in this file. It must be possible to identify each patient by using this patient file.

Additionally, any other documents with source data, especially original printouts of data that were generated by technical equipment have to be filed. All these documents have to bear at least patient identification and the printing date printed by the recording device to indicate to which patient and to which study procedure the document belongs. The medical evaluation of such records should be documented as necessary and signed/dated by the investigator.
Computerized patient files will be printed whenever source data verification is performed by the monitor. Printouts must be signed and dated by the investigator, countersigned by the monitor and kept in a safe place.

For the current study, documents considered to be source data include (but are not limited to):

- Patient’s record (patient’s clinic and/or office chart, hospital chart)
- Patient Informed Consent Form (PIC)
- Laboratory results
- Treatment notes
- Questionnaires
- Any other records maintained to conduct and evaluate the clinical study

17.2 Data Safety and Monitoring Committee (DSMC)

During the study, safety data will be monitored by an independent Data Safety Monitoring Committee (DSMC) consisting of 3 independent external experts in hematology/oncology, and immunology/toxicology, and a biostatistician. On behalf of the study team, the Coordinating Investigator, KKS Marburg representatives, and additional experts may be invited to participate in the open part of the regular meetings.

KKS Marburg will convene DSMC meetings as telephone conferences if possible.

The DSMC will review the conduct of the trial regarding accrual, protocol compliance, and general safety issues (clinical safety, tolerability and laboratory data). The DSMC may also review AEs for both treatment arms, develop recommendations, and take votes as necessary.

Based on these data, the DSMC will recommend if the study can be continued according to the procedures described in this study protocol. No communication, either written or oral, of the deliberations or recommendations of the DSMC will be made outside of the DSMC except as provided for in this description. Each member of the DSMC must sign a corresponding statement.

17.3 Monitoring, Audit and Inspection

The investigator will permit study-related monitoring and audits, Ethics Committee review, and regulatory inspections, providing direct access to source data/documents.

17.3.1 Monitoring

The monitoring of the study takes place by the trained staff of the Schantl Pharma Service GmbH. Patient recruitment can begin after the initiation visit. During the course of the study each participating center will be visited for monitoring before, during and after the trial. The scope, frequency and depth of the monitoring will be specified in a study specific monitoring manual.
During each of these visits, source data verification will be performed on the basis of a pre-specified sampling plan, generated by KKS Marburg.
In general any discrepancies in the e-CRF should be discussed and clarified with the study team during the monitoring visit and corrections/additions should be done according to GCP requirements. Furthermore, at these visits problems will be discussed. Source data verification will be performed by direct access to the original patient records. KKS Marburg guarantees that patient confidentiality will be respected at all times. Participation in this study will be taken as agreement to permit direct source data verification.

17.3.2 Audit and Inspections

In compliance with European regulations/ICH-GCP Guidelines, it is required that the investigator and institution permit authorized representatives of KKS Marburg and the regulatory agency(ies) direct access to review any study-related documents and subject's original medical records for verification of study-related procedures and data during and/or after the study. The extent is permitted by the applicable laws and regulations and that, by signing a written informed consent form, the subject or the subject's legally acceptable representative is authorizing such access.
Direct access includes examining, analyzing, verifying, and reproducing any records and reports that are important to the evaluation of the study. The investigator is responsible for giving any requested support for any monitoring, inspection or audit visit. The Principal Investigator has to be available during these visits.

17.4 Investigator Site File (ISF) and archiving

The investigator will obtain an Investigator Site File (ISF) before start of the study from KKS Marburg. This file contains all relevant documents necessary for the conduct of the study. This file and associated study-related documents must be safely archived after termination of the study for at least 10 years. It is the responsibility of the investigator to ensure that the patient identification list is stored

for at least 15 years beyond the end of the clinical study. All original patient files must be stored for the longest possible time permitted by the regulations at the hospital, research institute, or practice in question. If archiving can no longer be maintained at the site, the investigator will notify KKS Marburg.

17.5 Protocol violation and discrepancies

Any protocol violation has to be recorded and documented either as "note" in the e-CRF or by using the study specific File Notification Form, which has to be signed and forwarded to KKS Marburg.
In KKS Marburg all protocol violation will be tracked in a separate database. A continuous evaluation of all protocol violation encountered throughout the study will be done by the Coordinating Investigator and KKS Marburg and if needed with the responsible statistician latest at times of planned data analysis.

18 Ethical Considerations

Good Clinical Practice (GCP) is an international ethical and scientific quality standard for designing, conducting, recording, and reporting studies that involve the participation of human patients. The study will be conducted in compliance with GCP and the applicable national regulations to assure that the rights, safety, and well-being of the participating patients are protected consistent with the ethical principles that have their origin in the Declaration of Helsinki.

19 Ethical and regulatory aspects

19.1 Investigator's responsibilities

The Principal Investigator at site has adequate experience in the conduct of clinical trials and designates a deputy with comparable qualification before the start of the study. He/she has to lead and supervise the trial site team set up for this study (study team), which consists of qualified personnel in the field of the study specific indication and GCP. The principal investigator is also obliged to forward any study specific information (e.g. study protocol, product information) or updates of these documents to the study team. He/she is responsible to conduct the clinical study in accordance with the protocol, the ethical principles that have their origin in the Declaration of Helsinki (current version) as well as in accordance with the International Conference on Harmonization of Technical Requirements of Pharmaceuticals for Humans Use (ICH) Guideline for Good Clinical Practice E6 (current version), and the relevant applicable national laws and regulatory requirements.
Each subinvestigator is responsible conduct the designated duties in accordance with the protocol, especially concerning treatment of the patients, the AE/SAE announcement to KKS Marburg and related e-CRF documentation.

The Coordinating Investigator is responsible for setting up the DSUR in cooperation with the statistician and the safety manager and may be contacted for medical questions. He is also responsible for archiving study-relevant documents and data according to national regulation.

19.2 Further responsibilities

KKS Marburg is responsible for project management (incl. regulatory affairs such as ethics and authority approval and the registration of the study), safety management and data management.
Schantl Pharma Service GmbH is responsible for monitoring.
Further responsibilities will be specified in separate contracts between the participating parties.

19.3 Patient information and consent

The consent of the patient/subject or professional legal representative to participate in the clinical study has to be given in writing before any study-related activities are carried out. A subject information sheet will be provided for the purpose of obtaining informed consent. It must be signed

and personally dated by the subject after giving the patient a reasonable time for decision. After that by the investigator designated by the Principal Investigator who to conduct the informed consent process should also sign and date personally the patient consent form. It will be revised and forwarded to the patients whenever important new information becomes available that may be relevant to the consent of subjects. At the study center the investigator or his designate will inform the patient/subject or professional legal representative verbally about all risks, the aim of the study, all study procedures and possible alternatives. In doing so, the wording used will be chosen so that the information can be fully and readily understood by laypersons.

By signing informed consent patient will also agree on blood sampling, to perform BCR-ABL PCR assessments in peripheral blood cells (12.2.10). Since it is critical to determine the primary endpoint, this procedure is obligatory for each patient willing to take a part in the ENDURE-CML study.

Provision of consent will be confirmed in the patient file by the investigator. The signed and dated declaration of informed consent will remain in the ISF and can be retrieved at any time for monitoring, auditing and inspection purposes. A copy of the signed and dated information consent together with the patients insurance should be provided to the subject prior to participation.
Only physicians may inform the patients and obtain the consent for the trial.

In addition, the investigator will inform and ask the patient for the permission of optional blood sampling (at Baseline Visit, Visit 2, Visit 8, Visit 11, EOT Visit and in the Surveillance Phase month 18 and month 25). The biomaterial would be used for the accompanying translational projects (12.2.11). To perform this procedure patient has to agree separately in the informed consent form. Patient’s disagreement does not affect their participation in the ENDURE-CML study.

In case of premature end of the individual study therapy upon request of the patient, the subject will be informed verbally on the possibilities to continue the study procedures and gives informed consent by means of a written, signed and dated consent form for the follow-up period. Only an investigator may obtain the consent for the follow-up in accordance with the regulatory requirements and GCP principles.

19.4 Patient insurance

For all patients in this trial, an insurance covering possible harm to the patients is contracted at the HDI Global SE.

HDI Global SE
Niederlassung Düsseldorf
Am Schönenkamp 45
40599 Düsseldorf
Insurance number: 5701031203019

This insurance covers any damage to health arising from participation in the study up to a maximum sum. In order not to violate the insurance cover, the patient must immediately notify the insurance company or the investigator in case of any damage to health arising from participation in the clinical study. A copy of the complete insurance terms and conditions will be made available to the patient with a copy of the signed patient information and consent form.

19.5 Ethics Committee (EC) or Institutional Review Board

KKS Marburg certifies that written documentation of appropriate Ethics Committee approval of the protocol, patient information and informed consent will be obtained before the beginning of this study.

The investigator will not begin the study until he has received written confirmation of approval by the EC and notification to local authority (see 19.6.2). KKS Marburg has to inform the leading EC within 90 days after termination of the clinical trial (in case of suspending or interruption within 15 days).

Prior to commencement of the study, the study protocol will be submitted together with its associated documents (patient information, consent form, product information) to the relevant EC for their favorable opinion.

The study will only commence following provision of a written favorable opinion, documenting the date of the meeting, constitution of the committee and voting members present at the meeting as well as clearly identifying the trial, protocol version, and consent documents reviewed.

Any substantial amendments to the protocol will be submitted to the EC and they will be informed about SUSARs in accordance with national requirements. Additional trial sites may only recruit patients, if KKS Marburg already obtained approval for the site. Where the clinical trial has been suspended or interrupted by KKS Marburg, KKS Marburg informs the Ethics Committee, giving the reasons for suspension or interruption.

19.6 Regulatory Authorities

19.6.1 Notification to competent authority

Prior to commencement of the study, the study protocol will be submitted together with its associated documents to the Competent Authority (BfArM) for their favorable opinion. According to the German Drug Law (AMG) the study will only commence following provision of a written favorable opinion by the Competent Authority.

KKS Marburg has to inform the competent authority before the beginning and within 90 days after termination of the clinical trial (in case of suspending or interruption within 15 days giving the reasons for suspension or interruption).

19.6.2 Notification to the local competent authority

According to §67 AMG the Principal Investigator shall inform the local authority about start and termination of the clinical trial.

Where the clinical trial has been suspended or interrupted by KKS Marburg, KKS Marburg informs the Competent Authority (BfArM), giving the reasons for suspension or interruption. The investigator has to inform the local authority about this.

The principal investigator and deputy principal investigator can delegate these responsibilities to KKS Marburg. This must be documented and signed by each investigator.

19.7 Changes to Study Protocol

'After the commencement of the clinical trial, the Sponsor may make amendments to the protocol. If those amendments are substantial and are likely to have an impact on the safety of the trial subjects or to change the interpretation of the scientific documents in support of the conduct of the trial, or if they are otherwise significant, the Sponsor shall notify the competent authorities and shall inform the ethics committee'.[20]

Also the investigator must inform the local authority about any substantial amendments and changes of the principal investigator and the deputy principal investigator.

'If the opinion of the Ethics Committee is favorable and the competent authorities of the Member States have raised no grounds for non-acceptance of the abovementioned substantial amendments, the Sponsor shall proceed to conduct the clinical trial following the amended protocol'. [16]

Changes of administrative or technical nature will be recorded in a document entitled “non-substantial amendment“. It will be sent for information to the relevant ECs and to the CA.

[20] DIRECTIVE 2001/20/EC OF THE EUROPEAN PARLIAMENT AND OF THE COUNCIL of 2 April 2001, Article 10 (a)

All signatories must sign amendments of the protocol. All investigators will acknowledge the receipt and confirm by their signature on the Amendment that they will adhere to the amendment. A copy of the signature page will be filed in the Investigator Study File the original in the Trial Master File.

19.8 Safety of subjects, immediate danger

'In the light of the circumstances, notably the occurrence of any new event relating to the conduct of the trial or the development of the investigational medicinal product where that new event is likely to affect the safety of the subjects, the Sponsor and the investigator shall take appropriate urgent safety measures to protect the subjects against any immediate hazard.
The Sponsor shall forthwith inform the competent authorities of those new events and the measures taken and shall ensure that the Ethics Committee is notified at the same time'.[21]

Other administrative changes (not affecting the scope of the investigation or the scientific quality of the study) can be made following approval by the principal investigator; however the EC must be notified of these administrative protocol changes (as appropriate).
The principal investigator will be responsible for implementing any amendments at the study site (including the distribution of amendments to all staff concerned).

19.9 Pre-conditions before study start

Before study start and recruitment of patients following conditions have to be fulfilled:

- Favorable opinion to the study of the Ethics Committee (including investigators und deputy investigators) and of the Competent Authority (BfArM)
- Information to the Competent Authority (BfArM) about the start of the study
- Notification of KKS Marburg to the local authorities about study start according to §67 AMG
- Notification of all investigators and deputy investigators according to §67 AMG
- Signed study contract
- Signed study protocol (by principal investigator, deputy principal investigator and all subinvestigators)
- Initiation Visit

20 Financial Aspects

The financial aspects of the study will be documented in separate agreements between KKS Marburg and the study centers. The study is supported by a research grant from the Deutsche Krebshilfe.

21 Final Report

The final report will be set up as synopsis according to ICH E3 and will be submitted to the EC and CA within one year after termination of the study. The KKS Marburg together with the Coordinating Investigator and the responsible statistician are responsible for the preparation of the report and submission to the EC and CA.

[21] DIRECTIVE 2001/20/EC OF THE EUROPEAN PARLIAMENT AND OF THE COUNCIL of 2 April 2001, Article 10 (b)

22 Registration

The study will be registered in an official WHO-accepted register before the first patient will be recruited. KKS Marburg is responsible for the first registration and following updates.

23 Publication

In this multi-center study, the main publication will be a full publication of all data from all sites. Any publications of the results, either in part or in total (abstracts in journals, oral presentations, etc.) by Investigators or their representatives will require pre-submission-review by KKS Marburg and the Principal Investigator / Steering Committee. The Principal Investigator will be given the choice to be the first or the last author for the main publication. The remaining positions will be based on recruitment, good data quality and scientific input to the study. The final author list will be a joint agreement by the Principal Investigator, the Steering Committee and KKS Marburg. For all other publications, the order of the authors will be determined according to recruitment, data quality and significant scientific input to the study, after consultation with the Steering Committee.

24 References

1. Ben-Neriah Y, Daley GQ, Mes-Masson AM, Witte ON, Baltimore D. The chronic myelogenous leukemia-specific P210 protein is the product of the bcr/abl hybrid gene. *Science*. 1986;233(4760):212–214.
2. Daley GQ, Van Etten RA, Baltimore D. Induction of chronic myelogenous leukemia in mice by the P210bcr/abl gene of the Philadelphia chromosome. *Science*. 1990;247(4944):824–830.
3. Perrotti D, Jamieson C, Goldman J, Skorski T. Chronic myeloid leukemia: mechanisms of blastic transformation. *J. Clin. Invest.* 2010;120(7):2254–2264.
4. Saussele S, Lauseker M, Gratwohl A, et al. Allogeneic hematopoietic stem cell transplantation (allo SCT) for chronic myeloid leukemia in the imatinib era: evaluation of its impact within a subgroup of the randomized German CML Study IV. *Blood*. 2010;115(10):1880–1885.
5. Hochhaus A, O'Brien SG, Guilhot F, et al. Six-year follow-up of patients receiving imatinib for the first-line treatment of chronic myeloid leukemia. *Leukemia*. 2009;23(6):1054–1061.
6. Larson RA, Hochhaus A, Hughes TP, et al. Nilotinib vs imatinib in patients with newly diagnosed Philadelphia chromosome-positive chronic myeloid leukemia in chronic phase: ENESTnd 3-year follow-up. *Leukemia*. 2012;26(10):2197–2203.
7. Jabbour E, Kantarjian HM, Saglio G, et al. Early response with dasatinib or imatinib in chronic myeloid leukemia: 3-year follow-up from a randomized phase 3 trial (DASISION). *Blood*. 2014;123(4):494–500.
8. Baccarani M, Deininger MW, Rosti G, et al. European LeukemiaNet recommendations for the management of chronic myeloid leukemia: 2013. *Blood*. 2013;122(6):872–884.
9. Cross NCP, White HE, Müller MC, Saglio G, Hochhaus A. Standardized definitions of molecular response in chronic myeloid leukemia. *Leukemia*. 2012;26(10):2172–2175.
10. Müller MC, Cross NCP, Erben P, et al. Harmonization of molecular monitoring of CML therapy in Europe. *Leukemia*. 2009;23(11):1957–1963.
11. Hehlmann R, Müller MC, Lauseker M, et al. Deep Molecular Response Is Reached by the Majority of Patients Treated With Imatinib, Predicts Survival, and Is Achieved More Quickly by Optimized High-Dose Imatinib: Results From the Randomized CML-Study IV. *J. Clin. Oncol.* 2013.
12. Graham SM, Jørgensen HG, Allan E, et al. Primitive, quiescent, Philadelphia-positive stem cells from patients with chronic myeloid leukemia are insensitive to STI571 in vitro. *Blood*. 2002;99(1):319–325.
13. Corbin AS, Agarwal A, Loriaux M, et al. Human chronic myeloid leukemia stem cells are insensitive to imatinib despite inhibition of BCR-ABL activity. *J. Clin. Invest.* 2011;121(1):396–409.
14. Cortes J, O'Brien S, Kantarjian H. Discontinuation of imatinib therapy after achieving a molecular response. *Blood*. 2004;104(7):2204–2205.
15. Rousselot P, Huguet F, Réa D, et al. Imatinib mesylate discontinuation in patients with chronic myelogenous leukemia in complete molecular remission for more than 2 years. *Blood*. 2007;109(1):58–60.
16. Mahon F-X, Réa D, Guilhot J, et al. Discontinuation of imatinib in patients with chronic myeloid leukaemia who have maintained complete molecular remission for at least 2 years: the prospective, multicentre Stop Imatinib (STIM) trial. *Lancet Oncol.* 2010;11(11):1029–1035.
17. Ross DM, Branford S, Seymour JF, et al. Safety and efficacy of imatinib cessation for CML patients with stable undetectable minimal residual disease: results from the TWISTER study. *Blood*. 2013;122(4):515–522.
18. Rousselot P, Charbonnier A, Cony-Makhoul P, et al. Loss of Major Molecular Response As a Trigger for Restarting Tyrosine Kinase Inhibitor Therapy in Patients With Chronic-Phase Chronic Myelogenous Leukemia Who Have Stopped Imatinib After Durable Undetectable Disease. *J. Clin. Oncol.* 2013;32(5):424–430.
19. Branford S, Yeung DT, Ross DM, et al. Early molecular response and female sex strongly predict stable undetectable BCR-ABL1, the criteria for imatinib discontinuation in patients with CML. *Blood*. 2013;121(19):3818–3824.
20. Talpaz M, Kantarjian HM, McCredie KB, et al. Clinical investigation of human alpha interferon in chronic myelogenous leukemia. *Blood*. 1987;69(5):1280–1288.
21. Talpaz M, Kantarjian HM, McCredie K, et al. Hematologic remission and cytogenetic improvement induced by recombinant human interferon alpha A in chronic myelogenous leukemia. *N. Engl. J. Med.* 1986;314(17):1065–1069.
22. Hehlmann R, Heimpel H, Hasford J, et al. Randomized comparison of interferon-alpha with busulfan and hydroxyurea in chronic myelogenous leukemia. The German CML Study Group. *Blood*. 1994;84(12):4064–4077.
23. Guilhot F, Chastang C, Michallet M, et al. Interferon alfa-2b combined with cytarabine versus interferon alone in chronic myelogenous leukemia. French Chronic Myeloid Leukemia Study Group. *N. Engl. J. Med.* 1997;337(4):223–229.
24. Hughes TP, Kaeda J, Branford S, et al. Frequency of major molecular responses to imatinib or interferon alfa plus cytarabine in newly diagnosed chronic myeloid leukemia. *N. Engl. J. Med.* 2003;349(15):1423–1432.
25. Hochhaus A, Reiter A, Saußele S, et al. Molecular heterogeneity in complete cytogenetic responders after interferon-alpha therapy for chronic myelogenous leukemia: low levels of minimal residual disease are associated with continuing remission. German CML Study Group and the UK MRC CML Study Group. *Blood*. 2000;95(1):62–66.
26. Bonifazi F, de Vivo A, Rosti G, et al. Chronic myeloid leukemia and interferon-alpha: a study of complete cytogenetic responders. *Blood*. 2001;98(10):3074–3081.
27. Mahon FX, Delbrel X, Cony-Makhoul P, et al. Follow-up of complete cytogenetic remission in patients with chronic myeloid leukemia after cessation of interferon alfa. *J. Clin. Oncol.* 2002;20(1):214–220.
28. Mauro MJ, Druker BJ, Maziarz RT. Divergent clinical outcome in two CML patients who discontinued imatinib therapy after achieving a molecular remission. *Leukemia Res*. 2004;28 Suppl 1:S71–3.
29. Merante S, Orlandi E, Bernasconi P, et al. Outcome of four patients with chronic myeloid leukemia after imatinib mesylate discontinuation. *Haematologica*. 2005;90(7):979–981.
30. Burchert A, Wölfl S, Schmidt M, et al. Interferon-alpha, but not the ABL-kinase inhibitor imatinib (STI571), induces expression of myeloblastin and a specific T-cell response in chronic myeloid leukemia. *Blood*. 2003;101(1):259–264.
31. Molldrem JJ, Lee PP, Wang C, et al. Evidence that specific T lymphocytes may participate in the elimination of chronic myelogenous leukemia. *Nat. Med.* 2000;6(9):1018–1023.

32. Molldrem JJ, Lee PP, Kant S, et al. Chronic myelogenous leukemia shapes host immunity by selective deletion of high-avidity leukemia-specific T cells. *J. Clin. Invest.* 2003;111(5):639–647.

33. Kreutzman A, Rohon P, Faber E, et al. Chronic myeloid leukemia patients in prolonged remission following interferon-α monotherapy have distinct cytokine and oligoclonal lymphocyte profile. *PLoS ONE*. 2011;6(8):e23022.

34. Baccarani M, Martinelli G, Rosti G, et al. Imatinib and pegylated human recombinant interferon-alpha2b in early chronic-phase chronic myeloid leukemia. *Blood*. 2004;104(13):4245–4251.

35. Preudhomme C, Guilhot J, Nicolini FE, et al. Imatinib plus peginterferon alfa-2a in chronic myeloid leukemia. *N. Engl. J. Med.* 2010;363(26):2511–2521.

36. Simonsson B, Gedde-Dahl T, Markevärn B, et al. Combination of pegylated IFN-α2b with imatinib increases molecular response rates in patients with low- or intermediate-risk chronic myeloid leukemia. *Blood*. 2011;118(12):3228–3235.

37. Hehlmann R, Lauseker M, Jung-Munkwitz S, et al. Tolerability-adapted imatinib 800 mg/d versus 400 mg/d versus 400 mg/d plus interferon-α in newly diagnosed chronic myeloid leukemia. *J. Clin. Oncol.* 2011;29(12):1634–1642.

38. Nicolini FE, Etienne G, Dubruille V, et al. Nilotinib and peginterferon alfa-2a for newly diagnosed chronic-phase chronic myeloid leukaemia (NiloPeg): a multicentre, non-randomised, open-label phase 2 study. *Lancet Haematology*. 2015;2:e37–e46.

39. Burchert A, Müller MC, Kostrewa P, et al. Sustained molecular response with interferon alfa maintenance after induction therapy with imatinib plus interferon alfa in patients with chronic myeloid leukemia. *J. Clin. Oncol.* 2010;28(8):1429–1435.

40. Burchert A, Saußele S, Eigendorff E, et al. Interferon alpha 2 (IFN) maintenance therapy may enable high rates of treatment discontinuation in chronic myeloid leukemia (CML). *Leukemia*. 2015;29:1331–1335.

41. Baccarani M, Rosti G, de Vivo A, et al. A randomized study of interferon-alpha versus interferon-alpha and low-dose arabinosyl cytosine in chronic myeloid leukemia. *Blood*. 2002;99(5):1527–1535.

42. Lipton JH, Khoroshko N, Golenkov A, et al. Phase II, randomized, multicenter, comparative study of peginterferon-alpha-2a (40 kD) (Pegasys) versus interferon alpha-2a (Roferon-A) in patients with treatment-naïve, chronic-phase chronic myelogenous leukemia. *Leuk. Lymphoma*. 2007;48(3):497–505.

43. Koskenvesa P, Kreutzman A, Rohon P, et al. Imatinib and pegylated IFN-α2b discontinuation in first-line chronic myeloid leukemia patients following a major molecular response. *Eur. J. Haematol.* 2014;92(5):413–420.

44. Faderl S, Talpaz M, Estrov Z, Kantarjian HM. Chronic myelogenous leukemia: biology and therapy. *Ann. Intern. Med.* 1999;131(3):207–219.

45. Saußele S, Richter J, Hochhaus A, Mahon FX. The concept of treatment-free remission in chronic myeloid leukemia. *Leukemia*. 2016;30(8):1638–1647.

46. Mahon F-X, Richter J, Guilhot J, et al. Interim Analysis of a Pan European Stop Tyrosine Kinase Inhibitor Trial in Chronic Myeloid Leukemia : The EURO-SKI study. *Blood*. 2014;124(21):151.

47. Kluin-Nelemans HC, Kester MG, van deCorput L, et al. Correction of abnormal T-cell receptor repertoire during interferon-alpha therapy in patients with hairy cell leukemia. *Blood*. 1998;91(11):4224–4231.

48. Kantarjian HM, O'Brien S, Cortes JE, et al. Complete cytogenetic and molecular responses to interferon-alpha-based therapy for chronic myelogenous leukemia are associated with excellent long-term prognosis. *Cancer*. 2003;97(4):1033–1041.

49. Hasford J, Baccarani M, Hoffmann V, et al. Predicting complete cytogenetic response and subsequent progression-free survival in 2060 patients with CML on imatinib treatment: the EUTOS score. *Blood*. 2011;118(3):686–692.

50. Sokal JE, Cox EB, Baccarani M, et al. Prognostic discrimination in “good-risk” chronic granulocytic leukemia. *Blood*. 1984;63(4):789–799.

51. Hasford J, Pfirrmann M, Hehlmann R, et al. A new prognostic score for survival of patients with chronic myeloid leukemia treated with interferon alfa. Writing Committee for the Collaborative CML Prognostic Factors Project Group. *J. Natl. Cancer Inst.* 1998;90(11):850–858.

52. Pfirrmann M, Lauseker M, Hoffmann VS, Hasford J. Prognostic scores for patients with chronic myeloid leukemia under particular consideration of competing causes of death. *Ann. Hematol.* 2015;94 Suppl 2:S209–18.

53. Pfirrmann M, Baccarani M, Saußele S, et al. Prognosis of long-term survival considering disease-specific death in patients with chronic myeloid leukemia. *Leukemia*. 2016;30(1):48–56.

54. Rasimas J, Katsounas A, Raza H, et al. Gene expression profiles predict emergence of psychiatric adverse events in HIV/HCV-coinfected patients on interferon-based HCV therapy. *J. Acquir. Immune Defic. Syndr.* 2012;60(3):273–281.

55. Tateno M, Honda M, Kawamura T, Honda H, Kaneko S. Expression profiling of peripheral-blood mononuclear cells from patients with chronic hepatitis C undergoing interferon therapy. *J. Infect. Dis.* 2007;195(2):255–267.

56. Zimmerer JM, Lesinski GB, Ruppert AS, et al. Gene expression profiling reveals similarities between the in vitro and in vivo responses of immune effector cells to IFN-alpha. *Clin. Cancer Res.* 2008;14(18):5900–5906.

57. Osinusi A, Rasimas JJ, Bishop R, et al. HIV/Hepatitis C virus-coinfected virologic responders to pegylated interferon and ribavirin therapy more frequently incur interferon-related adverse events than nonresponders do. *J. Acquir. Immune Defic. Syndr.* 2010;53(3):357–363.

58. Hasham A, Zhang W, Lotay V, et al. Genetic analysis of interferon induced thyroiditis (IIT): evidence for a key role for MHC and apoptosis related genes and pathways. *J Autoimmun*. 2013;44:61–70.

59. Pfirrmann M, Hochhaus A, Lauseker M, Saussele S, Hehlmann R, Hasford J. Recommendations to meet statistical challenges arising from endpoints beyond overall survival in clinical trials on chronic myeloid leukemia. Leukemia. 2011;25(9):1433-1438.

60. Putter H, Fiocco M, Geskus RB. Tutorial in biostatistics: competing risks and multi-state models. Statistics in Medicine. 2007;26(11):2389-2430.
