# Supplementary material for: Randomized phase 3 trial of Ropeginterferon alfa-2b versus surveillance after tyrosine kinase inhibitor discontinuation in chronic myeloid leukemia (ENDURE/CML-IX)
Source: Leukemia. 2026 Jan 12;40(2):410–7. doi: 10.1038/s41375-025-02859-1 (PMC12875868; doi:10.1038/s41375-025-02859-1)
Supplement: Supplementary file 2 — Study protocol - Amendment [file 41375_2025_2859_MOESM2_ESM.pdf]

Amendment No. 2 und 3

Document change history to
to Clinical Study Protocol Version V05F and V06F

EFFICACY AND SAFETY OF PEGYLATED-PROLINE- INTERFERON ALPHA 2B (AOP2014) IN MAINTAINING DEEP MOLECULAR REMISSIONS IN PATIENTS WITH CHRONIC MYELOID LEUKEMIA (CML) WHO DISCONTINUE ABL-KINASE INHIBITOR THERAPY -
A RANDOMIZED PHASE II, MULTICENTER TRIAL
WITH POST-STUDY FOLLOW-UP

**Short Title:**	**ENDURE-CML (CML-IX study)**
**Protocol Code**	KKS-227
**EudraCT Number:**	2016-001030-94
**Clinical Trial Register Number:**	NCT03117816
**Investigational Product:**	Pegylated-Proline-interferon alpha-2b (AOP2014)
**Sponsor:**	Philipps University Marburg<br>Biegenstr. 10<br>D-35037 Marburg
**Coordinating Investigator:**	Prof. Dr. Andreas Burchert<br>Department of Hematology/Oncology/Immunology<br>University Hospital Marburg<br>Baldingerstrasse<br>D-35043 Marburg

This amendment includes two changes to the protocol.
Both will be included in the new version of the Clinical Study Protocol V06F dated 07.09.2018

Amendment 2 to Clinical Study Protocol V04F dated 04.12.2017
was included in the new version of the Clinical Study Protocol V05F dated 20.07.2018.

This version was approved by the Competent Authority (BfArM) in Germany at 03.09.2018,
but Ethical Review required changes, that were included in the next version.

Amendment 3 to Clinical Study Protocol V05F dated 20.07.2018 was included in the new version of the Clinical Study Protocol V06F dated 07.09.2018

Amendment No. 2

to Clinical Study Protocol V04F dated 04.12.2017

EFFICACY AND SAFETY OF PEGYLATED-PROLINE- INTERFERON ALPHA 2B (AOP2014) IN MAINTAINING DEEP MOLECULAR REMISSIONS IN PATIENTS WITH CHRONIC MYELOID LEUKEMIA (CML) WHO DISCONTINUE ABL-KINASE INHIBITOR THERAPY -
A RANDOMIZED PHASE II, MULTICENTER TRIAL
WITH POST-STUDY FOLLOW-UP

**Short Title:**	**ENDURE-CML (CML-IX study)**
**Protocol Code**	KKS-227
**EudraCT Number:**	2016-001030-94
**Clinical Trial Register Number:**	NCT03117816
**Investigational Product:**	Pegylated-Proline-interferon alpha-2b (AOP2014)
**Sponsor:**	Philipps University Marburg<br>Biegenstr. 10<br>D-35037 Marburg
**Coordinating Investigator:**	Prof. Dr. Andreas Burchert<br>Department of Hematology/Oncology/Immunology<br>University Hospital Marburg<br>Baldingerstrasse<br>D-35043 Marburg
**Date:**	24.07.2018
**Version:**	V02F

This amendment will be included in the new version of the Clinical Study Protocol V05F dated 20.07.2018

This amendment document and all its including parts are property of Prof. Dr. A. Burchert, Prof. Dr. A. Hochhaus, Prof Dr. Susanne Saußele on behalf of the CML study group / CML study alliance, the biometrician PD Dr. Pfirrmann and KKS Marburg. It is at confidential disposal to the members of the study team in the participating centers. The use of such confidential information must be restricted to the recipient for the agreed purpose and must not be disclosed, published or otherwise communicated to any unauthorized person, for any reason, in any form whatsoever without prior written approval of the parties stated above.

Description of the protocol amendment

☒ Substantial amendment / ☐ Non-Substantial amendment

Documents, which need to be modified because of this amendment:
- The study protocol
- The German Synopsis
- Modul 1

Overview of the amended protocol sections and reason for changes:

Spelling and typing errors as well as formatting changes are not listed.

**Page / section** (according to clinical study protocol V04F)	**Modification**	**Reason**
1-7	Front pages,<br>list of study personnel	Change of names, titles, and contact details; as well as specifications for other European countries
14,15 (Synopsis)<br><br>35 (9.4)	Enhanced number of trial sites<br>Multinational Trial Design	We would like to include more trial sites in the recruitment of patients for our clinical trial from previously 20 trial sites in Germany to 25-30 trial sites in Europe.<br>The trial is planned to be multinational in Europe.
16 (Synopsis),<br>33 - 34 (9.2)	Inclusion and exclusion criteria	Inclusion criterion no. 5:<br>The wording was changed to allow other PCR Laboratories for participating European countries.
19 (Synopsis),<br>30 (4.2)	Clarification on secondary safety endpoint	The wording was changed
21 (Table 1)	Table 1.2 with study specific procedures for Arm A	Missing timepoints were added to assure comparability with Arm B
35 (10.2)	Update of section 10.2 “Potential Toxicity in Patients”	Update due to new information
41	Labeling, Drug Supply Procedures	The wording was changed to allow other participating European countries.
50	Different procedure in case of withdrawal of consent	The wording was changed to continue TKI pausing on the decision of the investigator:<br>“In case of withdrawal of informed consent TKI treatment should be re-commenced unless the treating physician recommends to the patient to continue pausing the TKI off study until molecular relapse (loss of MMR) occurs.”
51 (14)	Revision of the safety section	Revision was done due to an update of European Guidelines and corrected wording.
58	Section on Quality of life assessment	The section was deleted, because no new information was given as in section 12.2.16
62 (17.2)	Section Data Safety and Monitoring Committee (DSMC)	Revision by KKS

**Page / section** (according to clinical study protocol V04F)	**Modification**	**Reason**
63 (17.3.1, 17.4)<br>64 (19.1, 19.2)<br>65 (19.4, 19.5)<br>66 (19.6)<br>67 (19.9, 21)	Specification of procedures for Germany and other participating European countries	The procedures were specified for Germany. Details concerning the patient insurance for other participation European countries are outlined in a separate Group Specific Appendix (GSA).

Consequences and risks for study subjects: All changes have no impact on the safety of the study subjects.

No further action is required concerning subjects treated under ENDURE (CML-IX Study) according to the current Amendment No. 2 and implementation of the new Clinical Study Protocol Version V05F dated 2018-07-20

For details of the changes listed above, please refer to the tracked changes document (ENDURE_CML_Study Protocol_V05F_2018-07-20_tracked changes).

Amendment No. 3

to Clinical Study Protocol V05F dated 20.07.2018

EFFICACY AND SAFETY OF PEGYLATED-PROLINE- INTERFERON ALPHA 2B (AOP2014) IN MAINTAINING DEEP MOLECULAR REMISSIONS IN PATIENTS WITH CHRONIC MYELOID LEUKEMIA (CML) WHO DISCONTINUE ABL-KINASE INHIBITOR THERAPY -
A RANDOMIZED PHASE II, MULTICENTER TRIAL
WITH POST-STUDY FOLLOW-UP

**Short Title:**	**ENDURE-CML (CML-IX study)**
**Protocol Code**	KKS-227
**EudraCT Number:**	2016-001030-94
**Clinical Trial Register Number:**	NCT03117816
**Investigational Product:**	Pegylated-Proline-interferon alpha-2b (AOP2014)
**Sponsor:**	Philipps University Marburg<br>Biegenstr. 10<br>D-35037 Marburg
**Coordinating Investigator:**	Prof. Dr. Andreas Burchert<br>Department of Hematology/Oncology/Immunology<br>University Hospital Marburg<br>Baldingerstrasse<br>D-35043 Marburg
**Date:**	07.09.2018
**Version:**	V03F

This amendment will be included in the new version of the Clinical Study Protocol V06F dated 07.09.2018

This amendment document and all its including parts are property of Prof. Dr. A. Burchert, Prof. Dr. A. Hochhaus, Prof Dr. Susanne Saußele on behalf of the CML study group / CML study alliance, the biometrician PD Dr. Pfirrmann and KKS Marburg. It is at confidential disposal to the members of the study team in the participating centers. The use of such confidential information must be restricted to the recipient for the agreed purpose and must not be disclosed, published or otherwise communicated to any unauthorized person, for any reason, in any form whatsoever without prior written approval of the parties stated above.

Description of the protocol amendment

☒ Substantial amendment / ☐ Non-Substantial amendment

Documents, which need to be modified because of this amendment:

- The study protocol
- The German Synopsis
- The Patient Information and Informed Consent Form
- The electronic case report forms (eCRFs)
- Modul 1

Overview of the amended protocol sections and reason for changes:
Spelling and typing errors as well as formatting changes are not listed.

**Page / section** (according to clinical study protocol V06F)	**Modification**	**Reason**
1-7	Front pages	Change of version and dates
15 (Synopsis)<br><br>45 (12.1.3)	Comparator Arm B	A patient in Arm B will receive no further *CML* treatment after one month.<br><br>**Patients randomized into arm B**, will discontinue TKI one month after randomization. From this moment patient will receive no further *CML* treatment.
17,18 (Synopsis),<br><br>35 (9.3)	Exclusion criteria Added due to new information provided with new IB 11.0	Exclusion criterion no. 3 was amended to include transplant recipients<br>Exclusion criterion no. 10 was added: End stage renal disease (GFR <15 ml/min)<br>Exclusion criterion no. 12 was added: Uncontrolled diabetes mellitus<br>Exclusion criterion no. 15 was added: Uncontrolled hypertension
23 (Legend)	Legend to table 1 and table 2	The headline corrected and serum tests were mentioned additionally (point 4) to avoid, that they would be forgotten.
40 (10.3.1.1.4)	Dose modifications	Dose modifications amended and warnings were added due to new information (IB 11.0)
43 (10.8.1.1)	Drug interactions	A section for drug interaction was added due to new information (IB 11.0)
48 (12.2.8.3)	Clarification blood tests for Clinical chemistry	Triglyceride and albumin were deleted at required clinical chemistry, as they were never mentioned before (e.g. at laboratory safety analyses)

Consequences and risks for study subjects: All changes have no impact on the safety of the study subjects.

No further action is required concerning subjects treated under ENDURE (CML-IX Study) according to the current Amendment No. 3 and implementation of the new Clinical Study Protocol Version V06F dated 2018-09-07.

For details of the changes listed above on Amendments 2 and 3, please refer to the tracked changes document tracking the changes from version V04F to version V06F (ENDURE-CML_tracked changes_Study Protocol V06F-V04F_Prüfplanamendment 2+3_2018-09-07).
