# Supplementary figures and images for: Randomized phase 3 trial of Ropeginterferon alfa-2b versus surveillance after tyrosine kinase inhibitor discontinuation in chronic myeloid leukemia (ENDURE/CML-IX)
Source: Leukemia. 2026 Jan 12;40(2):410–7. doi: 10.1038/s41375-025-02859-1 (PMC12875868; doi:10.1038/s41375-025-02859-1)

## Slide 1
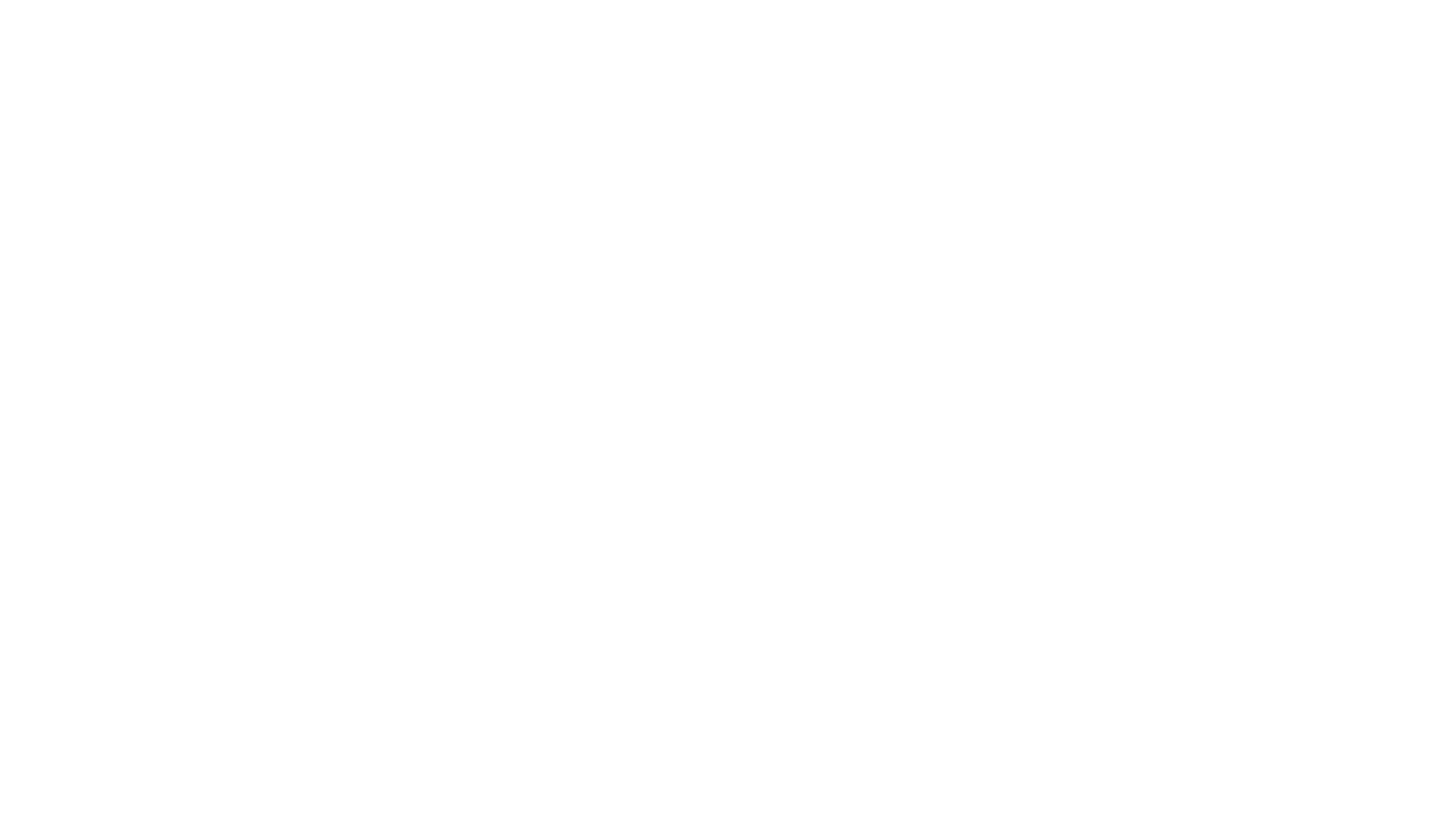

Supplement: Supplementary file 3 — Supplemental Figure 1 [file 41375_2025_2859_MOESM3_ESM.pptx]
