# Supplementary material for: Randomized phase 3 trial of Ropeginterferon alfa-2b versus surveillance after tyrosine kinase inhibitor discontinuation in chronic myeloid leukemia (ENDURE/CML-IX)
Source: Leukemia. 2026 Jan 12;40(2):410–7. doi: 10.1038/s41375-025-02859-1 (PMC12875868; doi:10.1038/s41375-025-02859-1)
Supplement: Supplementary file 4 — Supplemental Tables [file 41375_2025_2859_MOESM4_ESM.docx]

**Supplemental Tables**


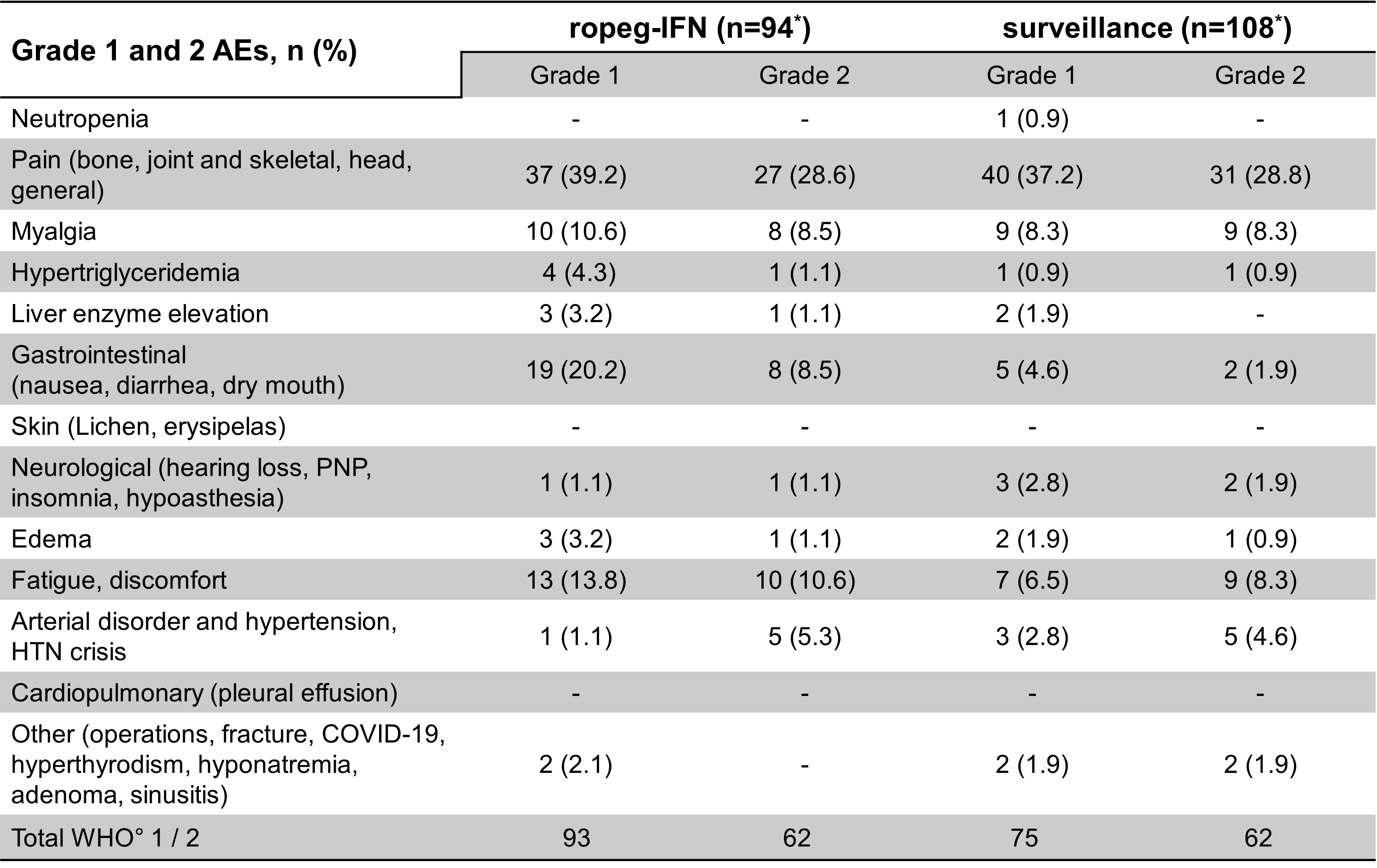


### **Supplemental Table 1. Incidence of low-grade adverse events (safety population)**

Note: All data are presented as No. (%) unless otherwise indicated.

**Abbreviations:** COVID, coronavirus disease; PNP, peripheral neuropathic pain; ropeg-IFN, ropeginterferon alfa-2b.


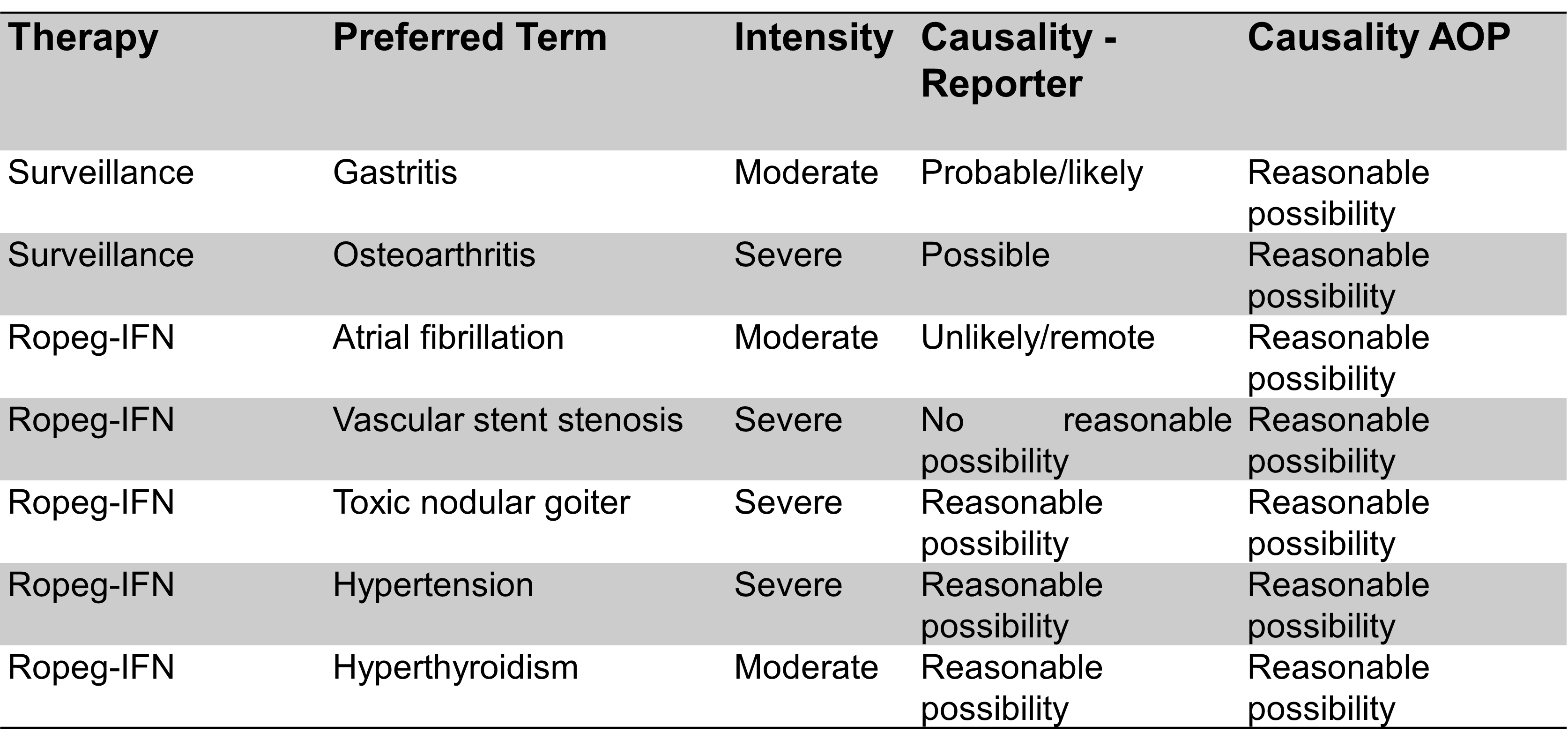


### **Supplemental Table 2. Serious adverse events (SAEs)**

**Abbreviations:** ropeg-IFN, ropeginterferon alfa-2b.
